# Supplementary material for: Hitac: a hierarchical taxonomic classifier for fungal ITS sequences compatible with QIIME2
Source: BMC Bioinformatics. 2024 Jul 2;25:228. doi: 10.1186/s12859-024-05839-x (PMC11220968; doi:10.1186/s12859-024-05839-x)
Supplement: Supplementary file 1 — Supporting information text, Figures S1–S4 and tables S1–S92 [file 12859_2024_5839_MOESM1_ESM.pdf]

bioRxiv

Supporting Information for

HiTaC: a hierarchical taxonomic classifier for fungal ITS sequences compatible with QIIME2

Fábio Miranda, Vasco Azevedo, Rommel Ramos, Bernhard Renard and Vitor Piro

Corresponding Author: Vitor Piro.
E-mail: vitor.piro@fu-berlin.de

Table of contents

Supporting Information Text 4

Evaluation 4
Datasets . . . 4
Evaluation Metrics . . . 4
Default metrics from the TAXXI benchmark . . . 4
Standard machine learning metrics . . . 5
Hierarchical machine learning metrics . . . 6
Resources benchmark . . . 6

List of Figures

S1 Depiction of imbalanced training set . . . 7
S2 Accuracies for the top 5 methods . . . 8
S3 Under-classification versus over-classification rates . . . 9
S4 Misclassification rate results . . . 10

List of Tables

S1 List of software available in the TAXXI benchmark . . . 11
S2 List of training datasets available in the TAXXI benchmark . . . 12
S3 List of test datasets available in the TAXXI benchmark . . . 13
S4 Commands and parameters to run BLCA. . . . 14
S5 Commands and parameters to run BTOP. . . . 15
S6 Commands and parameters to run CT1. . . . 16
S7 Commands and parameters to run CT2. . . . 17
S8 Commands and parameters to run HiTaC. . . . 18
S9 Commands and parameters to run HiTaC_Filter. . . . 19
S10 Commands and parameters to run KNN. . . . 20
S11 Commands and parameters to run KTOP. . . . 21
S12 Commands and parameters to run Metaxa2. . . . 22
S13 Commands and parameters to run Microclass. . . . 23
S14 Commands and parameters to run NBC. . . . 24
S15 Commands and parameters to run Q1. . . . 25
S16 Commands and parameters to run Q2_BLAST. . . . 26
S17 Commands and parameters to run Q2_SK. . . . 27
S18 Commands and parameters to run Q2_VS. . . . 28
S19 Commands and parameters to run RDP. . . . 29
S20 Commands and parameters to run SINTAX. . . . 30
S21 Commands and parameters to run SPINGO. . . . 31
S22 Commands and parameters to run TOP. . . . 32

S23 Resources benchmark computed for the dataset SP RDP ITS 90. . . . 33
S24 Resources benchmark computed for the dataset SP RDP ITS 95. . . . 34
S25 Resources benchmark computed for the dataset SP RDP ITS 97. . . . 35
S26 Resources benchmark computed for the dataset SP RDP ITS 99. . . . 36
S27 Resources benchmark computed for the dataset SP RDP ITS 100. . . . 37
S28 Hierarchical metrics computed for the dataset SP RDP ITS 90. . . . 38
S29 Hierarchical metrics computed for the dataset SP RDP ITS 95. . . . 39
S30 Hierarchical metrics computed for the dataset SP RDP ITS 97. . . . 40
S31 Hierarchical metrics computed for the dataset SP RDP ITS 99. . . . 41
S32 Hierarchical metrics computed for the dataset SP RDP ITS 100. . . . 42
S33 TAXXI metrics computed for the dataset SP RDP ITS 90 at the phylum level. . . . 43
S34 TAXXI metrics computed for the dataset SP RDP ITS 90 at the class level. . . . 44
S35 TAXXI metrics computed for the dataset SP RDP ITS 90 at the order level. . . . 45
S36 TAXXI metrics computed for the dataset SP RDP ITS 90 at the family level. . . . 46
S37 TAXXI metrics computed for the dataset SP RDP ITS 90 at the genus level. . . . 47
S38 TAXXI metrics computed for the dataset SP RDP ITS 90 at the species level. . . . 48
S39 TAXXI metrics computed for the dataset SP RDP ITS 95 at the phylum level. . . . 49
S40 TAXXI metrics computed for the dataset SP RDP ITS 95 at the class level. . . . 50
S41 TAXXI metrics computed for the dataset SP RDP ITS 95 at the order level. . . . 51
S42 TAXXI metrics computed for the dataset SP RDP ITS 95 at the family level. . . . 52
S43 TAXXI metrics computed for the dataset SP RDP ITS 95 at the genus level. . . . 53
S44 TAXXI metrics computed for the dataset SP RDP ITS 95 at the species level. . . . 54
S45 TAXXI metrics computed for the dataset SP RDP ITS 97 at the phylum level. . . . 55
S46 TAXXI metrics computed for the dataset SP RDP ITS 97 at the class level. . . . 56
S47 TAXXI metrics computed for the dataset SP RDP ITS 97 at the order level. . . . 57
S48 TAXXI metrics computed for the dataset SP RDP ITS 97 at the family level. . . . 58
S49 TAXXI metrics computed for the dataset SP RDP ITS 97 at the genus level. . . . 59
S50 TAXXI metrics computed for the dataset SP RDP ITS 97 at the species level. . . . 60
S51 TAXXI metrics computed for the dataset SP RDP ITS 99 at the phylum level. . . . 61
S52 TAXXI metrics computed for the dataset SP RDP ITS 99 at the class level. . . . 62
S53 TAXXI metrics computed for the dataset SP RDP ITS 99 at the order level. . . . 63
S54 TAXXI metrics computed for the dataset SP RDP ITS 99 at the family level. . . . 64
S55 TAXXI metrics computed for the dataset SP RDP ITS 99 at the genus level. . . . 65
S56 TAXXI metrics computed for the dataset SP RDP ITS 99 at the species level. . . . 66
S57 TAXXI metrics computed for the dataset SP RDP ITS 100 at the phylum level. . . . 67
S58 TAXXI metrics computed for the dataset SP RDP ITS 100 at the class level. . . . 68
S59 TAXXI metrics computed for the dataset SP RDP ITS 100 at the order level. . . . 69
S60 TAXXI metrics computed for the dataset SP RDP ITS 100 at the family level. . . . 70
S61 TAXXI metrics computed for the dataset SP RDP ITS 100 at the genus level. . . . 71
S62 TAXXI metrics computed for the dataset SP RDP ITS 100 at the species level. . . . 72
S63 Machine learning metrics computed for the dataset SP RDP ITS 90 at the phylum level. . . . 73
S64 Machine learning metrics computed for the dataset SP RDP ITS 90 at the class level. . . . 74
S65 Machine learning metrics computed for the dataset SP RDP ITS 90 at the order level. . . . 75
S66 Machine learning metrics computed for the dataset SP RDP ITS 90 at the family level. . . . 76
S67 Machine learning metrics computed for the dataset SP RDP ITS 90 at the genus level. . . . 77
S68 Machine learning metrics computed for the dataset SP RDP ITS 90 at the species level. . . . 78
S69 Machine learning metrics computed for the dataset SP RDP ITS 95 at the phylum level. . . . 79
S70 Machine learning metrics computed for the dataset SP RDP ITS 95 at the class level. . . . 80
S71 Machine learning metrics computed for the dataset SP RDP ITS 95 at the order level. . . . 81
S72 Machine learning metrics computed for the dataset SP RDP ITS 95 at the family level. . . . 82
S73 Machine learning metrics computed for the dataset SP RDP ITS 95 at the genus level. . . . 83
S74 Machine learning metrics computed for the dataset SP RDP ITS 95 at the species level. . . . 84
S75 Machine learning metrics computed for the dataset SP RDP ITS 97 at the phylum level. . . . 85
S76 Machine learning metrics computed for the dataset SP RDP ITS 97 at the class level. . . . 86
S77 Machine learning metrics computed for the dataset SP RDP ITS 97 at the order level. . . . 87
S78 Machine learning metrics computed for the dataset SP RDP ITS 97 at the family level. . . . 88
S79 Machine learning metrics computed for the dataset SP RDP ITS 97 at the genus level. . . . 89
S80 Machine learning metrics computed for the dataset SP RDP ITS 97 at the species level. . . . 90
S81 Machine learning metrics computed for the dataset SP RDP ITS 99 at the phylum level. . . . 91
S82 Machine learning metrics computed for the dataset SP RDP ITS 99 at the class level. . . . 92
S83 Machine learning metrics computed for the dataset SP RDP ITS 99 at the order level. . . . 93

S84 Machine learning metrics computed for the dataset SP RDP ITS 99 at the family level. 94
S85 Machine learning metrics computed for the dataset SP RDP ITS 99 at the genus level. 95
S86 Machine learning metrics computed for the dataset SP RDP ITS 99 at the species level. 96
S87 Machine learning metrics computed for the dataset SP RDP ITS 100 at the phylum level. 97
S88 Machine learning metrics computed for the dataset SP RDP ITS 100 at the class level. 98
S89 Machine learning metrics computed for the dataset SP RDP ITS 100 at the order level. 99
S90 Machine learning metrics computed for the dataset SP RDP ITS 100 at the family level. 100
S91 Machine learning metrics computed for the dataset SP RDP ITS 100 at the genus level. 101
S92 Machine learning metrics computed for the dataset SP RDP ITS 100 at the species level. 102

Supporting Information Text

Evaluation

The TAXXI benchmark (1) was employed to facilitate the comparison between HiTaC and seventeen other similar software previously available in the benchmark (Table S1), some of which are similarity based approaches, while others are alignment free. We relied on this benchmark because it mimics more accurately real world scenarios including novel sequences, as well as its diversity in software, reference databases and use of real environmental data. Furthermore, the TAXXI benchmark assesses the accuracy using cross-validation by identity, a strategy which models the variation in distances between query sequences and the closest entry in reference databases. In this approach, a 90% identity enforces sequences to only belong to species that are absent from the training set, while a 95% identity allows for a mix of present and absent species. In other words, the cross-validation by identity enables the creation of datasets whose lowest taxonomic ranks were nonexistent in the training data while still knowing the ground truth, which allows for a better evaluation on how a classifier behaves when trying to predict novel sequences.

Datasets. We adopted five datasets containing both training and test data, which were previously available in the TAXXI benchmark (1). These datasets were selected because they were the only ones with taxonomic annotations of fungal ITS sequences up to species level. Although there were other five datasets available in this benchmark possessing fungal ITS sequences, we noticed that they were copies of the same datasets we selected, except that they were missing the species level annotation in the training datasets and ground truth. Thus, there was no added value in using these other datasets in our evaluation.

The fungal ITS datasets selected from the TAXXI benchmark were based on real environmental data, publicly available on NCBI (2), RDP (3), the Warcup fungal ITS training set v2 (WITS) (4), UNITE (5) and other *in vivo* samples. They were created through cross-validation by identity, hence the identity between the query sequences and the closest entry in a reference database varied on them, ranging from 90% to 100% (1) (Tables S2-S3). In practice, an identity of 90% means that the training data does not contain any of the labels that are in the test dataset at the species level, but most labels are known at the genus level. The known taxa at the species level increase with identity, i.e., all species are present in the training data when the identity is 100%.

It is also important to mention that the training datasets are extremely imbalanced. For example, among the seven existing phyla on dataset SP RDP ITS 90, Ascomycota and Basidiomycota contain approximately 52% and 44% of total instances, respectively (Fig. S1). A similar pattern can be observed in the other datasets with higher identities.

Evaluation Metrics.

Default metrics from the TAXXI benchmark. These metrics, as well as scripts used to compute them, originated from the TAXXI benchmark (1):

- **Accuracy (ACC) -** Accounts for all test sequences predicted by the algorithm with names that are known and/or over-predicted, ranging from 0% (no correct predictions) to 100% (no errors);
- **Misclassification Rate (MCR) -** The percentage of known sequences incorrectly predicted;
- **Over-classification Rate (OCR) -** The percentage of unknown sequences assigned a label;
- **True Positive Rate (TPR) -** The percentage of known sequences correctly predicted;
- **Under-classification Rate (UCR) -** The percentage of known sequences that were not assigned a label.

Defining these metrics in mathematical terms, we have:

$$\text{ACC} = 100 \times \frac{\text{True Positive}}{\text{Known Sequences} + \text{Over-classification Errors}}$$

$$\text{MCR} = 100 \times \frac{\text{Misclassification Errors}}{\text{Known Sequences}}$$

$$\text{OCR} = 100 \times \frac{\text{Over-classification Errors}}{\text{Novel Sequences}}$$

$$\text{TPR} = 100 \times \frac{\text{True Positive}}{\text{Known Sequences}}$$

$$\text{UCR} = 100 \times \frac{\text{Under-classification Errors}}{\text{Known Sequences}}$$

where *known sequences* have labels in the training dataset and *novel sequences* do not have labels in the training dataset. A *true positive* is the number of correctly predicted sequences. *Under-classification errors* occur when too few ranks are predicted, while *over-classification errors* happen when too many ranks are predicted. Finally, *misclassification errors* occur when a known name is incorrectly predicted.

Standard machine learning metrics. These metrics and their respective implementations originated from the package scikit-learn (6):

- **Accuracy -** The fraction of correctly classified samples;
- **Balanced accuracy -** Accuracy counterpart planned to deal with imbalanced datasets, which is defined as the average of recall obtained on each class;
- **Precision -** Intuitively, precision is the ability of the classifier not to label a negative sample as positive, and it ranges from 0 (worst score) to 100 (best value);
- **Recall -** Intuitively, recall is the ability of the classifier to detect all positive samples;
- **F1-score -** The F1-score is the harmonic mean of the precision and recall, ranging from 0 (worst score) to 100 (best value);
- **Jaccard -** Quantifies the similarity between the set of predicted labels and the ground truth;
- **Micro, macro and weighted variants -** The Precision, Recall, F1-score and Jaccard were computed in three different ways, which are described below:

  - **Micro:** Computes metrics globally considering the overall true positives, false negatives and false positives;

  - **Macro:** Computes metrics locally for each label and determines their unweighted average without considering label imbalance;

  - **Weighted:** Computes metrics locally for each label and determines their average weight by support, that is, the total of correct instances for each label. In practice, this modifies "macro" to consider label imbalance, which can result in an F1-score that is not between precision and recall.

Defining these metrics in mathematical terms, we have:

$$\text{Accuracy}(y, \hat{y}) = 100 \times \frac{1}{n_{\text{samples}}} \sum_{i=0}^{n_{samples}-1} \mathbb{1}(\hat{y}_i = y_i)$$

where $\hat{y}_i$ is the predicted value of the i-th sample and y_i is the corresponding ground truth, while $\mathbb{1}$ is the indicator function.

$$\text{Balanced accuracy}(y, \hat{y}, w) = 100 \times \frac{1}{\sum \hat{w}_i} \sum_i \mathbb{1}(\hat{y}_i = y_i)\hat{w}_i$$

where $\hat{w}_i = \dfrac{w_i}{\sum_j \mathbb{1}(y_j = y_i) w_j}$ and w_i is the weight of the i-th sample.

$$\text{Precision}(y, \hat{y}) = 100 \times \frac{\text{True Positives}}{\text{True Positives} + \text{False Positives}}$$

$$\text{Recall}(y, \hat{y}) = 100 \times \frac{\text{True Positives}}{\text{True Positives} + \text{False Negatives}}$$

$$\text{F1-score}(y, \hat{y}) = 100 \times \frac{2 \times \text{Precision} \times \text{Recall}}{\text{Precision} + \text{Recall}}$$

$$\text{Jaccard}(y, \hat{y}) = 100 \times \frac{y \cap \hat{y}}{y \cup \hat{y}}$$

$$\text{Micro}(y, \hat{y}) = 100 \times M(y, \hat{y})$$

where M is the metric to compute for, i.e., precision, recall, F1-score or jaccard.

$$\text{Macro}(y, \hat{y}) = 100 \times \frac{1}{|L|} \sum_{l \in L} M(y_l, \hat{y}_l)$$

where L is the set of labels.

$$\text{Weighted}(y, \hat{y}) = 100 \times \frac{1}{\sum_{l \in L} |y_l|} \sum_{l \in L} |y_l| M(y_l, \hat{y}_l)$$

Hierarchical machine learning metrics. These metrics are adaptations of the previously introduced metrics of precision, recall and F1-score, but tailored to the hierarchical classification scenario. Their respective implementations originated from the package HiClass (7):

$$\text{Hierarchical precision } (hP) = 100 \times \frac{\sum_i |\alpha_i \cap \beta_i|}{\sum_i |\alpha_i|}$$

$$\text{Hierarchical recall } (hR) = 100 \times \frac{\sum_i |\alpha_i \cap \beta_i|}{\sum_i |\beta_i|}$$

$$\text{Hierarchical F1-score} = 100 \times \frac{2 \times hP \times hR}{hP + hR}$$

where α_i is the set containing the predicted labels for test example i and their respective ancestors, while β_i is the set containing the ground truth of test example i and their respective ancestors, with summations computed over all test examples.

Resources benchmark. We enhanced the TAXXI benchmark by porting it to Snakemake (8) in order to make it more reproducible, and we introduced a resources benchmark. The benchmark scripts and instructions on how to run it are available in our GitLab repository[1]. The benchmark was computed on a laptop running GNU/Linux with 16 GB physical memory and 12 cores provided by an AMD Ryzen™ 5 processor, where 12 cores were allocated for each method. The results from the benchmark are available in Tables S23-S27, while the commands and parameters executed for all software are available in Tables S4-S22.

[1] https://gitlab.com/dacs-hpi/hitac/-/tree/main/benchmark

Number of examples for each taxonomic category

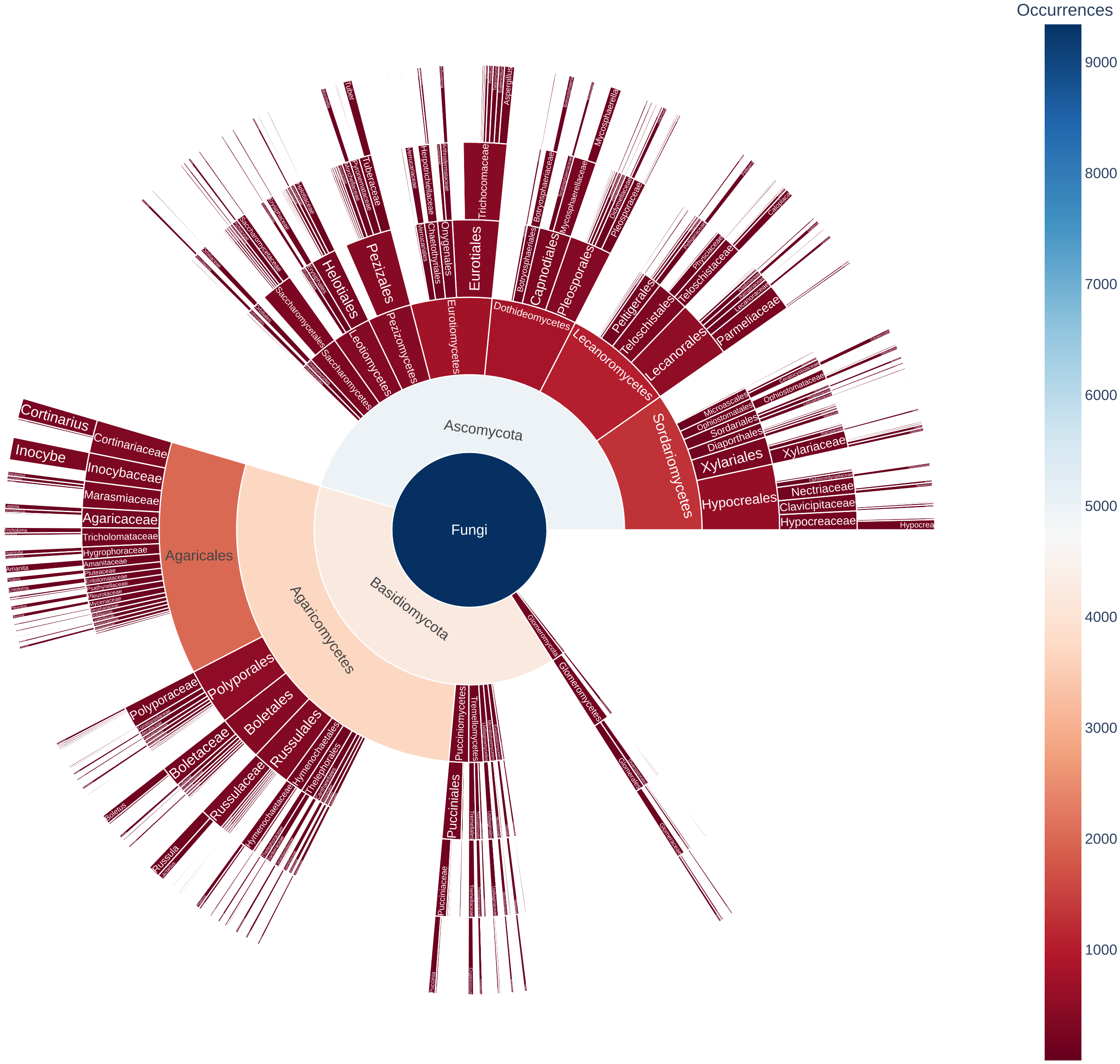

Fig. S1. Depiction of imbalanced training set. For instance, the phyla Ascomycota and Basidiomycota together contain approximately 96% of total instances between the seven existing phyla in dataset SP RDP ITS 90. In the remaining taxonomic ranks and datasets we can also observe similar patterns.

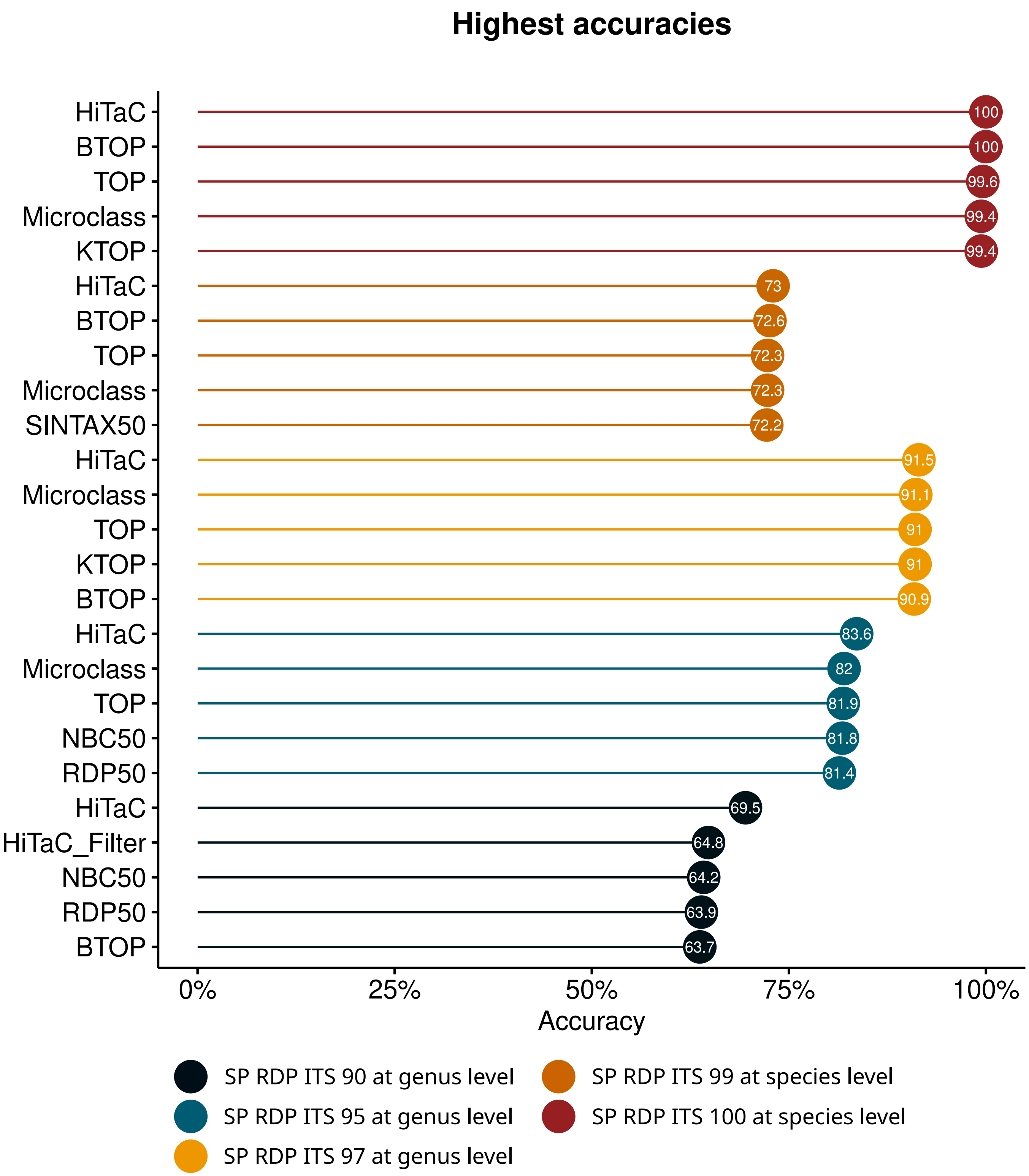

Fig. S2. Accuracies for the top 5 methods for datasets SP RDP ITS 90 at the genus level, SP RDP ITS 95 at the genus level, SP RDP ITS 97 at the genus level, SP RDP ITS 99 at the species level and SP RDP ITS 100 at the species level. The trend is that HiTaC achieved an accuracy higher or equal to top methods. For instance, HiTaC tied with the top method for the dataset with 100% identity, obtaining a perfect score. Moreover, for the datasets with 90-99% identity, HiTaC improved the accuracy upon top methods.

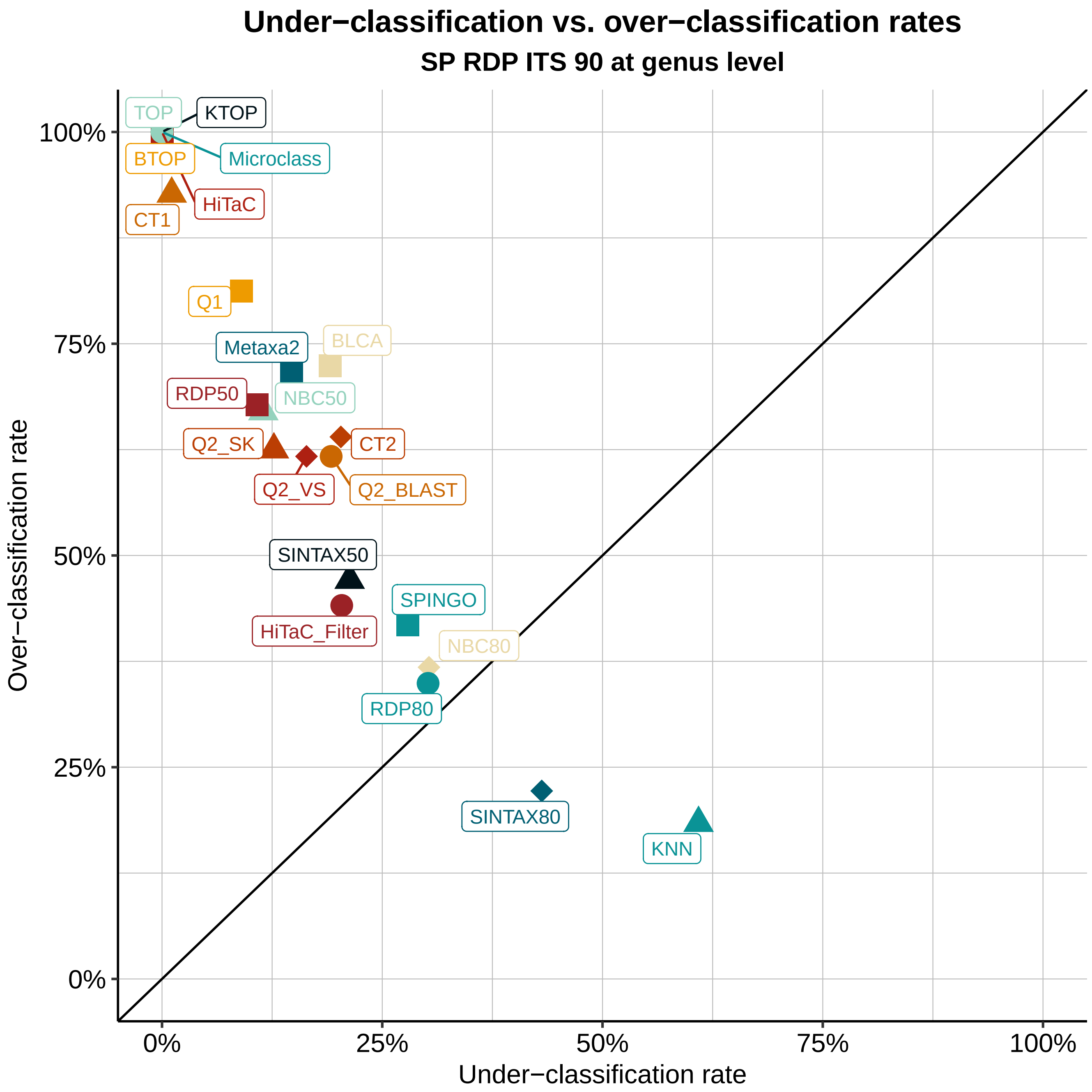

Fig. S3. Under-classification versus over-classification rates for dataset SP RDP ITS 90 at genus level. The introduction of the filter in HiTaC proved to be effective by sharply decreasing the over-classification rate from 100% to 44.1%. Contrarily, the filter slightly increased the under-classification rate from 0% to 20.4%, but it still ranked among the methods with the best trade-off between over-classification and under-classification rates, which are the methods closer to the diagonal on the bottom left. An ideal method should be able to classify all known sequences and leave novel sequences unclassified, thus avoiding both over-classification and under-classification errors.

Misclassification rate

Method	SP RDP ITS 90 genus level	SP RDP ITS 95 genus level	SP RDP ITS 97 genus level	SP RDP ITS 99 species level	SP RDP ITS 100 species level
BLCA	15.9	10.2	6.8	7.9	0.0
BTOP	26.6	15.2	8.4	11.3	0.0
CT1	27.9	16.1	10.6	28.5	8.7
CT2	19.0	10.6	11.3	0.9	1.1
HiTaC	19.7	12.9	7.7	10.6	0.0
HiTaC_Filter	10.3	8.3	5.0	2.7	0.0
KNN	3.3	1.9	1.9	0.0	0.0
KTOP	27.8	15.8	8.3	12.2	0.6
Metaxa2	19.7	10.6	7.3	2.8	0.0
Microclass	27.5	14.6	8.2	11.4	0.6
NBC50	17.6	13.1	7.4	13.3	Memory exceeded
NBC80	8.8	8.2	4.9	7.0	Memory exceeded
Q1	23.0	14.7	11.4	21.7	7.8
Q2_BLAST	19.4	12.4	11.1	1.7	1.3
Q2_SK	18.8	11.8	6.7	8.4	0.8
Q2_VS	19.5	11.0	10.4	2.1	1.3
RDP50	18.6	12.9	7.1	13.3	2.9
RDP80	9.0	8.3	4.7	6.4	1.5
SINTAX50	12.2	10.6	6.7	6.6	0.4
SINTAX80	5.9	5.4	3.6	1.3	0.0
SPINGO	10.4	9.1	5.0	3.5	0.0
TOP	27.9	14.7	8.3	11.4	0.4

Fig. S4. Misclassification rate results for the SP RDP ITS datasets at genus and species level. The misclassification rates for HiTaC, HiTaC_Filter, BTOP, BLCA, SPINGO, SINTAX80, Metaxa2 and KNN were 0% for the dataset SP RDP ITS 100. HiTaC's misclassification rate can be deemed adequate for the datasets with 90–99% identity, while misclassification rate reduced even further when the filter was applied to HiTaC. NBC50 and NBC80 crashed with memory limit exceeded for dataset SP RDP ITS 100, so their results are missing.

Table S1. List of software available in the TAXXI benchmark (1), which were used for comparison with HiTaC.

Method	Software	Algorithm reference
BLCA	BLCA v2.3-alpha	(9)
BTOP	blastn v2.10.1+	(10)
CT1	usearch v11.0.667	Re-implements Q1 (1)
CT2	usearch v11.0.667	Re-implements Q2_VS (1)
HiTaC	HiTaC v2.2.2	(This paper)
KNN	mothur v1.48.0	(11)
KTOP	usearch v11.0.667	(1)
Metaxa2	Metaxa 2.2.3	(12)
Microclass	microclass v1.2	(13)
NBC	usearch v11.0.667	Re-implements RDP (1)
Q1	QIIME v1.9	(14)
Q2_BLAST	QIIME v2.2022.2	(15)
Q2_SK	QIIME v2.2022.2	(15)
Q2_VS	QIIME v2.2022.2	(15)
RDP	Classifier v2.13	(16)
SINTAX	usearch v11.0.667	(17)
SPINGO	SPINGO v1.3	(18)
TOP	usearch v11.0.667	(1)

Table S2. List of training datasets available in the TAXXI benchmark (1), which were selected for possessing taxonomic annotations of fungal ITS sequences up to species level and were used to compare HiTaC with similar software.

Name	Lowest Rank	Identity (%)	# Training sequences	Shortest sequence (bp)	Longest sequence (bp)	Median (bp)	Average (bp)
SP RDP ITS 90	Species	90	9336	235	1373	539	552.02
SP RDP ITS 95	Species	95	11439	280	1373	540	551.43
SP RDP ITS 97	Species	97	12545	251	1373	537	549.23
SP RDP ITS 99	Species	99	10901	235	1373	531	546.37
SP RDP ITS 100	Species	100	16055	235	1373	530	545.09

Table S3. List of test datasets available in the TAXXI benchmark (1), which were selected for possessing taxonomic annotations of fungal ITS sequences up to species level and were used to compare HiTaC with similar software.

Name	Lowest Rank	Identity (%)	# Test sequences	Shortest sequence (bp)	Longest sequence (bp)	Median (bp)	Average (bp)
SP RDP ITS 90	Species	90	1935	268	1216	521	535.75
SP RDP ITS 95	Species	95	1265	251	1155	520	538.00
SP RDP ITS 97	Species	97	1420	284	1211	518	533.24
SP RDP ITS 99	Species	99	3945	261	1336	528	542.52
SP RDP ITS 100	Species	100	16055	235	1373	530	545.09

Table S4. Commands and parameters to run BLCA.

```
python scripts/fasta_utax2_to_blca.py \
    {input.train} \
    {output.reference_reads} \
    {output.reference_taxonomy}

python scripts/fasta_utax2_to_blca.py \
    {input.test} \
    {output.query_reads} \
    {output.query_taxonomy}

makeblastdb \
    -in {input.reference_reads} \
    -dbtype nucl \
    -parse_seqids \
    -out {params.database}

2.blca_main.py \
    -i {input.query_reads} \
    -r {input.reference_taxonomy} \
    -q {params.database} \
    --proc {threads}

python scripts/blca2tab.py \
    {input.predictions} \
    {input.test} \
    > {output.predictions}
```

Table S5. Commands and parameters to run BTOP.

```
sed \
    "-es/;.*//" \
    < {input.train} \
    > {output.reference}

makeblastdb \
    -in {input.reference} \
    -dbtype nucl \
    -parse_seqids \
    -out {params.database}

blastn \
    -task megablast \
    -db {params.database} \
    -query {input.test} \
    -num_threads {threads} \
    -max_target_seqs 1 \
    -outfmt "6 qseqid sseqid" \
    -evalue 0.01 \
    > {output.predictions}

python scripts/btop2tab.py \
    {input.predictions} \
    {input.train} \
    {output.predictions}
```

Table S6. Commands and parameters to run CT1.

```
usearch \
    -db {input.train} \
    -cons_tax {input.test} \
    -strand plus \
    -tabbedout \
    {output.predictions} \
    -strand plus \
    -id 0.7 \
    -maxaccepts 3 \
    -maxrejects 8 \
    -maj 0.51
```

Table S7. Commands and parameters to run CT2.

```
usearch \
    -db {input.train} \
    -cons_tax {input.test} \
    -strand plus \
    -tabbedout \
    {output.predictions} \
    -strand plus \
    -id 0.7 \
    -maxaccepts 10 \
    -maxrejects 32 \
    -maj 0.51
```

Table S8. Commands and parameters to run HiTaC.

```
qiime hitac fit \
    --i-reference-reads {input.reference_reads} \
    --i-reference-taxonomy {input.reference_taxonomy} \
    --p-kmer 6 \
    --p-threads {threads} \
    --o-classifier {output.classifier}

qiime hitac classify \
    --i-reads {input.query_reads} \
    --i-classifier {output.classifier} \
    --p-kmer 6 \
    --p-threads {threads} \
    --o-classification {output.predictions}
```

Table S9. Commands and parameters to run HiTaC_Filter.

```
qiime hitac fit-filter \
    --i-reference-reads {input.reference_reads} \
    --i-reference-taxonomy {input.reference_taxonomy} \
    --p-kmer 6 \
    --p-threads {threads} \
    --o-filter {output.filter}

qiime hitac filter \
    --i-filter {output.filter} \
    --i-reads {input.query_reads} \
    --i-classification {input.unfiltered_predictions} \
    --p-threshold 0.7 \
    --p-kmer 6 \
    --p-threads {threads} \
    --o-filtered-classification {output.filtered_predictions}
```

Table S10. Commands and parameters to run KNN.

```
cp {input.test} {output.query_reads}

python2 scripts/mothur_make_taxtrainfiles.py \
    {input.train} \
    {output.reference_reads} \
    {output.reference_taxonomy}

mothur \
    "#classify.seqs(fasta={input.query_reads}, \
    template={input.reference_reads}, \
    taxonomy={input.reference_taxonomy}, \
    method=knn, processors={threads})"

python scripts/motknn2utax2.py \
    {input.predictions} \
    > {output.predictions}
```

Table S11. Commands and parameters to run KTOP.

```
usearch \
    -db {input.train} \
    -sintax {input.test} \
    -strand plus \
    -tabbedout \
    {output.predictions} \
    -strand plus \
    -ktop
```

Table S12. Commands and parameters to run Metaxa2.

```
python scripts/fasta_utax2_to_metaxa2.py \
    {input.train} \
    {output.reference_reads} \
    {output.reference_taxonomy}

metaxa2_dbb \
    -b {input.reference_reads} \
    -o {params.database} \
    -t {input.reference_taxonomy} \
    --auto_rep T \
    --cpu {threads} \
    --mode divergent

metaxa2 \
    -i {input.test} \
    -d {params.blast} \
    -p {params.hhms} \
    -o {params.predictions} \
    -cpu {threads}

python scripts/metaxa2tab.py \
    {input.predictions} \
    {input.test} \
    > {output.predictions}
```

Table S13. Commands and parameters to run Microclass.

```
Rscript scripts/microclass.R \
    {input.train} \
    {input.test} \
    {output.predictions}

python scripts/microclass2tab.py \
    {input.predictions} \
    {output.predictions}
```

Table S14. Commands and parameters to run NBC.

```
usearch \
    -nbc_tax \
    {input.test} \
    -db {input.train} \
    -strand plus \
    -tabbedout {output.predictions}

python scripts/bbc_cutoff.py \
    {input.predictions} \
    0.5 \
    > {output.predictions}

python scripts/bbc_cutoff.py \
    {input.predictions} \
    0.8 \
    > {output.predictions}
```

Table S15. Commands and parameters to run Q1.

```
python scripts/fasta_utax2qiime.py \
    {input.train} \
    {output.reference_reads} \
    {output.reference_taxonomy}

assign_taxonomy.py \
    -i {input.test} \
    -m uclust \
    -r {input.reference_reads} \
    -t {input.reference_taxonomy} \
    -o {params.tmpdir}

python scripts/qiimetax2tab.py \
    {input.predictions} \
    > {output.predictions}
```

Table S16. Commands and parameters to run Q2_BLAST.

```
qiime feature-classifier classify-consensus-blast \
    --i-query {input.query_reads} \
    --i-reference-reads {input.reference_reads} \
    --i-reference-taxonomy {input.reference_taxonomy} \
    --o-classification {output.predictions} \
    --o-search-results {output.search_results}
```

Table S17. Commands and parameters to run Q2_SK.

```
qiime feature-classifier fit-classifier-naive-bayes \
    --i-reference-reads {input.reference_reads} \
    --i-reference-taxonomy {input.reference_taxonomy} \
    --o-classifier {output.classifier}

qiime feature-classifier classify-sklearn \
    --i-classifier {output.classifier} \
    --i-reads {input.query_reads} \
    --p-n-jobs {threads} \
    --o-classification {output.predictions}
```

Table S18. Commands and parameters to run Q2_VS.

```
qiime feature-classifier classify-consensus-vsearch \
    --i-query {input.query_reads} \
    --p-threads {threads} \
    --i-reference-reads {input.reference_reads} \
    --i-reference-taxonomy {input.reference_taxonomy} \
    --o-classification {output.predictions} \
    --o-search-results {output.search_results}
```

Table S19. Commands and parameters to run RDP.

```
python scripts/fasta_utax2rdp.py \
    {input.train} \
    {output.reference_taxonomy} \
    > {output.reference_reads}

workdir=`echo /usr/bin/rdp_classifier_2.13/dist/classifier.jar | sed "-es/e:/\/e/"`

rd=`dirname $workdir | sed "-es/\/dist//"`

props=$(find $rd | grep rRNAClassifier.properties | head -1)

cp $props {output.properties}

java \
    -Xmx8g \
    -cp /usr/bin/rdp_classifier_2.13/dist/classifier.jar \
    edu/msu/cme/rdp/classifier/train/ClassifierTraineeMaker \
    train \
    -t {input.reference_taxonomy} \
    -s {input.reference_reads} \
    -o {params.tmpdir}
java \
    -Xmx1g \
    -jar /usr/bin/rdp_classifier_2.13/dist/classifier.jar \
    -t {output.properties} \
    -q {input.test} \
    -o {output.predictions}

python scripts/rdpc2tab3.py \
    {input.predictions} \
    50 \
    > {output.predictions}

python scripts/rdpc2tab3.py \
    {input.predictions} \
    80 \
    > {output.predictions}
```

Table S20. Commands and parameters to run SINTAX.

```
usearch \
    -sintax \
    {input.test} \
    -db {input.train} \
    -strand plus \
    -tabbedout {output.predictions}

python scripts/bbc_cutoff.py \
    {input.predictions} \
    0.5 \
    > {output.predictions}

python scripts/bbc_cutoff.py \
    {input.predictions} \
    0.8 \
    > {output.predictions}
```

Table S21. Commands and parameters to run SPINGO.

```
python scripts/fasta_utax2spingo.py \
    {input.train} \
    > {output.reference_reads}

spingo \
    -i {input.test} \
    -d {input.reference_reads} \
    -p {threads} \
    > {output.predictions}

python scripts/spingo2tab.py \
    {input.predictions} \
    > {output.predictions}
```

Table S22. Commands and parameters to run TOP.

```
usearch \
    -db {input.train} \
    -cons_tax {input.test} \
    -strand plus \
    -tabbedout {output.predictions} \
    -strand plus \
    -id 0.7 \
    -maxaccepts 3 \
    -maxrejects 16 \
    -top_hit_only
```

Table S23. Resources benchmark computed for the dataset SP RDP ITS 90.

Method	Training Time (hh:mm:ss)	Training Memory (MB)	Classification Time (hh:mm:ss)	Classification Memory (MB)
BTOP	00:00:00	28.28	00:00:17	21.16
BLCA	00:00:00	22.51	00:22:22	27.44
RDP	00:00:20	18.15	00:00:17	26.23
Q2_SK	00:00:37	26.05	00:00:39	25.40
HiTaC	00:03:18	22.84	00:00:03	18.24
HiTaC_Filter	00:51:39	22.94	00:00:05	23.63
Metaxa2	01:40:34	17.42	00:01:18	21.11
TOP	-	-	00:00:00	20.84
CT1	-	-	00:00:00	21.75
KTOP	-	-	00:00:00	21.83
CT2	-	-	00:00:00	21.86
KNN	-	-	00:00:01	24.03
SINTAX	-	-	00:00:01	24.64
Q1	-	-	00:00:03	21.52
SPINGO	-	-	00:00:04	24.29
Microclass	-	-	00:00:14	26.50
NBC	-	-	00:00:15	14.87
Q2_BLAST	-	-	00:02:21	20.94
Q2_VS	-	-	00:02:23	25.89

Table S24. Resources benchmark computed for the dataset SP RDP ITS 95.

Method	Training Time (hh:mm:ss)	Training Memory (MB)	Classification Time (hh:mm:ss)	Classification Memory (MB)
BTOP	00:00:00	17.47	00:00:14	23.90
BLCA	00:00:00	15.71	00:14:10	19.42
RDP	00:00:30	20.00	00:00:14	24.22
Q2_SK	00:00:49	24.25	00:00:43	25.95
HiTaC	00:04:00	20.32	00:00:03	19.25
HiTaC_Filter	01:18:19	21.29	00:00:04	25.39
Metaxa2	02:44:28	19.03	00:01:03	18.50
TOP	-	-	00:00:00	16.29
CT2	-	-	00:00:00	18.63
CT1	-	-	00:00:00	19.71
KTOP	-	-	00:00:00	20.18
SINTAX	-	-	00:00:01	15.03
KNN	-	-	00:00:01	17.00
Q1	-	-	00:00:03	27.53
SPINGO	-	-	00:00:05	24.41
NBC	-	-	00:00:16	21.65
Microclass	-	-	00:00:17	23.75
Q2_VS	-	-	00:01:42	27.21
Q2_BLAST	-	-	00:01:53	23.95

Table S25. Resources benchmark computed for the dataset SP RDP ITS 97.

Method	Training Time (hh:mm:ss)	Training Memory (MB)	Classification Time (hh:mm:ss)	Classification Memory (MB)
BTOP	00:00:00	23.84	00:00:17	21.43
BLCA	00:00:00	13.42	00:14:08	19.36
RDP	00:00:35	23.73	00:00:17	24.48
Q2_SK	00:00:55	22.33	00:00:48	27.01
HiTaC	00:04:28	14.47	00:00:03	20.16
HiTaC_Filter	01:35:15	19.27	00:00:05	22.26
Metaxa2	02:42:24	12.98	00:01:17	14.35
CT2	-	-	00:00:00	20.02
CT1	-	-	00:00:00	22.20
TOP	-	-	00:00:00	22.48
KTOP	-	-	00:00:00	28.46
SINTAX	-	-	00:00:01	22.36
KNN	-	-	00:00:01	24.37
Q1	-	-	00:00:03	17.74
SPINGO	-	-	00:00:06	28.26
NBC	-	-	00:00:18	19.19
Microclass	-	-	00:00:19	20.40
Q2_VS	-	-	00:01:52	15.38
Q2_BLAST	-	-	00:02:18	23.07

Table S26. Resources benchmark computed for the dataset SP RDP ITS 99.

Method	Training Time (hh:mm:ss)	Training Memory (MB)	Classification Time (hh:mm:ss)	Classification Memory (MB)
BTOP	00:00:00	15.54	00:00:42	27.00
BLCA	00:00:00	25.02	00:27:30	22.30
RDP	00:00:43	22.35	00:00:51	25.66
Q2_SK	00:00:55	23.56	00:01:11	24.18
HiTaC	00:04:51	21.34	00:00:07	21.29
HiTaC_Filter	01:32:13	18.90	00:00:11	25.14
Metaxa2	01:57:22	20.55	00:03:08	19.63
CT2	-	-	00:00:01	19.60
CT1	-	-	00:00:01	20.47
TOP	-	-	00:00:01	20.86
KTOP	-	-	00:00:01	22.03
KNN	-	-	00:00:02	22.84
SINTAX	-	-	00:00:03	23.03
Q1	-	-	00:00:05	14.33
SPINGO	-	-	00:00:06	19.82
Microclass	-	-	00:00:23	21.20
NBC	-	-	00:00:33	22.34
Q2_BLAST	-	-	00:05:23	24.81
Q2_VS	-	-	00:08:10	29.31

Table S27. Resources benchmark computed for the dataset SP RDP ITS 100.

Method	Training Time (hh:mm:ss)	Training Memory (MB)	Classification Time (hh:mm:ss)	Classification Memory (MB)
BTOP	00:00:00	27.71	00:04:01	20.39
BLCA	00:00:00	19.81	00:04:30	21.77
RDP	00:00:50	24.66	00:03:42	20.38
Q2_SK	00:01:17	28.97	00:02:52	27.91
HiTaC	00:05:16	26.81	00:00:26	25.20
HiTaC_Filter	02:32:27	20.39	00:00:45	20.36
Metaxa2	02:43:29	12.67	00:18:10	13.79
KTOP	-	-	00:00:03	18.77
CT1	-	-	00:00:03	22.85
TOP	-	-	00:00:03	25.27
CT2	-	-	00:00:04	18.37
KNN	-	-	00:00:05	23.22
Q1	-	-	00:00:11	19.79
SPINGO	-	-	00:00:16	24.14
SINTAX	-	-	00:00:17	20.38
Microclass	-	-	00:00:38	17.61
Q2_BLAST	-	-	00:30:37	21.40
Q2_VS	-	-	00:41:07	25.41
NBC	-	Exceeded	-	Exceeded

Table S28. Hierarchical metrics computed for the dataset SP RDP ITS 90.

Method	F1-score	Precision	Recall
HiTaC_Filter	86.61	96.05	78.86
Q2_VS	84.92	93.42	77.84
CT2	84.92	94.10	77.37
SINTAX50	84.80	92.59	78.22
RDP80	84.80	93.83	77.36
NBC80	84.79	93.77	77.37
SINTAX80	84.76	96.63	75.48
Q2_BLAST	84.71	93.57	77.38
KNN	83.08	98.47	71.84
NBC50	82.92	87.43	78.85
Q2_SK	82.90	87.68	78.62
RDP50	82.79	87.16	78.83
Metaxa2	82.46	87.81	77.73
CT1	80.76	82.73	78.89
HiTaC	80.38	80.38	80.38
BTOP	79.33	79.33	79.33
Microclass	79.24	79.24	79.24
TOP	79.16	79.16	79.16
KTOP	79.05	79.05	79.05
BLCA	78.52	84.95	72.99
Q1	77.80	82.43	73.66
SPINGO	32.49	75.92	20.66

Table S29. Hierarchical metrics computed for the dataset SP RDP ITS 95.

Method	F1-score	Precision	Recall
CT2	88.13	96.94	80.79
Q2_VS	88.08	96.45	81.04
HiTaC_Filter	88.05	94.66	82.30
Q2_BLAST	87.96	96.28	80.97
SINTAX80	87.40	95.32	80.70
RDP80	86.58	92.09	81.69
NBC80	86.54	92.20	81.54
SINTAX50	86.26	90.72	82.22
Metaxa2	85.67	90.34	81.46
KNN	85.41	99.25	74.95
Q2_SK	85.09	88.16	82.22
RDP50	84.56	86.77	82.45
NBC50	84.51	86.71	82.42
Q1	84.09	86.03	82.24
CT1	83.88	85.67	82.17
HiTaC	82.96	82.96	82.96
TOP	82.64	82.64	82.64
Microclass	82.59	82.59	82.59
BTOP	82.55	82.55	82.55
BLCA	82.54	86.90	78.60
KTOP	82.50	82.50	82.50
SPINGO	37.47	76.18	24.84

Table S30. Hierarchical metrics computed for the dataset SP RDP ITS 97.

Method	F1-score	Precision	Recall
Q2_BLAST	88.96	96.94	82.19
Q2_VS	88.95	96.99	82.14
SINTAX80	88.84	95.36	83.16
CT2	88.75	97.15	81.68
HiTaC_Filter	88.65	93.71	84.10
RDP80	87.93	92.28	83.96
NBC80	87.89	92.19	83.97
SINTAX50	87.45	90.79	84.34
Metaxa2	87.18	91.63	83.14
Q2_SK	86.80	89.31	84.43
RDP50	86.05	87.52	84.63
NBC50	85.96	87.33	84.64
KNN	85.69	99.32	75.35
CT1	85.55	87.44	83.74
Q1	85.49	87.31	83.75
HiTaC	84.78	84.78	84.78
TOP	84.75	84.75	84.75
Microclass	84.73	84.73	84.73
KTOP	84.72	84.72	84.72
BTOP	84.72	84.72	84.72
BLCA	83.72	88.46	79.47
SPINGO	40.14	79.08	26.89

Table S31. Hierarchical metrics computed for the dataset SP RDP ITS 99.

Method	F1-score	Precision	Recall
HiTaC_Filter	96.41	98.30	94.59
SINTAX50	96.28	97.25	95.33
SINTAX80	96.14	98.77	93.65
HiTaC	96.06	96.06	96.06
RDP80	96.03	97.45	94.65
Q2_SK	96.02	96.92	95.14
NBC80	96.00	97.37	94.67
BTOP	95.99	95.99	95.99
TOP	95.96	95.96	95.96
Microclass	95.96	95.96	95.96
KTOP	95.84	95.84	95.84
RDP50	95.66	95.97	95.35
NBC50	95.66	95.92	95.39
BLCA	93.33	96.37	90.47
Q1	92.36	94.84	90.01
Metaxa2	92.13	98.32	86.67
CT1	90.60	93.29	88.06
Q2_VS	90.02	97.90	83.32
Q2_BLAST	89.69	97.78	82.84
CT2	89.02	97.77	81.71
KNN	85.20	99.99	74.23
SPINGO	54.08	95.03	37.80

Table S32. Hierarchical metrics computed for the dataset SP RDP ITS 100.

Method	F1-score	Precision	Recall
HiTaC	100.00	100.00	100.00
BTOP	100.00	100.00	100.00
TOP	99.94	99.94	99.94
KTOP	99.92	99.92	99.92
Microclass	99.91	99.91	99.91
HiTaC_Filter	99.84	100.00	99.68
SINTAX50	99.79	99.94	99.64
Q2_SK	99.60	99.88	99.33
RDP50	99.52	99.58	99.45
RDP80	99.38	99.78	98.99
SINTAX80	98.98	100.00	97.99
Metaxa2	98.14	100.00	96.35
Q1	98.10	98.72	97.50
CT1	97.77	98.41	97.14
Q2_VS	90.77	98.27	84.33
Q2_BLAST	90.51	98.17	83.96
CT2	90.34	98.18	83.65
KNN	86.62	100.00	76.40
SPINGO	59.54	100.00	42.39

Table S33. TAXXI metrics computed for the dataset SP RDP ITS 90 at the phylum level.

Method	Accuracy	Misclassification Rate	Over-classification Rate	True Positive Rate	Under-classification Rate
BTOP	100.00	0.00	.	100.00	0.00
CT1	100.00	0.00	.	100.00	0.00
CT2	100.00	0.00	.	100.00	0.00
HiTaC	100.00	0.00	.	100.00	0.00
HiTaC_Filter	100.00	0.00	.	100.00	0.00
KTOP	100.00	0.00	.	100.00	0.00
Metaxa2	100.00	0.00	.	100.00	0.00
Microclass	100.00	0.00	.	100.00	0.00
NBC50	100.00	0.00	.	100.00	0.00
NBC80	100.00	0.00	.	100.00	0.00
Q2_SK	100.00	0.00	.	100.00	0.00
RDP50	100.00	0.00	.	100.00	0.00
RDP80	100.00	0.00	.	100.00	0.00
SINTAX50	100.00	0.00	.	100.00	0.00
SINTAX80	100.00	0.00	.	100.00	0.00
TOP	100.00	0.00	.	100.00	0.00
KNN	99.90	0.00	.	99.90	0.10
Q2_VS	99.80	0.00	.	99.80	0.20
Q2_BLAST	99.70	0.00	.	99.70	0.30
Q1	92.80	0.00	.	92.80	7.20
BLCA	92.50	0.00	.	92.50	7.50
SPINGO	0.00	0.00	.	0.00	100.00

Table S34. TAXXI metrics computed for the dataset SP RDP ITS 90 at the class level.

Method	Accuracy	Misclassification Rate	Over-classification Rate	True Positive Rate	Under-classification Rate
CT2	99.90	0.10	.	99.90	0.00
HiTaC	99.90	0.10	.	99.90	0.00
HiTaC_Filter	99.90	0.10	.	99.90	0.00
Q2_VS	99.80	0.10	.	99.80	0.20
BTOP	99.80	0.20	.	99.80	0.00
Microclass	99.80	0.20	.	99.80	0.00
Q2_SK	99.80	0.20	.	99.80	0.00
Q2_BLAST	99.70	0.10	.	99.70	0.30
Metaxa2	99.70	0.30	.	99.70	0.00
TOP	99.70	0.30	.	99.70	0.00
RDP50	99.60	0.40	.	99.60	0.00
NBC80	99.50	0.10	.	99.50	0.40
RDP80	99.50	0.10	.	99.50	0.40
SINTAX80	99.50	0.10	.	99.50	0.40
SINTAX50	99.50	0.20	.	99.50	0.30
CT1	99.50	0.40	.	99.50	0.10
NBC50	99.50	0.40	.	99.50	0.10
KTOP	99.50	0.50	.	99.50	0.00
KNN	98.60	0.10	.	98.60	1.30
Q1	92.60	0.20	.	92.60	7.20
BLCA	92.40	0.20	.	92.40	7.50
SPINGO	0.00	0.00	.	0.00	100.00

Table S35. TAXXI metrics computed for the dataset SP RDP ITS 90 at the order level.

Method	Accuracy	Misclassification Rate	Over-classification Rate	True Positive Rate	Under-classification Rate
HiTaC_Filter	98.20	1.60	.	98.20	0.20
HiTaC	98.20	1.80	.	98.20	0.00
Microclass	98.20	1.80	.	98.20	0.00
Q2_VS	98.10	1.50	.	98.10	0.40
Q2_SK	98.10	1.60	.	98.10	0.30
BTOP	98.10	1.90	.	98.10	0.00
TOP	98.10	1.90	.	98.10	0.00
Q2_BLAST	98.00	1.60	.	98.00	0.50
CT2	97.90	1.80	.	97.90	0.40
NBC50	97.90	1.90	.	97.90	0.30
RDP50	97.90	2.00	.	97.90	0.10
KTOP	97.90	2.10	.	97.90	0.00
CT1	97.90	2.10	.	97.90	0.10
RDP80	97.80	1.30	.	97.80	0.80
NBC80	97.80	1.30	.	97.80	0.90
SINTAX50	97.80	1.50	.	97.80	0.70
Metaxa2	97.20	2.40	.	97.20	0.40
SINTAX80	97.10	1.30	.	97.10	1.60
KNN	93.20	0.90	.	93.20	5.80
Q1	91.40	1.40	.	91.40	7.20
BLCA	90.40	1.80	.	90.40	7.80
SPINGO	0.00	0.00	.	0.00	100.00

Table S36. TAXXI metrics computed for the dataset SP RDP ITS 90 at the family level.

Method	Accuracy	Misclassification Rate	Over-classification Rate	True Positive Rate	Under-classification Rate
HiTaC	95.10	4.90	.	95.10	0.00
HiTaC_Filter	94.00	3.40	.	94.00	2.60
TOP	93.90	6.00	.	94.00	0.00
Microclass	93.90	6.10	.	93.90	0.00
BTOP	93.70	6.20	.	93.80	0.00
KTOP	93.40	6.50	.	93.50	0.00
CT1	93.30	6.60	.	93.40	0.10
NBC50	93.20	5.40	.	93.20	1.40
RDP50	93.20	5.90	.	93.30	0.80
Q2_SK	93.10	4.60	.	93.20	2.30
SINTAX50	92.70	4.30	.	92.70	3.00
Q2_VS	91.70	5.40	.	91.80	2.80
NBC80	91.60	3.30	.	91.70	5.00
RDP80	91.50	3.50	.	91.60	5.00
Q2_BLAST	91.40	5.60	.	91.40	2.90
SPINGO	91.30	3.60	.	91.30	5.10
CT2	91.30	5.70	.	91.30	3.00
Metaxa2	90.50	6.40	.	90.50	3.10
SINTAX80	87.60	2.30	.	87.70	10.00
Q1	87.30	5.20	.	87.30	7.40
BLCA	86.90	4.40	.	87.00	8.60
KNN	80.20	1.40	.	80.20	18.40

Table S37. TAXXI metrics computed for the dataset SP RDP ITS 90 at the genus level.

Method	Accuracy	Misclassification Rate	Over-classification Rate	True Positive Rate	Under-classification Rate
HiTaC	69.50	19.70	100.00	80.30	0.00
HiTaC_Filter	64.80	10.30	44.10	69.30	20.40
NBC50	64.20	17.60	67.00	70.90	11.50
RDP50	63.90	18.60	67.80	70.60	10.80
BTOP	63.70	26.60	100.00	73.40	0.00
Microclass	62.70	27.50	100.00	72.50	0.00
Q2_SK	62.40	18.80	62.50	68.50	12.70
KTOP	62.40	27.80	100.00	72.20	0.00
TOP	62.40	27.90	100.00	72.10	0.00
CT1	62.10	27.90	92.70	71.00	1.10
SINTAX50	61.90	12.20	47.10	66.50	21.30
Q1	60.30	23.00	81.20	68.00	9.00
Metaxa2	59.00	19.70	71.60	65.60	14.70
Q2_VS	58.50	19.50	61.70	64.20	16.40
BLCA	58.40	15.90	72.40	65.00	19.10
SPINGO	57.90	10.40	41.80	61.70	27.90
RDP80	57.70	9.00	34.90	60.80	30.20
NBC80	57.60	8.80	36.80	60.90	30.30
Q2_BLAST	56.00	19.40	61.70	61.40	19.20
CT2	55.20	19.00	64.00	60.80	20.30
SINTAX80	49.20	5.90	22.20	51.00	43.10
KNN	34.80	3.30	18.40	35.80	60.90

Table S38. TAXXI metrics computed for the dataset SP RDP ITS 90 at the species level.

Method	Accuracy	Misclassification Rate	Over-classification Rate	True Positive Rate	Under-classification Rate
CT2	0.00	.	1.30	.	.
HiTaC_Filter	0.00	.	2.70	.	.
Q2_BLAST	0.00	.	4.80	.	.
Q2_VS	0.00	.	6.20	.	.
SINTAX80	0.00	.	6.60	.	.
RDP80	0.00	.	18.20	.	.
NBC80	0.00	.	18.60	.	.
SINTAX50	0.00	.	20.80	.	.
SPINGO	0.00	.	22.50	.	.
Metaxa2	0.00	.	39.70	.	.
Q2_SK	0.00	.	46.20	.	.
NBC50	0.00	.	47.50	.	.
RDP50	0.00	.	47.70	.	.
BLCA	0.00	.	53.10	.	.
Q1	0.00	.	64.90	.	.
CT1	0.00	.	69.50	.	.
BTOP	0.00	.	100.00	.	.
HiTaC	0.00	.	100.00	.	.
KTOP	0.00	.	100.00	.	.
Microclass	0.00	.	100.00	.	.
TOP	0.00	.	100.00	.	.
KNN	.	.	0.00	.	.

Table S39. TAXXI metrics computed for the dataset SP RDP ITS 95 at the phylum level.

Method	Accuracy	Misclassification Rate	Over-classification Rate	True Positive Rate	Under-classification Rate
BTOP	100.00	0.00	.	100.00	0.00
CT1	100.00	0.00	.	100.00	0.00
CT2	100.00	0.00	.	100.00	0.00
HiTaC	100.00	0.00	.	100.00	0.00
HiTaC_Filter	100.00	0.00	.	100.00	0.00
KNN	100.00	0.00	.	100.00	0.00
KTOP	100.00	0.00	.	100.00	0.00
Metaxa2	100.00	0.00	.	100.00	0.00
Microclass	100.00	0.00	.	100.00	0.00
NBC50	100.00	0.00	.	100.00	0.00
NBC80	100.00	0.00	.	100.00	0.00
Q2_BLAST	100.00	0.00	.	100.00	0.00
Q2_SK	100.00	0.00	.	100.00	0.00
Q2_VS	100.00	0.00	.	100.00	0.00
RDP50	100.00	0.00	.	100.00	0.00
RDP80	100.00	0.00	.	100.00	0.00
SINTAX50	100.00	0.00	.	100.00	0.00
SINTAX80	100.00	0.00	.	100.00	0.00
TOP	100.00	0.00	.	100.00	0.00
Q1	99.80	0.00	.	99.80	0.20
BLCA	95.90	0.00	.	95.90	4.10
SPINGO	0.00	0.00	.	0.00	100.00

Table S40. TAXXI metrics computed for the dataset SP RDP ITS 95 at the class level.

Method	Accuracy	Misclassification Rate	Over-classification Rate	True Positive Rate	Under-classification Rate
CT2	99.70	0.30	.	99.70	0.00
HiTaC	99.70	0.30	.	99.70	0.00
HiTaC_Filter	99.70	0.30	.	99.70	0.00
Q2_BLAST	99.70	0.30	.	99.70	0.00
Q2_VS	99.70	0.30	.	99.70	0.00
CT1	99.60	0.40	.	99.60	0.00
KTOP	99.60	0.40	.	99.60	0.00
TOP	99.60	0.40	.	99.60	0.00
SINTAX50	99.50	0.40	.	99.50	0.10
BTOP	99.50	0.50	.	99.50	0.00
RDP50	99.50	0.50	.	99.50	0.00
NBC80	99.40	0.40	.	99.40	0.20
Q2_SK	99.40	0.40	.	99.40	0.20
RDP80	99.40	0.40	.	99.40	0.20
SINTAX80	99.40	0.40	.	99.40	0.20
NBC50	99.40	0.50	.	99.40	0.10
Metaxa2	99.40	0.50	.	99.40	0.20
Q1	99.40	0.50	.	99.40	0.20
Microclass	99.40	0.60	.	99.40	0.00
KNN	98.70	0.30	.	98.70	0.90
BLCA	95.40	0.50	.	95.40	4.10
SPINGO	0.00	0.00	.	0.00	100.00

Table S41. TAXXI metrics computed for the dataset SP RDP ITS 95 at the order level.

Method	Accuracy	Misclassification Rate	Over-classification Rate	True Positive Rate	Under-classification Rate
Q2_VS	99.10	0.60	.	99.10	0.20
CT2	99.10	0.70	.	99.10	0.20
Q2_BLAST	99.10	0.70	.	99.10	0.20
HiTaC_Filter	99.10	0.80	.	99.10	0.10
HiTaC	99.10	0.90	.	99.10	0.00
KTOP	99.10	0.90	.	99.10	0.00
TOP	99.10	0.90	.	99.10	0.00
SINTAX50	99.00	0.80	.	99.00	0.20
RDP50	99.00	1.00	.	99.00	0.00
RDP80	98.90	0.90	.	98.90	0.20
NBC50	98.90	1.00	.	98.90	0.10
BTOP	98.90	1.10	.	98.90	0.00
CT1	98.90	1.10	.	98.90	0.00
Microclass	98.90	1.10	.	98.90	0.00
Q2_SK	98.80	0.90	.	98.80	0.20
NBC80	98.70	0.80	.	98.70	0.60
Metaxa2	98.70	1.00	.	98.70	0.20
Q1	98.70	1.20	.	98.70	0.20
SINTAX80	98.40	0.60	.	98.40	1.00
KNN	94.80	0.50	.	94.80	4.70
BLCA	94.80	0.90	.	94.80	4.30
SPINGO	0.00	0.00	.	0.00	100.00

Table S42. TAXXI metrics computed for the dataset SP RDP ITS 95 at the family level.

Method	Accuracy	Misclassification Rate	Over-classification Rate	True Positive Rate	Under-classification Rate
HiTaC	98.00	1.90	.	98.10	0.00
BTOP	97.80	2.10	.	97.90	0.00
KTOP	97.80	2.10	.	97.90	0.00
TOP	97.80	2.10	.	97.90	0.00
HiTaC_Filter	97.60	1.00	.	97.60	1.30
RDP50	97.60	2.20	.	97.70	0.10
Microclass	97.50	2.40	.	97.60	0.00
SINTAX50	97.30	1.70	.	97.40	0.90
NBC50	97.30	2.20	.	97.40	0.40
Q2_SK	97.20	1.80	.	97.30	0.90
Q1	97.20	2.50	.	97.20	0.30
CT1	97.00	2.90	.	97.10	0.00
RDP80	96.80	1.40	.	96.90	1.70
SPINGO	96.80	1.50	.	96.90	1.60
NBC80	96.60	1.30	.	96.70	2.00
Metaxa2	96.40	2.10	.	96.40	1.60
SINTAX80	96.10	1.00	.	96.10	2.80
Q2_VS	95.80	2.20	.	95.80	2.00
CT2	95.70	2.30	.	95.70	2.00
Q2_BLAST	95.60	2.50	.	95.60	2.00
BLCA	93.00	1.90	.	93.00	5.10
KNN	85.20	0.90	.	85.20	13.80

Table S43. TAXXI metrics computed for the dataset SP RDP ITS 95 at the genus level.

Method	Accuracy	Misclassification Rate	Over-classification Rate	True Positive Rate	Under-classification Rate
HiTaC	83.60	12.90	100.00	87.10	0.00
Microclass	82.00	14.60	100.00	85.40	0.00
TOP	81.90	14.70	100.00	85.30	0.00
NBC50	81.80	13.10	78.40	84.50	2.40
RDP50	81.40	12.90	82.40	84.30	2.80
BTOP	81.30	15.20	100.00	84.80	0.00
Q1	81.00	14.70	90.20	84.10	1.20
KTOP	80.80	15.80	100.00	84.20	0.00
HiTaC_Filter	80.60	8.30	70.60	82.90	8.70
Q2_SK	80.60	11.80	80.40	83.30	4.90
SINTAX50	80.50	10.60	72.50	82.90	6.40
CT1	79.80	16.10	96.10	83.00	0.90
SPINGO	78.10	9.10	64.70	80.20	10.60
RDP80	77.60	8.30	66.70	79.80	11.90
NBC80	77.20	8.20	62.70	79.20	12.60
Metaxa2	76.60	10.60	74.50	79.00	10.40
BLCA	75.90	10.20	76.50	78.30	11.40
Q2_VS	73.30	11.00	76.50	75.70	13.30
Q2_BLAST	73.10	12.40	76.50	75.50	12.10
SINTAX80	72.40	5.40	49.00	73.90	20.80
CT2	72.00	10.60	70.60	74.10	15.20
KNN	47.70	1.90	9.80	47.90	50.20

Table S44. TAXXI metrics computed for the dataset SP RDP ITS 95 at the species level.

Method	Accuracy	Misclassification Rate	Over-classification Rate	True Positive Rate	Under-classification Rate
Q2_BLAST	2.00	.	3.40	.	.
Q2_VS	1.80	.	4.10	.	.
HiTaC_Filter	0.80	.	19.50	.	.
SINTAX80	0.40	.	18.60	.	.
SINTAX50	0.40	.	42.80	.	.
Q2_SK	0.30	.	59.60	.	.
BTOP	0.30	.	100.00	.	.
NBC80	0.20	.	35.30	.	.
RDP80	0.20	.	35.60	.	.
SPINGO	0.20	.	39.90	.	.
RDP50	0.20	.	68.50	.	.
NBC50	0.20	.	69.00	.	.
HiTaC	0.20	.	100.00	.	.
KTOP	0.20	.	100.00	.	.
Microclass	0.20	.	100.00	.	.
TOP	0.20	.	100.00	.	.
BLCA	0.10	.	62.60	.	.
Q1	0.10	.	71.40	.	.
CT2	0.00	.	1.50	.	.
Metaxa2	0.00	.	44.20	.	.
CT1	0.00	.	72.50	.	.
KNN	.	.	0.00	.	.

Table S45. TAXXI metrics computed for the dataset SP RDP ITS 97 at the phylum level.

Method	Accuracy	Misclassification Rate	Over-classification Rate	True Positive Rate	Under-classification Rate
BTOP	100.00	0.00	.	100.00	0.00
CT1	100.00	0.00	.	100.00	0.00
CT2	100.00	0.00	.	100.00	0.00
HiTaC	100.00	0.00	.	100.00	0.00
HiTaC_Filter	100.00	0.00	.	100.00	0.00
KTOP	100.00	0.00	.	100.00	0.00
Microclass	100.00	0.00	.	100.00	0.00
NBC50	100.00	0.00	.	100.00	0.00
NBC80	100.00	0.00	.	100.00	0.00
Q2_BLAST	100.00	0.00	.	100.00	0.00
Q2_SK	100.00	0.00	.	100.00	0.00
RDP50	100.00	0.00	.	100.00	0.00
RDP80	100.00	0.00	.	100.00	0.00
SINTAX50	100.00	0.00	.	100.00	0.00
SINTAX80	100.00	0.00	.	100.00	0.00
TOP	100.00	0.00	.	100.00	0.00
KNN	99.90	0.00	.	99.90	0.10
Metaxa2	99.90	0.00	.	99.90	0.10
Q1	99.90	0.00	.	99.90	0.10
Q2_VS	99.90	0.00	.	99.90	0.10
BLCA	94.50	0.00	.	94.50	5.50
SPINGO	0.00	0.00	.	0.00	100.00

Table S46. TAXXI metrics computed for the dataset SP RDP ITS 97 at the class level.

Method	Accuracy	Misclassification Rate	Over-classification Rate	True Positive Rate	Under-classification Rate
BTOP	99.90	0.10	.	99.90	0.00
CT2	99.90	0.10	.	99.90	0.00
HiTaC	99.90	0.10	.	99.90	0.00
HiTaC_Filter	99.90	0.10	.	99.90	0.00
KTOP	99.90	0.10	.	99.90	0.00
Microclass	99.90	0.10	.	99.90	0.00
NBC50	99.90	0.10	.	99.90	0.00
NBC80	99.90	0.10	.	99.90	0.00
Q2_BLAST	99.90	0.10	.	99.90	0.00
Q2_SK	99.90	0.10	.	99.90	0.00
RDP50	99.90	0.10	.	99.90	0.00
RDP80	99.90	0.10	.	99.90	0.00
SINTAX50	99.90	0.10	.	99.90	0.00
TOP	99.90	0.10	.	99.90	0.00
CT1	99.90	0.10	.	99.90	0.10
Q2_VS	99.90	0.10	.	99.90	0.10
SINTAX80	99.90	0.10	.	99.90	0.10
Q1	99.80	0.10	.	99.80	0.10
Metaxa2	99.70	0.10	.	99.70	0.20
KNN	98.10	0.10	.	98.10	1.80
BLCA	94.40	0.10	.	94.40	5.50
SPINGO	0.00	0.00	.	0.00	100.00

Table S47. TAXXI metrics computed for the dataset SP RDP ITS 97 at the order level.

Method	Accuracy	Misclassification Rate	Over-classification Rate	True Positive Rate	Under-classification Rate
BTOP	99.40	0.60	.	99.40	0.00
HiTaC	99.40	0.60	.	99.40	0.00
KTOP	99.40	0.60	.	99.40	0.00
Microclass	99.40	0.60	.	99.40	0.00
NBC50	99.40	0.60	.	99.40	0.00
NBC80	99.40	0.60	.	99.40	0.00
Q2_SK	99.40	0.60	.	99.40	0.00
RDP50	99.40	0.60	.	99.40	0.00
RDP80	99.40	0.60	.	99.40	0.00
TOP	99.40	0.60	.	99.40	0.00
HiTaC_Filter	99.40	0.60	.	99.40	0.10
SINTAX50	99.40	0.60	.	99.40	0.10
Q2_VS	99.30	0.50	.	99.30	0.20
SINTAX80	99.30	0.50	.	99.30	0.20
Q1	99.30	0.60	.	99.30	0.10
Q2_BLAST	99.30	0.60	.	99.30	0.10
CT2	99.20	0.60	.	99.20	0.10
CT1	99.20	0.70	.	99.20	0.10
Metaxa2	99.00	0.50	.	99.00	0.50
KNN	95.80	0.40	.	95.80	3.70
BLCA	93.90	0.60	.	93.90	5.60
SPINGO	0.00	0.00	.	0.00	100.00

Table S48. TAXXI metrics computed for the dataset SP RDP ITS 97 at the family level.

Method	Accuracy	Misclassification Rate	Over-classification Rate	True Positive Rate	Under-classification Rate
TOP	99.20	0.70	.	99.30	0.00
BTOP	99.20	0.80	.	99.20	0.00
KTOP	99.20	0.80	.	99.20	0.00
RDP80	99.10	0.70	.	99.20	0.10
Microclass	99.10	0.80	.	99.20	0.00
NBC50	99.10	0.80	.	99.20	0.00
Q2_SK	99.10	0.80	.	99.20	0.00
RDP50	99.10	0.80	.	99.20	0.00
NBC80	99.00	0.60	.	99.10	0.30
SINTAX50	98.90	0.70	.	99.00	0.30
HiTaC_Filter	98.90	0.80	.	98.90	0.30
HiTaC	98.90	1.00	.	99.00	0.00
SPINGO	98.70	0.70	.	98.80	0.50
CT1	98.70	1.10	.	98.70	0.10
Q1	98.50	1.30	.	98.50	0.20
SINTAX80	98.40	0.70	.	98.40	0.90
Metaxa2	98.20	0.70	.	98.20	1.10
Q2_BLAST	97.50	1.80	.	97.50	0.80
Q2_VS	97.50	1.90	.	97.50	0.60
CT2	96.50	2.30	.	96.50	1.20
BLCA	93.40	0.80	.	93.40	5.80
KNN	85.70	0.60	.	85.70	13.70

Table S49. TAXXI metrics computed for the dataset SP RDP ITS 97 at the genus level.

Method	Accuracy	Misclassification Rate	Over-classification Rate	True Positive Rate	Under-classification Rate
HiTaC	91.50	7.70	100.00	92.30	0.00
Microclass	91.10	8.20	100.00	91.80	0.00
KTOP	91.00	8.30	100.00	91.70	0.00
TOP	91.00	8.30	100.00	91.70	0.00
BTOP	90.90	8.40	100.00	91.60	0.00
NBC50	90.60	7.40	100.00	91.30	1.30
RDP50	90.50	7.10	100.00	91.20	1.70
SINTAX50	89.10	6.70	81.80	89.60	3.60
Q2_SK	89.10	6.70	100.00	89.80	3.50
CT1	87.90	10.60	90.90	88.60	0.90
HiTaC_Filter	87.80	5.00	81.80	88.40	6.60
Q1	87.30	11.40	81.80	87.90	0.70
SPINGO	86.60	5.00	81.80	87.20	7.90
RDP80	86.50	4.70	81.80	87.00	8.30
NBC80	86.50	4.90	81.80	87.10	8.00
Metaxa2	85.00	7.30	90.90	85.60	7.10
BLCA	83.10	6.80	81.80	83.60	9.60
SINTAX80	82.50	3.60	54.50	82.90	13.50
Q2_BLAST	78.10	11.10	81.80	78.60	10.20
Q2_VS	77.90	10.40	90.90	78.50	11.10
CT2	76.10	11.30	81.80	76.60	12.10
KNN	48.20	1.90	54.50	48.40	49.70

Table S50. TAXXI metrics computed for the dataset SP RDP ITS 97 at the species level.

Method	Accuracy	Misclassification Rate	Over-classification Rate	True Positive Rate	Under-classification Rate
Q2_BLAST	9.40	0.00	4.20	18.30	81.70
SINTAX80	8.80	1.70	24.00	56.70	41.70
Q2_VS	8.30	1.70	4.40	16.70	81.70
HiTaC_Filter	7.90	1.70	33.80	68.30	30.00
NBC80	6.50	6.70	44.60	71.70	21.70
SPINGO	6.40	3.30	44.70	71.70	25.00
RDP80	6.40	8.30	44.00	70.00	21.70
SINTAX50	5.80	11.70	52.90	75.00	13.30
Q2_SK	5.30	10.00	64.00	81.70	8.30
RDP50	4.40	13.30	77.90	81.70	5.00
NBC50	4.30	13.30	79.10	81.70	5.00
BLCA	4.20	5.00	60.50	61.70	33.30
HiTaC	3.70	13.30	100.00	86.70	0.00
TOP	3.70	13.30	100.00	86.70	0.00
BTOP	3.70	14.80	100.00	85.20	0.00
Microclass	3.60	15.00	100.00	85.00	0.00
KTOP	3.50	16.70	100.00	83.30	0.00
CT2	3.40	0.00	2.00	5.00	95.00
Q1	2.30	23.30	73.40	40.00	36.70
CT1	0.80	38.30	72.60	13.30	48.30
Metaxa2	0.70	0.00	45.90	8.30	91.70
KNN	0.00	0.00	0.20	0.00	100.00

Table S51. TAXXI metrics computed for the dataset SP RDP ITS 99 at the phylum level.

Method	Accuracy	Misclassification Rate	Over-classification Rate	True Positive Rate	Under-classification Rate
BTOP	100.00	0.00	.	100.00	0.00
CT1	100.00	0.00	.	100.00	0.00
CT2	100.00	0.00	.	100.00	0.00
HiTaC	100.00	0.00	.	100.00	0.00
HiTaC_Filter	100.00	0.00	.	100.00	0.00
KTOP	100.00	0.00	.	100.00	0.00
Metaxa2	100.00	0.00	.	100.00	0.00
Microclass	100.00	0.00	.	100.00	0.00
NBC50	100.00	0.00	.	100.00	0.00
NBC80	100.00	0.00	.	100.00	0.00
Q2_SK	100.00	0.00	.	100.00	0.00
RDP50	100.00	0.00	.	100.00	0.00
RDP80	100.00	0.00	.	100.00	0.00
SINTAX50	100.00	0.00	.	100.00	0.00
SINTAX80	100.00	0.00	.	100.00	0.00
TOP	100.00	0.00	.	100.00	0.00
Q2_BLAST	99.90	0.00	.	99.90	0.10
Q2_VS	99.90	0.00	.	99.90	0.10
KNN	99.80	0.00	.	99.80	0.20
Q1	99.70	0.00	.	99.70	0.30
BLCA	95.20	0.00	.	95.20	4.80
SPINGO	0.00	0.00	.	0.00	100.00

Table S52. TAXXI metrics computed for the dataset SP RDP ITS 99 at the class level.

Method	Accuracy	Misclassification Rate	Over-classification Rate	True Positive Rate	Under-classification Rate
BTOP	100.00	0.00	.	100.00	0.00
HiTaC	100.00	0.00	.	100.00	0.00
HiTaC_Filter	100.00	0.00	.	100.00	0.00
KTOP	100.00	0.00	.	100.00	0.00
Microclass	100.00	0.00	.	100.00	0.00
NBC50	100.00	0.00	.	100.00	0.00
NBC80	100.00	0.00	.	100.00	0.00
Q2_SK	100.00	0.00	.	100.00	0.00
RDP50	100.00	0.00	.	100.00	0.00
RDP80	100.00	0.00	.	100.00	0.00
SINTAX50	100.00	0.00	.	100.00	0.00
SINTAX80	100.00	0.00	.	100.00	0.00
TOP	100.00	0.00	.	100.00	0.00
CT1	99.90	0.10	.	99.90	0.00
Q1	99.70	0.10	.	99.70	0.30
Q2_BLAST	99.70	0.20	.	99.70	0.10
Q2_VS	99.70	0.20	.	99.70	0.10
CT2	99.70	0.30	.	99.70	0.10
Metaxa2	99.60	0.00	.	99.60	0.40
KNN	97.80	0.00	.	97.80	2.20
BLCA	95.20	0.00	.	95.20	4.80
SPINGO	0.00	0.00	.	0.00	100.00

Table S53. TAXXI metrics computed for the dataset SP RDP ITS 99 at the order level.

Method	Accuracy	Misclassification Rate	Over-classification Rate	True Positive Rate	Under-classification Rate
BTOP	100.00	0.00	.	100.00	0.00
HiTaC	100.00	0.00	.	100.00	0.00
HiTaC_Filter	100.00	0.00	.	100.00	0.00
Microclass	100.00	0.00	.	100.00	0.00
Q2_SK	99.90	0.00	.	99.90	0.00
SINTAX50	99.90	0.00	.	99.90	0.00
SINTAX80	99.90	0.00	.	99.90	0.10
KTOP	99.90	0.10	.	99.90	0.00
NBC50	99.90	0.10	.	99.90	0.00
NBC80	99.90	0.10	.	99.90	0.00
RDP50	99.90	0.10	.	99.90	0.00
RDP80	99.90	0.10	.	99.90	0.00
TOP	99.90	0.10	.	99.90	0.00
Q1	99.40	0.30	.	99.40	0.30
CT1	99.30	0.60	.	99.30	0.10
Q2_VS	99.00	0.70	.	99.00	0.30
Q2_BLAST	98.90	0.80	.	98.90	0.30
Metaxa2	98.70	0.00	.	98.70	1.30
CT2	98.50	1.00	.	98.50	0.60
BLCA	95.20	0.00	.	95.20	4.80
KNN	93.20	0.00	.	93.20	6.80
SPINGO	0.00	0.00	.	0.00	100.00

Table S54. TAXXI metrics computed for the dataset SP RDP ITS 99 at the family level.

Method	Accuracy	Misclassification Rate	Over-classification Rate	True Positive Rate	Under-classification Rate
BTOP	100.00	0.00	.	100.00	0.00
HiTaC	100.00	0.00	.	100.00	0.00
HiTaC_Filter	99.90	0.00	.	99.90	0.00
NBC80	99.90	0.00	.	99.90	0.10
SINTAX50	99.90	0.00	.	99.90	0.10
SPINGO	99.90	0.00	.	99.90	0.10
KTOP	99.90	0.10	.	99.90	0.00
Microclass	99.90	0.10	.	99.90	0.00
NBC50	99.90	0.10	.	99.90	0.00
RDP50	99.90	0.10	.	99.90	0.00
TOP	99.90	0.10	.	99.90	0.00
Q2_SK	99.90	0.10	.	99.90	0.10
RDP80	99.90	0.10	.	99.90	0.10
SINTAX80	99.80	0.00	.	99.80	0.20
Q1	99.10	0.60	.	99.10	0.30
CT1	97.90	1.70	.	97.90	0.30
Metaxa2	96.20	0.00	.	96.20	3.80
Q2_VS	96.10	2.30	.	96.10	1.60
Q2_BLAST	95.60	2.30	.	95.60	2.10
BLCA	95.10	0.00	.	95.10	4.80
CT2	93.80	2.90	.	93.80	3.20
KNN	80.80	0.00	.	80.80	19.10

Table S55. TAXXI metrics computed for the dataset SP RDP ITS 99 at the genus level.

Method	Accuracy	Misclassification Rate	Over-classification Rate	True Positive Rate	Under-classification Rate
HiTaC	99.50	0.50	.	99.50	0.00
TOP	99.50	0.50	.	99.50	0.00
BTOP	99.40	0.60	.	99.40	0.00
KTOP	99.40	0.60	.	99.40	0.00
Microclass	99.40	0.60	.	99.40	0.00
RDP50	99.30	0.60	.	99.30	0.10
NBC50	99.30	0.70	.	99.30	0.10
Q2_SK	99.20	0.40	.	99.20	0.30
SINTAX50	99.20	0.40	.	99.20	0.40
SPINGO	99.00	0.20	.	99.00	0.80
RDP80	98.90	0.30	.	98.90	0.90
HiTaC_Filter	98.80	0.10	.	98.80	1.10
NBC80	98.80	0.20	.	98.80	1.00
SINTAX80	98.00	0.10	.	98.00	2.00
Q1	95.50	3.30	.	95.50	1.20
BLCA	94.30	0.30	.	94.30	5.40
CT1	92.20	6.00	.	92.20	1.70
Metaxa2	89.00	0.30	.	89.00	10.60
Q2_VS	81.40	6.80	.	81.40	11.80
Q2_BLAST	80.20	7.70	.	80.20	12.20
CT2	77.10	7.60	.	77.10	15.40
KNN	48.00	0.00	.	48.00	52.00

Table S56. TAXXI metrics computed for the dataset SP RDP ITS 99 at the species level.

Method	Accuracy	Misclassification Rate	Over-classification Rate	True Positive Rate	Under-classification Rate
HiTaC	73.00	10.60	100.00	89.40	0.00
BTOP	72.60	11.30	100.00	88.70	0.00
Microclass	72.30	11.40	100.00	88.60	0.00
TOP	72.30	11.40	100.00	88.60	0.00
SINTAX50	72.20	6.60	70.40	83.60	9.70
KTOP	71.60	12.20	100.00	87.80	0.00
SPINGO	71.30	3.50	57.60	80.60	15.90
Q2_SK	70.10	8.40	74.90	82.00	9.60
HiTaC_Filter	70.00	2.70	49.00	77.80	19.50
NBC50	69.70	13.30	91.20	84.10	2.60
RDP50	69.60	13.30	89.50	83.70	3.00
NBC80	68.60	7.00	64.30	78.50	14.50
RDP80	68.50	6.40	63.60	78.30	15.30
BLCA	67.20	7.90	66.50	77.30	14.80
SINTAX80	65.40	1.30	37.70	71.00	27.70
Q1	39.40	21.70	65.70	45.20	33.10
CT1	28.80	28.50	68.20	33.20	38.30
Metaxa2	25.90	2.80	42.00	28.30	68.90
Q2_VS	8.80	2.10	4.50	8.90	89.10
Q2_BLAST	7.00	1.70	4.70	7.10	91.30
CT2	3.60	0.90	3.30	3.60	95.50
KNN	0.00	0.00	0.00	0.00	100.00

Table S57. TAXXI metrics computed for the dataset SP RDP ITS 100 at the phylum level.

Method	Accuracy	Misclassification Rate	Over-classification Rate	True Positive Rate	Under-classification Rate
BTOP	100.00	0.00	.	100.00	0.00
CT1	100.00	0.00	.	100.00	0.00
CT2	100.00	0.00	.	100.00	0.00
HiTaC	100.00	0.00	.	100.00	0.00
HiTaC_Filter	100.00	0.00	.	100.00	0.00
KTOP	100.00	0.00	.	100.00	0.00
Metaxa2	100.00	0.00	.	100.00	0.00
Microclass	100.00	0.00	.	100.00	0.00
Q1	100.00	0.00	.	100.00	0.00
Q2_BLAST	100.00	0.00	.	100.00	0.00
Q2_SK	100.00	0.00	.	100.00	0.00
Q2_VS	100.00	0.00	.	100.00	0.00
RDP50	100.00	0.00	.	100.00	0.00
RDP80	100.00	0.00	.	100.00	0.00
SINTAX50	100.00	0.00	.	100.00	0.00
SINTAX80	100.00	0.00	.	100.00	0.00
TOP	100.00	0.00	.	100.00	0.00
KNN	99.80	0.00	.	99.80	0.20
BLCA	0.00	0.00	.	0.00	100.00
SPINGO	0.00	0.00	.	0.00	100.00

Table S58. TAXXI metrics computed for the dataset SP RDP ITS 100 at the class level.

Method	Accuracy	Misclassification Rate	Over-classification Rate	True Positive Rate	Under-classification Rate
BTOP	100.00	0.00	.	100.00	0.00
HiTaC	100.00	0.00	.	100.00	0.00
HiTaC_Filter	100.00	0.00	.	100.00	0.00
KTOP	100.00	0.00	.	100.00	0.00
Microclass	100.00	0.00	.	100.00	0.00
Q1	100.00	0.00	.	100.00	0.00
Q2_SK	100.00	0.00	.	100.00	0.00
RDP50	100.00	0.00	.	100.00	0.00
RDP80	100.00	0.00	.	100.00	0.00
SINTAX50	100.00	0.00	.	100.00	0.00
SINTAX80	100.00	0.00	.	100.00	0.00
TOP	100.00	0.00	.	100.00	0.00
Metaxa2	99.90	0.00	.	99.90	0.10
CT1	99.90	0.10	.	99.90	0.00
Q2_BLAST	99.70	0.20	.	99.70	0.00
Q2_VS	99.70	0.20	.	99.70	0.00
CT2	99.70	0.30	.	99.70	0.00
KNN	98.20	0.00	.	98.20	1.80
BLCA	0.00	0.00	.	0.00	100.00
SPINGO	0.00	0.00	.	0.00	100.00

Table S59. TAXXI metrics computed for the dataset SP RDP ITS 100 at the order level.

Method	Accuracy	Misclassification Rate	Over-classification Rate	True Positive Rate	Under-classification Rate
BTOP	100.00	0.00	.	100.00	0.00
HiTaC	100.00	0.00	.	100.00	0.00
HiTaC_Filter	100.00	0.00	.	100.00	0.00
KTOP	100.00	0.00	.	100.00	0.00
Microclass	100.00	0.00	.	100.00	0.00
Q1	100.00	0.00	.	100.00	0.00
Q2_SK	100.00	0.00	.	100.00	0.00
RDP50	100.00	0.00	.	100.00	0.00
RDP80	100.00	0.00	.	100.00	0.00
SINTAX50	100.00	0.00	.	100.00	0.00
SINTAX80	100.00	0.00	.	100.00	0.00
TOP	100.00	0.00	.	100.00	0.00
CT1	99.80	0.20	.	99.80	0.00
Metaxa2	99.70	0.00	.	99.70	0.30
Q2_VS	99.00	0.60	.	99.00	0.30
Q2_BLAST	99.00	0.60	.	99.00	0.40
CT2	98.70	0.80	.	98.70	0.50
KNN	94.40	0.00	.	94.40	5.60
BLCA	0.00	0.00	.	0.00	100.00
SPINGO	0.00	0.00	.	0.00	100.00

Table S60. TAXXI metrics computed for the dataset SP RDP ITS 100 at the family level.

Method	Accuracy	Misclassification Rate	Over-classification Rate	True Positive Rate	Under-classification Rate
BTOP	100.00	0.00	.	100.00	0.00
HiTaC	100.00	0.00	.	100.00	0.00
HiTaC_Filter	100.00	0.00	.	100.00	0.00
KTOP	100.00	0.00	.	100.00	0.00
Microclass	100.00	0.00	.	100.00	0.00
Q2_SK	100.00	0.00	.	100.00	0.00
RDP50	100.00	0.00	.	100.00	0.00
RDP80	100.00	0.00	.	100.00	0.00
SINTAX50	100.00	0.00	.	100.00	0.00
SINTAX80	100.00	0.00	.	100.00	0.00
SPINGO	100.00	0.00	.	100.00	0.00
TOP	100.00	0.00	.	100.00	0.00
Q1	99.80	0.20	.	99.80	0.00
CT1	99.50	0.50	.	99.50	0.10
Metaxa2	99.20	0.00	.	99.20	0.80
Q2_VS	96.40	2.00	.	96.40	1.60
Q2_BLAST	96.10	2.10	.	96.10	1.80
CT2	95.80	2.20	.	95.80	2.10
KNN	84.80	0.00	.	84.80	15.20
BLCA	0.00	0.00	.	0.00	100.00

Table S61. TAXXI metrics computed for the dataset SP RDP ITS 100 at the genus level.

Method	Accuracy	Misclassification Rate	Over-classification Rate	True Positive Rate	Under-classification Rate
BTOP	100.00	0.00	.	100.00	0.00
HiTaC	100.00	0.00	.	100.00	0.00
HiTaC_Filter	100.00	0.00	.	100.00	0.00
KTOP	100.00	0.00	.	100.00	0.00
Microclass	100.00	0.00	.	100.00	0.00
Q2_SK	100.00	0.00	.	100.00	0.00
RDP50	100.00	0.00	.	100.00	0.00
SINTAX50	100.00	0.00	.	100.00	0.00
SPINGO	100.00	0.00	.	100.00	0.00
TOP	100.00	0.00	.	100.00	0.00
RDP80	99.90	0.00	.	99.90	0.10
SINTAX80	99.80	0.00	.	99.80	0.20
Q1	99.00	0.80	.	99.00	0.20
CT1	98.20	1.50	.	98.20	0.30
Metaxa2	97.70	0.00	.	97.70	2.30
Q2_VS	84.30	6.20	.	84.30	9.50
Q2_BLAST	83.60	6.70	.	83.60	9.70
CT2	82.90	6.60	.	82.90	10.50
KNN	57.60	0.00	.	57.60	42.40
BLCA	0.00	0.00	.	0.00	100.00

Table S62. TAXXI metrics computed for the dataset SP RDP ITS 100 at the species level.

Method	Accuracy	Misclassification Rate	Over-classification Rate	True Positive Rate	Under-classification Rate
BTOP	100.00	0.00	.	100.00	0.00
HiTaC	100.00	0.00	.	100.00	0.00
TOP	99.60	0.40	.	99.60	0.00
KTOP	99.40	0.60	.	99.40	0.00
Microclass	99.40	0.60	.	99.40	0.00
HiTaC_Filter	97.80	0.00	.	97.80	2.20
SINTAX50	97.50	0.40	.	97.50	2.10
SPINGO	96.80	0.00	.	96.80	3.20
RDP50	96.20	2.90	.	96.20	0.90
Q2_SK	95.30	0.80	.	95.30	3.80
RDP80	93.00	1.50	.	93.00	5.50
SINTAX80	86.20	0.00	.	86.20	13.80
Q1	83.70	7.80	.	83.70	8.50
CT1	82.70	8.70	.	82.70	8.60
Metaxa2	78.00	0.00	.	78.00	22.00
Q2_VS	10.90	1.30	.	10.90	87.80
Q2_BLAST	9.30	1.30	.	9.30	89.40
CT2	8.50	1.10	.	8.50	90.40
KNN	0.10	0.00	.	0.10	99.90
BLCA	0.00	0.00	.	0.00	100.00

Table S63. Machine learning metrics computed for the dataset SP RDP ITS 90 at the phylum level.

Method	Accuracy	Balanced Accuracy	F1-score Micro	F1-score Macro	F1-score Weighted	Precision Micro	Precision Macro	Precision Weighted	Recall Micro	Recall Macro	Recall Weighted	Jaccard Micro	Jaccard Macro	Jaccard Weighted
BTOP	100.00	100.00	100.00	100.00	100.00	100.00	100.00	100.00	100.00	100.00	100.00	100.00	100.00	100.00
CT1	100.00	100.00	100.00	100.00	100.00	100.00	100.00	100.00	100.00	100.00	100.00	100.00	100.00	100.00
CT2	100.00	100.00	100.00	100.00	100.00	100.00	100.00	100.00	100.00	100.00	100.00	100.00	100.00	100.00
HiTaC	100.00	100.00	100.00	100.00	100.00	100.00	100.00	100.00	100.00	100.00	100.00	100.00	100.00	100.00
HiTaC_Filter	100.00	100.00	100.00	100.00	100.00	100.00	100.00	100.00	100.00	100.00	100.00	100.00	100.00	100.00
KTOP	100.00	100.00	100.00	100.00	100.00	100.00	100.00	100.00	100.00	100.00	100.00	100.00	100.00	100.00
Metaxa2	100.00	100.00	100.00	100.00	100.00	100.00	100.00	100.00	100.00	100.00	100.00	100.00	100.00	100.00
Microclass	100.00	100.00	100.00	100.00	100.00	100.00	100.00	100.00	100.00	100.00	100.00	100.00	100.00	100.00
NBC50	100.00	100.00	100.00	100.00	100.00	100.00	100.00	100.00	100.00	100.00	100.00	100.00	100.00	100.00
NBC80	100.00	100.00	100.00	100.00	100.00	100.00	100.00	100.00	100.00	100.00	100.00	100.00	100.00	100.00
Q2_SK	100.00	100.00	100.00	100.00	100.00	100.00	100.00	100.00	100.00	100.00	100.00	100.00	100.00	100.00
RDP50	100.00	100.00	100.00	100.00	100.00	100.00	100.00	100.00	100.00	100.00	100.00	100.00	100.00	100.00
RDP80	100.00	100.00	100.00	100.00	100.00	100.00	100.00	100.00	100.00	100.00	100.00	100.00	100.00	100.00
SINTAX50	100.00	100.00	100.00	100.00	100.00	100.00	100.00	100.00	100.00	100.00	100.00	100.00	100.00	100.00
SINTAX80	100.00	100.00	100.00	100.00	100.00	100.00	100.00	100.00	100.00	100.00	100.00	100.00	100.00	100.00
TOP	100.00	100.00	100.00	100.00	100.00	100.00	100.00	100.00	100.00	100.00	100.00	100.00	100.00	100.00
KNN	99.90	98.15	99.90	74.29	99.95	99.90	75.00	100.00	99.90	73.61	99.90	99.79	73.61	99.90
Q2_VS	99.84	99.91	99.84	74.97	99.92	99.84	75.00	100.00	99.84	74.93	99.84	99.69	74.93	99.84
Q2_BLAST	99.74	99.85	99.74	74.94	99.87	99.74	75.00	100.00	99.74	74.89	99.74	99.48	74.89	99.74
Q1	92.76	95.16	92.76	73.12	96.24	92.76	75.00	100.00	92.76	71.37	92.76	86.51	71.37	92.76
BLCA	92.51	82.79	92.51	67.31	96.03	92.51	75.00	100.00	92.51	62.09	92.51	86.06	62.09	92.51
SPINGO	0.00	0.00	0.00	0.00	0.00	0.00	0.00	0.00	0.00	0.00	0.00	0.00	0.00	0.00

Table S64. Machine learning metrics computed for the dataset SP RDP ITS 90 at the class level.

Method	Accuracy	Balanced Accuracy	F1-score Micro	F1-score Macro	F1-score Weighted	Precision Micro	Precision Macro	Precision Weighted	Recall Micro	Recall Macro	Recall Weighted	Jaccard Micro	Jaccard Macro	Jaccard Weighted
HiTaC	99.95	99.97	99.95	99.97	99.95	99.95	99.98	99.95	99.95	99.97	99.95	99.90	99.95	99.90
HiTaC_Filter	99.95	99.97	99.95	99.97	99.95	99.95	99.98	99.95	99.95	99.97	99.95	99.90	99.95	99.90
CT2	99.90	99.95	99.90	99.95	99.90	99.90	99.95	99.90	99.90	99.95	99.90	99.79	99.90	99.79
BTOP	99.85	99.04	99.85	99.12	99.85	99.85	99.33	99.86	99.85	99.04	99.85	99.70	98.37	99.71
Microclass	99.84	99.04	99.84	99.12	99.84	99.84	99.33	99.86	99.84	99.04	99.84	99.69	98.37	99.70
Q2_SK	99.84	99.04	99.84	99.12	99.84	99.84	99.33	99.86	99.84	99.04	99.84	99.69	98.37	99.70
Q2_VS	99.79	99.92	99.79	94.69	99.87	99.79	94.72	99.95	99.79	94.66	99.79	99.59	94.64	99.74
TOP	99.74	99.01	99.74	99.02	99.74	99.74	99.17	99.76	99.74	99.01	99.74	99.48	98.17	99.50
Q2_BLAST	99.69	99.88	99.69	94.67	99.82	99.69	94.72	99.95	99.69	94.62	99.69	99.38	94.61	99.64
Metaxa2	99.69	98.95	99.69	99.05	99.69	99.69	99.28	99.70	99.69	98.95	99.69	99.38	98.23	99.39
RDP50	99.59	98.93	99.59	98.83	99.59	99.59	98.88	99.61	99.59	98.93	99.59	99.18	97.81	99.20
KTOP	99.53	98.90	99.53	98.90	99.53	99.53	99.04	99.55	99.53	98.90	99.53	99.07	97.94	99.09
NBC80	99.53	98.90	99.53	94.17	99.74	99.53	94.72	99.95	99.53	93.69	99.53	99.07	93.68	99.48
RDP80	99.53	98.90	99.53	94.17	99.74	99.53	94.72	99.95	99.53	93.69	99.53	99.07	93.68	99.48
SINTAX80	99.53	98.90	99.53	94.17	99.74	99.53	94.72	99.95	99.53	93.69	99.53	99.07	93.68	99.48
SINTAX50	99.53	98.90	99.53	94.14	99.66	99.53	94.67	99.79	99.53	93.69	99.53	99.07	93.63	99.33
CT1	99.53	98.90	99.53	93.79	99.56	99.53	94.00	99.60	99.53	93.69	99.53	99.07	92.96	99.14
NBC50	99.53	98.90	99.53	93.78	99.59	99.53	93.96	99.65	99.53	93.69	99.53	99.07	92.92	99.19
KNN	98.60	91.56	98.60	88.95	99.15	98.60	94.72	99.95	98.60	86.74	98.60	97.25	86.73	98.55
Q1	92.61	92.46	92.61	90.40	96.03	92.61	94.10	99.86	92.61	87.59	92.61	86.24	86.96	92.47
BLCA	92.35	91.80	92.35	90.00	95.71	92.35	94.10	99.85	92.35	86.96	92.35	85.79	86.33	92.21
SPINGO	0.00	0.00	0.00	0.00	0.00	0.00	0.00	0.00	0.00	0.00	0.00	0.00	0.00	0.00

Table S65. Machine learning metrics computed for the dataset SP RDP ITS 90 at the order level.

Method	Accuracy	Balanced Accuracy	F1-score Micro	F1-score Macro	F1-score Weighted	Precision Micro	Precision Macro	Precision Weighted	Recall Micro	Recall Macro	Recall Weighted	Jaccard Micro	Jaccard Macro	Jaccard Weighted
Microclass	98.24	94.21	98.24	92.80	98.29	98.24	93.34	98.46	98.24	92.66	98.24	96.55	91.38	97.24
HiTaC	98.19	90.49	98.19	89.42	98.07	98.19	90.38	98.07	98.19	89.01	98.19	96.45	88.04	97.10
HiTaC_Filter	98.19	90.49	98.19	88.01	98.17	98.19	88.99	98.26	98.19	87.57	98.19	96.45	86.69	97.29
Q2_VS	98.14	89.79	98.14	87.08	98.20	98.14	87.39	98.30	98.14	86.89	98.14	96.35	86.03	97.34
TOP	98.09	94.09	98.09	92.65	98.14	98.09	93.17	98.31	98.09	92.55	98.09	96.25	91.09	96.95
Q2_SK	98.09	93.78	98.09	91.13	98.28	98.09	91.93	98.59	98.09	90.76	98.09	96.25	89.59	97.22
BTOP	98.07	93.84	98.07	92.57	98.10	98.07	93.31	98.28	98.07	92.30	98.07	96.21	90.99	96.89
Q2_BLAST	97.98	91.02	97.98	88.47	98.11	97.98	89.06	98.30	97.98	88.08	97.98	96.05	87.27	97.13
KTOP	97.93	94.11	97.93	92.47	97.98	97.93	92.83	98.19	97.93	92.56	97.93	95.95	90.77	96.67
RDP50	97.93	94.04	97.93	91.07	98.02	97.93	91.55	98.26	97.93	91.00	97.93	95.95	89.46	96.74
CT1	97.88	94.02	97.88	90.99	97.95	97.88	91.42	98.16	97.88	90.99	97.88	95.85	89.32	96.63
NBC50	97.88	94.01	97.88	91.14	98.04	97.88	91.70	98.35	97.88	90.98	97.88	95.85	89.59	96.78
CT2	97.88	86.64	97.88	84.42	97.88	97.88	85.58	98.01	97.88	83.85	97.88	95.85	82.78	96.84
RDP80	97.83	93.74	97.83	91.34	98.25	97.83	92.35	98.80	97.83	90.71	97.83	95.75	89.97	97.18
SINTAX50	97.83	93.62	97.83	91.20	98.18	97.83	92.22	98.67	97.83	90.60	97.83	95.75	89.73	97.05
NBC80	97.78	93.32	97.78	91.11	98.22	97.78	92.35	98.80	97.78	90.31	97.78	95.65	89.56	97.13
Metaxa2	97.21	92.23	97.21	89.45	97.18	97.21	91.13	97.88	97.21	89.26	97.21	94.57	87.38	95.69
SINTAX80	97.11	89.60	97.11	87.75	97.75	97.11	89.12	98.55	97.11	86.71	97.11	94.37	85.97	96.46
KNN	93.23	68.33	93.23	68.02	94.70	93.23	72.18	96.81	93.23	66.12	93.23	87.32	65.74	92.80
Q1	91.37	87.92	91.37	87.73	94.68	91.37	92.02	98.75	91.37	85.09	91.37	84.11	84.08	90.71
BLCA	90.44	86.70	90.44	86.87	93.71	90.44	91.73	98.39	90.44	83.90	90.44	82.55	82.73	89.49
SPINGO	0.00	0.00	0.00	0.00	0.00	0.00	0.00	0.00	0.00	0.00	0.00	0.00	0.00	0.00

Table S66. Machine learning metrics computed for the dataset SP RDP ITS 90 at the family level.

Method	Accuracy	Balanced Accuracy	F1-score Micro	F1-score Macro	F1-score Weighted	Precision Micro	Precision Macro	Precision Weighted	Recall Micro	Recall Macro	Recall Weighted	Jaccard Micro	Jaccard Macro	Jaccard Weighted
HiTaC	95.09	87.88	95.09	84.95	94.86	95.09	85.89	95.51	95.09	85.68	95.09	90.64	82.74	92.60
HiTaC_Filter	93.95	86.27	93.95	83.33	94.47	93.95	85.37	96.39	93.95	83.60	93.95	88.60	81.18	92.40
TOP	93.90	89.21	93.90	84.49	93.77	93.90	85.44	95.14	93.90	85.91	93.90	88.50	82.01	90.90
Microclass	93.85	89.90	93.85	84.35	93.92	93.85	84.74	95.02	93.85	85.52	93.85	88.41	81.67	90.87
BTOP	93.75	88.95	93.75	84.30	93.87	93.75	85.58	95.38	93.75	85.65	93.75	88.23	81.39	90.89
KTOP	93.44	88.40	93.44	83.12	93.18	93.44	84.02	94.61	93.44	84.60	93.44	87.68	80.41	89.98
CT1	93.33	87.73	93.33	81.73	93.23	93.33	82.41	94.63	93.33	83.45	93.33	87.50	78.84	90.00
RDP50	93.23	88.85	93.23	84.01	93.31	93.23	85.26	95.12	93.23	85.03	93.23	87.32	81.34	90.13
NBC50	93.18	88.83	93.18	84.08	93.40	93.18	85.72	95.46	93.18	85.02	93.18	87.23	81.54	90.37
Q2_SK	93.13	89.02	93.13	85.43	93.92	93.13	87.32	96.14	93.13	85.73	93.13	87.14	82.64	90.88
SINTAX50	92.66	86.44	92.66	82.90	93.46	92.66	85.14	95.97	92.66	83.24	92.66	86.33	80.39	90.64
Q2_VS	91.73	81.38	91.73	76.48	92.00	91.73	78.97	94.33	91.73	77.89	91.73	84.73	73.63	89.32
NBC80	91.63	86.39	91.63	83.79	92.90	91.63	86.47	96.00	91.63	83.71	91.63	84.55	81.19	90.04
RDP80	91.52	86.51	91.52	83.78	92.80	91.52	86.42	95.97	91.52	83.82	91.52	84.37	81.30	89.97
Q2_BLAST	91.37	82.22	91.37	77.09	91.90	91.37	78.94	93.71	91.37	78.21	91.37	84.11	74.17	88.85
SPINGO	91.27	86.17	91.27	83.28	92.74	91.27	86.36	95.99	91.27	82.98	91.27	83.94	80.34	89.64
CT2	91.27	79.08	91.27	74.12	91.51	91.27	76.16	93.54	91.27	75.22	91.27	83.94	71.10	88.62
Metaxa2	90.49	83.06	90.49	79.90	90.85	90.49	82.14	93.72	90.49	80.48	90.49	82.63	76.74	87.22
SINTAX80	87.65	77.13	87.65	77.70	90.33	87.65	83.05	95.64	87.65	76.15	87.65	78.01	74.28	86.71
Q1	87.29	81.03	87.29	79.45	90.37	87.29	83.41	95.34	87.29	78.03	87.29	77.44	75.12	85.07
BLCA	86.93	81.64	86.93	79.39	89.98	86.93	83.12	95.21	86.93	78.61	86.93	76.87	75.13	84.88
KNN	80.16	53.99	80.16	55.66	83.55	80.16	61.78	90.70	80.16	53.31	80.16	66.88	52.70	79.56

Table S67. Machine learning metrics computed for the dataset SP RDP ITS 90 at the genus level.

Method	Accuracy	Balanced Accuracy	F1-score Micro	F1-score Macro	F1-score Weighted	Precision Micro	Precision Macro	Precision Weighted	Recall Micro	Recall Macro	Recall Weighted	Jaccard Micro	Jaccard Macro	Jaccard Weighted
HiTaC	69.46	55.89	69.46	43.88	65.82	69.46	43.74	65.51	69.46	47.45	69.46	53.21	41.25	61.51
BTOP	63.65	57.26	63.65	43.26	63.08	63.65	44.23	68.20	63.65	45.81	63.65	46.68	40.44	57.69
Microclass	62.74	56.93	62.74	42.74	61.97	62.74	43.30	64.98	62.74	45.45	62.74	45.71	39.92	56.97
KTOP	62.43	56.09	62.43	42.29	61.26	62.43	43.13	64.00	62.43	45.14	62.43	45.38	39.43	56.14
TOP	62.38	56.63	62.38	42.43	61.28	62.38	43.03	64.04	62.38	45.30	62.38	45.32	39.73	56.32
CT1	61.45	51.53	61.45	38.34	59.95	61.45	38.46	61.71	61.45	41.55	61.45	44.35	35.57	54.99
NBC50	61.34	55.04	61.34	44.13	60.65	61.34	45.57	64.31	61.34	46.25	61.34	44.24	41.25	55.92
RDP50	61.09	54.83	61.09	44.11	60.59	61.09	45.41	64.37	61.09	46.27	61.09	43.97	41.23	55.70
HiTaC_Filter	59.95	46.92	59.95	42.51	58.84	59.95	43.94	63.32	59.95	44.36	59.95	42.80	39.97	54.80
Q2_SK	59.28	53.63	59.28	43.23	59.27	59.28	44.92	64.77	59.28	45.25	59.28	42.12	40.56	54.52
Q1	58.81	52.45	58.81	41.91	59.78	58.81	44.87	67.48	58.81	43.20	58.81	41.65	38.49	53.64
SINTAX50	57.52	49.40	57.52	42.74	57.55	57.52	44.55	61.66	57.52	44.58	57.52	40.37	40.05	53.01
Metaxa2	56.74	47.63	56.74	39.35	56.82	56.74	41.43	62.19	56.74	41.02	56.74	39.61	36.75	51.90
BLCA	56.23	49.12	56.23	40.17	57.93	56.23	43.20	66.30	56.23	41.03	56.23	39.11	36.97	52.27
Q2_VS	55.50	42.52	55.50	34.92	53.30	55.50	34.73	54.64	55.50	38.45	55.50	38.41	32.55	49.45
SPINGO	53.39	47.62	53.39	41.90	54.43	53.39	44.16	60.34	53.39	43.07	53.39	36.41	39.25	49.70
Q2_BLAST	53.13	40.53	53.13	33.79	51.69	53.13	34.25	54.26	53.13	36.73	53.13	36.17	31.23	47.16
NBC80	52.66	46.40	52.66	41.73	53.56	52.66	44.34	60.25	52.66	42.71	52.66	35.74	39.22	49.31
RDP80	52.61	46.95	52.61	42.37	53.64	52.61	44.98	60.72	52.61	43.21	52.61	35.69	39.98	49.42
CT2	52.56	37.55	52.56	31.00	50.70	52.56	31.10	52.81	52.56	34.26	52.56	35.65	28.57	46.47
SINTAX80	44.08	34.72	44.08	33.29	45.93	44.08	36.60	53.02	44.08	33.28	44.08	28.27	31.16	41.95
KNN	31.01	16.11	31.01	16.20	33.47	31.01	18.32	39.74	31.01	15.80	31.01	18.35	14.80	29.76

Table S68. Machine learning metrics computed for the dataset SP RDP ITS 90 at the species level.

Method	Accuracy	Balanced Accuracy	F1-score Micro	F1-score Macro	F1-score Weighted	Precision Micro	Precision Macro	Precision Weighted	Recall Micro	Recall Macro	Recall Weighted	Jaccard Micro	Jaccard Macro	Jaccard Weighted
BLCA	0.00	0.00	0.00	0.00	0.00	0.00	0.00	0.00	0.00	0.00	0.00	0.00	0.00	0.00
BTOP	0.00	0.00	0.00	0.00	0.00	0.00	0.00	0.00	0.00	0.00	0.00	0.00	0.00	0.00
CT1	0.00	0.00	0.00	0.00	0.00	0.00	0.00	0.00	0.00	0.00	0.00	0.00	0.00	0.00
CT2	0.00	0.00	0.00	0.00	0.00	0.00	0.00	0.00	0.00	0.00	0.00	0.00	0.00	0.00
HiTaC	0.00	0.00	0.00	0.00	0.00	0.00	0.00	0.00	0.00	0.00	0.00	0.00	0.00	0.00
HiTaC_Filter	0.00	0.00	0.00	0.00	0.00	0.00	0.00	0.00	0.00	0.00	0.00	0.00	0.00	0.00
KNN	0.00	0.00	0.00	0.00	0.00	0.00	0.00	0.00	0.00	0.00	0.00	0.00	0.00	0.00
KTOP	0.00	0.00	0.00	0.00	0.00	0.00	0.00	0.00	0.00	0.00	0.00	0.00	0.00	0.00
Metaxa2	0.00	0.00	0.00	0.00	0.00	0.00	0.00	0.00	0.00	0.00	0.00	0.00	0.00	0.00
Microclass	0.00	0.00	0.00	0.00	0.00	0.00	0.00	0.00	0.00	0.00	0.00	0.00	0.00	0.00
NBC50	0.00	0.00	0.00	0.00	0.00	0.00	0.00	0.00	0.00	0.00	0.00	0.00	0.00	0.00
NBC80	0.00	0.00	0.00	0.00	0.00	0.00	0.00	0.00	0.00	0.00	0.00	0.00	0.00	0.00
Q1	0.00	0.00	0.00	0.00	0.00	0.00	0.00	0.00	0.00	0.00	0.00	0.00	0.00	0.00
Q2_BLAST	0.00	0.00	0.00	0.00	0.00	0.00	0.00	0.00	0.00	0.00	0.00	0.00	0.00	0.00
Q2_SK	0.00	0.00	0.00	0.00	0.00	0.00	0.00	0.00	0.00	0.00	0.00	0.00	0.00	0.00
Q2_VS	0.00	0.00	0.00	0.00	0.00	0.00	0.00	0.00	0.00	0.00	0.00	0.00	0.00	0.00
RDP50	0.00	0.00	0.00	0.00	0.00	0.00	0.00	0.00	0.00	0.00	0.00	0.00	0.00	0.00
RDP80	0.00	0.00	0.00	0.00	0.00	0.00	0.00	0.00	0.00	0.00	0.00	0.00	0.00	0.00
SINTAX50	0.00	0.00	0.00	0.00	0.00	0.00	0.00	0.00	0.00	0.00	0.00	0.00	0.00	0.00
SINTAX80	0.00	0.00	0.00	0.00	0.00	0.00	0.00	0.00	0.00	0.00	0.00	0.00	0.00	0.00
SPINGO	0.00	0.00	0.00	0.00	0.00	0.00	0.00	0.00	0.00	0.00	0.00	0.00	0.00	0.00
TOP	0.00	0.00	0.00	0.00	0.00	0.00	0.00	0.00	0.00	0.00	0.00	0.00	0.00	0.00

Table S69. Machine learning metrics computed for the dataset SP RDP ITS 95 at the phylum level.

Method	Accuracy	Balanced Accuracy	F1-score Micro	F1-score Macro	F1-score Weighted	Precision Micro	Precision Macro	Precision Weighted	Recall Micro	Recall Macro	Recall Weighted	Jaccard Micro	Jaccard Macro	Jaccard Weighted
BTOP	100.00	100.00	100.00	100.00	100.00	100.00	100.00	100.00	100.00	100.00	100.00	100.00	100.00	100.00
CT1	100.00	100.00	100.00	100.00	100.00	100.00	100.00	100.00	100.00	100.00	100.00	100.00	100.00	100.00
CT2	100.00	100.00	100.00	100.00	100.00	100.00	100.00	100.00	100.00	100.00	100.00	100.00	100.00	100.00
HiTaC	100.00	100.00	100.00	100.00	100.00	100.00	100.00	100.00	100.00	100.00	100.00	100.00	100.00	100.00
HiTaC_Filter	100.00	100.00	100.00	100.00	100.00	100.00	100.00	100.00	100.00	100.00	100.00	100.00	100.00	100.00
KNN	100.00	100.00	100.00	100.00	100.00	100.00	100.00	100.00	100.00	100.00	100.00	100.00	100.00	100.00
KTOP	100.00	100.00	100.00	100.00	100.00	100.00	100.00	100.00	100.00	100.00	100.00	100.00	100.00	100.00
Metaxa2	100.00	100.00	100.00	100.00	100.00	100.00	100.00	100.00	100.00	100.00	100.00	100.00	100.00	100.00
Microclass	100.00	100.00	100.00	100.00	100.00	100.00	100.00	100.00	100.00	100.00	100.00	100.00	100.00	100.00
NBC50	100.00	100.00	100.00	100.00	100.00	100.00	100.00	100.00	100.00	100.00	100.00	100.00	100.00	100.00
NBC80	100.00	100.00	100.00	100.00	100.00	100.00	100.00	100.00	100.00	100.00	100.00	100.00	100.00	100.00
Q2_BLAST	100.00	100.00	100.00	100.00	100.00	100.00	100.00	100.00	100.00	100.00	100.00	100.00	100.00	100.00
Q2_SK	100.00	100.00	100.00	100.00	100.00	100.00	100.00	100.00	100.00	100.00	100.00	100.00	100.00	100.00
Q2_VS	100.00	100.00	100.00	100.00	100.00	100.00	100.00	100.00	100.00	100.00	100.00	100.00	100.00	100.00
RDP50	100.00	100.00	100.00	100.00	100.00	100.00	100.00	100.00	100.00	100.00	100.00	100.00	100.00	100.00
RDP80	100.00	100.00	100.00	100.00	100.00	100.00	100.00	100.00	100.00	100.00	100.00	100.00	100.00	100.00
SINTAX50	100.00	100.00	100.00	100.00	100.00	100.00	100.00	100.00	100.00	100.00	100.00	100.00	100.00	100.00
SINTAX80	100.00	100.00	100.00	100.00	100.00	100.00	100.00	100.00	100.00	100.00	100.00	100.00	100.00	100.00
TOP	100.00	100.00	100.00	100.00	100.00	100.00	100.00	100.00	100.00	100.00	100.00	100.00	100.00	100.00
Q1	99.84	99.91	99.84	74.97	99.92	99.84	75.00	100.00	99.84	74.94	99.84	99.68	74.94	99.84
BLCA	95.89	92.69	95.89	72.07	97.87	95.89	75.00	100.00	95.89	69.51	95.89	92.10	69.51	95.89
SPINGO	0.00	0.00	0.00	0.00	0.00	0.00	0.00	0.00	0.00	0.00	0.00	0.00	0.00	0.00

Table S70. Machine learning metrics computed for the dataset SP RDP ITS 95 at the class level.

Method	Accuracy	Balanced Accuracy	F1-score Micro	F1-score Macro	F1-score Weighted	Precision Micro	Precision Macro	Precision Weighted	Recall Micro	Recall Macro	Recall Weighted	Jaccard Micro	Jaccard Macro	Jaccard Weighted
CT2	99.68	99.12	99.68	98.65	99.69	99.68	98.45	99.71	99.68	99.12	99.68	99.37	97.57	99.39
HiTaC	99.68	99.12	99.68	98.65	99.69	99.68	98.45	99.71	99.68	99.12	99.68	99.37	97.57	99.39
HiTaC_Filter	99.68	99.12	99.68	98.65	99.69	99.68	98.45	99.71	99.68	99.12	99.68	99.37	97.57	99.39
Q2_BLAST	99.68	99.12	99.68	98.65	99.69	99.68	98.45	99.71	99.68	99.12	99.68	99.37	97.57	99.39
Q2_VS	99.68	99.12	99.68	98.65	99.69	99.68	98.45	99.71	99.68	99.12	99.68	99.37	97.57	99.39
CT1	99.60	99.00	99.60	98.58	99.61	99.60	98.42	99.63	99.60	99.00	99.60	99.21	97.42	99.23
KTOP	99.60	99.00	99.60	98.57	99.61	99.60	98.41	99.63	99.60	99.00	99.60	99.21	97.40	99.23
TOP	99.60	99.00	99.60	98.57	99.61	99.60	98.41	99.63	99.60	99.00	99.60	99.21	97.40	99.23
BTOP	99.53	98.88	99.53	98.48	99.53	99.53	98.36	99.55	99.53	98.88	99.53	99.06	97.24	99.08
RDP50	99.53	98.88	99.53	98.48	99.53	99.53	98.36	99.55	99.53	98.88	99.53	99.06	97.24	99.08
SINTAX50	99.53	98.88	99.53	93.04	99.57	99.53	92.94	99.63	99.53	93.38	99.53	99.06	91.88	99.15
Microclass	99.45	98.76	99.45	98.40	99.45	99.45	98.31	99.47	99.45	98.76	99.45	98.90	97.07	98.92
Q2_SK	99.45	98.76	99.45	92.98	99.52	99.45	92.94	99.63	99.45	93.27	99.45	98.90	91.77	99.07
RDP80	99.45	98.76	99.45	92.98	99.52	99.45	92.94	99.63	99.45	93.27	99.45	98.90	91.77	99.07
SINTAX80	99.45	98.76	99.45	92.98	99.52	99.45	92.94	99.63	99.45	93.27	99.45	98.90	91.77	99.07
NBC50	99.45	98.76	99.45	92.95	99.49	99.45	92.89	99.55	99.45	93.27	99.45	98.90	91.72	99.00
Q1	99.37	98.79	99.37	92.97	99.45	99.37	92.89	99.55	99.37	93.30	99.37	98.74	91.75	98.92
NBC80	99.37	98.63	99.37	92.92	99.48	99.37	92.94	99.63	99.37	93.16	99.37	98.74	91.65	99.00
Metaxa2	99.37	98.63	99.37	92.89	99.44	99.37	92.89	99.55	99.37	93.16	99.37	98.74	91.60	98.92
KNN	98.74	94.95	98.74	91.00	99.17	98.74	92.52	99.69	98.74	89.68	98.74	97.50	88.68	98.46
BLCA	95.42	93.36	95.42	90.08	97.21	95.42	92.41	99.49	95.42	88.17	95.42	91.23	87.10	95.02
SPINGO	0.00	0.00	0.00	0.00	0.00	0.00	0.00	0.00	0.00	0.00	0.00	0.00	0.00	0.00

Table S71. Machine learning metrics computed for the dataset SP RDP ITS 95 at the order level.

Method	Accuracy	Balanced Accuracy	F1-score Micro	F1-score Macro	F1-score Weighted	Precision Micro	Precision Macro	Precision Weighted	Recall Micro	Recall Macro	Recall Weighted	Jaccard Micro	Jaccard Macro	Jaccard Weighted
HiTaC	99.13	97.59	99.13	95.78	99.17	99.13	96.49	99.30	99.13	95.75	99.13	98.28	94.13	98.44
HiTaC_Filter	99.13	97.59	99.13	94.04	99.20	99.13	94.78	99.38	99.13	93.98	99.13	98.28	92.46	98.51
Q2_VS	99.13	96.34	99.13	94.31	99.21	99.13	94.71	99.38	99.13	94.52	99.13	98.28	93.00	98.59
Q2_BLAST	99.13	96.27	99.13	94.24	99.17	99.13	94.64	99.30	99.13	94.45	99.13	98.28	92.86	98.52
KTOP	99.05	97.20	99.05	95.80	99.06	99.05	96.93	99.19	99.05	95.36	99.05	98.12	94.19	98.25
TOP	99.05	97.20	99.05	95.80	99.06	99.05	96.93	99.19	99.05	95.36	99.05	98.12	94.19	98.25
CT2	99.05	95.35	99.05	93.64	99.12	99.05	94.67	99.30	99.05	93.55	99.05	98.12	91.99	98.44
SINTAX50	98.97	97.12	98.97	94.02	99.10	98.97	95.20	99.34	98.97	93.52	98.97	97.97	92.43	98.32
RDP50	98.97	97.00	98.97	95.49	98.97	98.97	96.62	99.13	98.97	95.17	98.97	97.97	93.68	98.11
CT1	98.89	97.21	98.89	95.00	98.92	98.89	95.66	99.08	98.89	95.38	98.89	97.81	92.93	97.99
BTOP	98.89	96.93	98.89	95.44	98.89	98.89	96.58	99.05	98.89	95.10	98.89	97.81	93.58	97.96
Microclass	98.89	96.93	98.89	95.31	98.89	98.89	96.40	99.09	98.89	95.10	98.89	97.81	93.38	97.98
RDP80	98.89	96.93	98.89	93.87	99.00	98.89	95.17	99.27	98.89	93.34	98.89	97.81	92.21	98.17
NBC50	98.89	96.93	98.89	93.68	98.92	98.89	94.83	99.13	98.89	93.34	98.89	97.81	91.87	98.03
Q2_SK	98.81	96.85	98.81	93.81	98.92	98.81	95.14	99.20	98.81	93.26	98.81	97.66	92.11	98.02
Metaxa2	98.74	96.77	98.74	93.50	98.85	98.74	94.68	99.16	98.74	93.19	98.74	97.50	91.58	97.90
Q1	98.66	96.87	98.66	93.35	98.74	98.66	94.30	99.02	98.66	93.28	98.66	97.35	91.29	97.68
NBC80	98.66	96.74	98.66	93.79	98.91	98.66	95.20	99.34	98.66	93.16	98.66	97.35	92.07	98.00
SINTAX80	98.42	96.47	98.42	93.72	98.90	98.42	95.34	99.57	98.42	92.89	98.42	96.89	91.94	97.99
BLCA	94.78	90.84	94.78	88.81	96.50	94.78	91.17	98.98	94.78	87.47	94.78	90.08	86.08	94.10
KNN	94.78	75.55	94.78	76.32	96.07	94.78	82.04	98.38	94.78	74.13	94.78	90.08	73.15	94.36
SPINGO	0.00	0.00	0.00	0.00	0.00	0.00	0.00	0.00	0.00	0.00	0.00	0.00	0.00	0.00

Table S72. Machine learning metrics computed for the dataset SP RDP ITS 95 at the family level.

Method	Accuracy	Balanced Accuracy	F1-score Micro	F1-score Macro	F1-score Weighted	Precision Micro	Precision Macro	Precision Weighted	Recall Micro	Recall Macro	Recall Weighted	Jaccard Micro	Jaccard Macro	Jaccard Weighted
HiTaC	98.02	96.05	98.02	94.06	98.03	98.02	94.26	98.37	98.02	94.76	98.02	96.12	92.48	96.74
TOP	97.79	96.39	97.79	93.45	97.91	97.79	93.92	98.44	97.79	93.84	97.79	95.67	91.89	96.56
BTOP	97.79	96.33	97.79	93.35	97.89	97.79	94.00	98.56	97.79	93.78	97.79	95.67	91.75	96.52
KTOP	97.79	96.10	97.79	92.59	97.92	97.79	93.32	98.57	97.79	92.94	97.79	95.67	90.92	96.57
RDP50	97.63	95.81	97.63	92.73	97.64	97.63	93.22	98.21	97.63	93.27	97.63	95.37	91.17	96.28
Microclass	97.55	95.82	97.55	92.86	97.67	97.55	93.45	98.26	97.55	93.28	97.55	95.22	91.02	96.21
HiTaC_Filter	97.55	94.83	97.55	93.82	98.05	97.55	94.81	98.94	97.55	93.56	97.55	95.22	92.40	96.81
SINTAX50	97.31	95.75	97.31	93.28	97.74	97.31	94.44	98.74	97.31	93.21	97.31	94.77	91.68	96.28
NBC50	97.31	95.36	97.31	91.89	97.50	97.31	92.63	98.26	97.31	92.23	97.31	94.77	90.18	96.03
Q2_SK	97.23	95.26	97.23	92.74	97.55	97.23	93.75	98.44	97.23	92.74	97.23	94.62	91.19	96.15
Q1	97.15	94.13	97.15	91.42	97.25	97.15	92.48	97.98	97.15	91.64	97.15	94.47	89.47	95.51
CT1	97.00	92.06	97.00	89.15	96.81	97.00	89.34	97.17	97.00	90.22	97.00	94.17	86.98	95.06
SPINGO	96.84	95.05	96.84	92.54	97.44	96.84	93.63	98.66	96.84	92.53	96.84	93.87	90.86	95.96
RDP80	96.84	94.92	96.84	93.07	97.43	96.84	94.23	98.65	96.84	93.03	96.84	93.87	91.36	95.97
NBC80	96.60	94.80	96.60	93.06	97.32	96.60	94.32	98.69	96.60	92.91	96.60	93.43	91.28	95.74
Metaxa2	96.28	91.68	96.28	89.61	96.69	96.28	90.60	97.87	96.28	89.84	96.28	92.84	87.79	94.98
SINTAX80	96.05	93.22	96.05	92.44	97.02	96.05	93.97	98.66	96.05	91.97	96.05	92.40	90.69	95.39
Q2_VS	95.73	87.19	95.73	85.93	95.95	95.73	86.73	96.83	95.73	86.60	95.73	91.81	83.74	94.17
CT2	95.65	84.34	95.65	82.79	95.80	95.65	83.82	96.60	95.65	83.20	95.65	91.67	80.74	94.05
Q2_BLAST	95.49	86.87	95.49	85.42	95.76	95.49	86.44	96.73	95.49	86.28	95.49	91.38	83.05	93.90
BLCA	92.96	90.80	92.96	88.47	94.77	92.96	90.51	97.92	92.96	87.81	92.96	86.85	86.27	91.80
KNN	85.14	59.25	85.14	61.04	87.49	85.14	65.84	91.92	85.14	58.85	85.14	74.12	58.15	84.56

Table S73. Machine learning metrics computed for the dataset SP RDP ITS 95 at the genus level.

Method	Accuracy	Balanced Accuracy	F1-score Micro	F1-score Macro	F1-score Weighted	Precision Micro	Precision Macro	Precision Weighted	Recall Micro	Recall Macro	Recall Weighted	Jaccard Micro	Jaccard Macro	Jaccard Weighted
HiTaC	83.64	76.31	83.64	66.46	82.44	83.64	66.64	83.47	83.64	68.60	83.64	71.88	64.26	78.77
Microclass	81.98	77.03	81.98	65.65	82.00	81.98	66.54	84.57	81.98	67.33	81.98	69.46	63.35	77.82
TOP	81.90	77.14	81.90	66.36	82.01	81.90	67.22	84.51	81.90	67.77	81.90	69.34	64.26	78.03
BTOP	81.34	76.49	81.34	65.20	81.90	81.34	66.72	85.37	81.34	66.52	81.34	68.55	62.74	77.37
NBC50	81.11	76.34	81.11	66.84	81.36	81.11	67.76	84.16	81.11	68.27	81.11	68.22	64.62	77.33
RDP50	80.87	75.77	80.87	66.39	81.24	80.87	67.35	84.26	80.87	67.94	80.87	67.88	64.12	77.18
KTOP	80.79	75.66	80.79	64.24	81.04	80.79	65.42	84.18	80.79	65.80	80.79	67.77	61.95	76.96
Q1	80.71	72.72	80.71	61.90	80.29	80.71	62.53	82.14	80.71	63.40	80.71	67.66	59.61	76.35
Q2_SK	79.92	75.06	79.92	66.21	80.74	79.92	67.46	84.49	79.92	67.30	79.92	66.56	64.10	76.69
CT1	79.68	71.08	79.68	60.03	79.56	79.68	60.70	81.82	79.68	61.67	79.68	66.23	57.79	75.70
SINTAX50	79.60	73.91	79.60	66.19	80.28	79.60	67.59	83.46	79.60	67.14	79.60	66.12	63.93	76.43
HiTaC_Filter	79.60	71.69	79.60	66.33	79.95	79.60	67.35	82.59	79.60	67.23	79.60	66.12	64.22	76.28
SPINGO	77.00	71.48	77.00	64.78	78.61	77.00	66.65	83.22	77.00	65.10	77.00	62.60	62.49	74.39
RDP80	76.60	72.35	76.60	66.35	78.14	76.60	68.40	82.46	76.60	66.59	76.60	62.08	64.01	73.95
NBC80	76.05	71.77	76.05	65.86	77.78	76.05	67.98	82.32	76.05	66.06	76.05	61.35	63.48	73.54
Metaxa2	75.81	68.64	75.81	61.53	77.38	75.81	63.13	81.97	75.81	62.35	75.81	61.04	59.23	72.80
BLCA	75.18	70.18	75.18	62.12	76.89	75.18	63.98	81.71	75.18	62.60	75.18	60.23	59.90	72.56
Q2_VS	72.65	57.03	72.65	52.13	72.15	72.65	53.08	74.63	72.65	53.78	72.65	57.05	49.89	68.29
Q2_BLAST	72.41	55.99	72.41	50.68	71.64	72.41	50.81	72.62	72.41	52.51	72.41	56.75	48.54	67.81
CT2	71.15	51.83	71.15	47.39	70.43	71.15	47.66	72.26	71.15	49.14	71.15	55.21	45.39	66.76
SINTAX80	70.91	64.77	70.91	62.10	73.62	70.91	64.97	79.61	70.91	61.41	70.91	54.93	59.73	69.22
KNN	46.01	24.46	46.01	25.16	49.58	46.01	28.20	58.94	46.01	24.25	46.01	29.88	23.56	45.31

Table S74. Machine learning metrics computed for the dataset SP RDP ITS 95 at the species level.

Method	Accuracy	Balanced Accuracy	F1-score Micro	F1-score Macro	F1-score Weighted	Precision Micro	Precision Macro	Precision Weighted	Recall Micro	Recall Macro	Recall Weighted	Jaccard Micro	Jaccard Macro	Jaccard Weighted
BTOP	0.32	0.38	0.32	0.18	0.26	0.32	0.16	0.24	0.32	0.21	0.32	0.16	0.16	0.22
KTOP	0.24	0.32	0.24	0.16	0.21	0.24	0.15	0.20	0.24	0.18	0.24	0.12	0.15	0.20
HiTaC	0.24	0.32	0.24	0.14	0.18	0.24	0.13	0.17	0.24	0.18	0.24	0.12	0.13	0.17
Microclass	0.24	0.32	0.24	0.14	0.18	0.24	0.13	0.17	0.24	0.18	0.24	0.12	0.13	0.17
HiTaC_Filter	0.16	0.22	0.16	0.18	0.16	0.16	0.18	0.16	0.16	0.18	0.16	0.08	0.18	0.16
SINTAX50	0.16	0.22	0.16	0.15	0.16	0.16	0.15	0.16	0.16	0.15	0.16	0.08	0.15	0.16
Q2_SK	0.16	0.22	0.16	0.14	0.16	0.16	0.14	0.16	0.16	0.14	0.16	0.08	0.14	0.16
TOP	0.16	0.22	0.16	0.12	0.16	0.16	0.12	0.16	0.16	0.12	0.16	0.08	0.12	0.16
NBC50	0.16	0.22	0.16	0.11	0.13	0.16	0.10	0.12	0.16	0.13	0.16	0.08	0.10	0.12
RDP50	0.16	0.22	0.16	0.11	0.13	0.16	0.10	0.12	0.16	0.13	0.16	0.08	0.10	0.12
Q2_BLAST	0.08	0.11	0.08	0.10	0.08	0.08	0.10	0.08	0.08	0.10	0.08	0.04	0.10	0.08
Q2_VS	0.08	0.11	0.08	0.10	0.08	0.08	0.10	0.08	0.08	0.10	0.08	0.04	0.10	0.08
SINTAX80	0.08	0.11	0.08	0.09	0.08	0.08	0.09	0.08	0.08	0.09	0.08	0.04	0.09	0.08
NBC80	0.08	0.11	0.08	0.08	0.08	0.08	0.08	0.08	0.08	0.08	0.08	0.04	0.08	0.08
RDP80	0.08	0.11	0.08	0.08	0.08	0.08	0.08	0.08	0.08	0.08	0.08	0.04	0.08	0.08
SPINGO	0.08	0.11	0.08	0.08	0.08	0.08	0.08	0.08	0.08	0.08	0.08	0.04	0.08	0.08
BLCA	0.08	0.11	0.08	0.07	0.08	0.08	0.07	0.08	0.08	0.07	0.08	0.04	0.07	0.08
Q1	0.08	0.11	0.08	0.07	0.08	0.08	0.07	0.08	0.08	0.07	0.08	0.04	0.07	0.08
CT1	0.00	0.00	0.00	0.00	0.00	0.00	0.00	0.00	0.00	0.00	0.00	0.00	0.00	0.00
CT2	0.00	0.00	0.00	0.00	0.00	0.00	0.00	0.00	0.00	0.00	0.00	0.00	0.00	0.00
KNN	0.00	0.00	0.00	0.00	0.00	0.00	0.00	0.00	0.00	0.00	0.00	0.00	0.00	0.00
Metaxa2	0.00	0.00	0.00	0.00	0.00	0.00	0.00	0.00	0.00	0.00	0.00	0.00	0.00	0.00

Table S75. Machine learning metrics computed for the dataset SP RDP ITS 97 at the phylum level.

Method	Accuracy	Balanced Accuracy	F1-score Micro	F1-score Macro	F1-score Weighted	Precision Micro	Precision Macro	Precision Weighted	Recall Micro	Recall Macro	Recall Weighted	Jaccard Micro	Jaccard Macro	Jaccard Weighted
BTOP	100.00	100.00	100.00	100.00	100.00	100.00	100.00	100.00	100.00	100.00	100.00	100.00	100.00	100.00
CT1	100.00	100.00	100.00	100.00	100.00	100.00	100.00	100.00	100.00	100.00	100.00	100.00	100.00	100.00
CT2	100.00	100.00	100.00	100.00	100.00	100.00	100.00	100.00	100.00	100.00	100.00	100.00	100.00	100.00
HiTaC	100.00	100.00	100.00	100.00	100.00	100.00	100.00	100.00	100.00	100.00	100.00	100.00	100.00	100.00
HiTaC_Filter	100.00	100.00	100.00	100.00	100.00	100.00	100.00	100.00	100.00	100.00	100.00	100.00	100.00	100.00
KTOP	100.00	100.00	100.00	100.00	100.00	100.00	100.00	100.00	100.00	100.00	100.00	100.00	100.00	100.00
Microclass	100.00	100.00	100.00	100.00	100.00	100.00	100.00	100.00	100.00	100.00	100.00	100.00	100.00	100.00
NBC50	100.00	100.00	100.00	100.00	100.00	100.00	100.00	100.00	100.00	100.00	100.00	100.00	100.00	100.00
NBC80	100.00	100.00	100.00	100.00	100.00	100.00	100.00	100.00	100.00	100.00	100.00	100.00	100.00	100.00
Q2_BLAST	100.00	100.00	100.00	100.00	100.00	100.00	100.00	100.00	100.00	100.00	100.00	100.00	100.00	100.00
Q2_SK	100.00	100.00	100.00	100.00	100.00	100.00	100.00	100.00	100.00	100.00	100.00	100.00	100.00	100.00
RDP50	100.00	100.00	100.00	100.00	100.00	100.00	100.00	100.00	100.00	100.00	100.00	100.00	100.00	100.00
RDP80	100.00	100.00	100.00	100.00	100.00	100.00	100.00	100.00	100.00	100.00	100.00	100.00	100.00	100.00
SINTAX50	100.00	100.00	100.00	100.00	100.00	100.00	100.00	100.00	100.00	100.00	100.00	100.00	100.00	100.00
SINTAX80	100.00	100.00	100.00	100.00	100.00	100.00	100.00	100.00	100.00	100.00	100.00	100.00	100.00	100.00
TOP	100.00	100.00	100.00	100.00	100.00	100.00	100.00	100.00	100.00	100.00	100.00	100.00	100.00	100.00
Q2_VS	99.93	99.98	99.93	85.71	99.96	99.93	85.71	100.00	99.93	85.70	99.93	99.86	85.70	99.93
Q1	99.93	99.96	99.93	85.70	99.96	99.93	85.71	100.00	99.93	85.68	99.93	99.86	85.68	99.93
KNN	99.86	66.67	99.86	57.14	99.86	99.86	57.14	99.86	99.86	57.14	99.86	99.72	57.14	99.86
Metaxa2	99.86	66.67	99.86	57.14	99.86	99.86	57.14	99.86	99.86	57.14	99.86	99.72	57.14	99.86
BLCA	94.51	53.31	94.51	50.01	97.07	94.51	57.14	99.86	94.51	45.69	94.51	89.59	45.69	94.51
SPINGO	0.00	0.00	0.00	0.00	0.00	0.00	0.00	0.00	0.00	0.00	0.00	0.00	0.00	0.00

Table S76. Machine learning metrics computed for the dataset SP RDP ITS 97 at the class level.

Method	Accuracy	Balanced Accuracy	F1-score Micro	F1-score Macro	F1-score Weighted	Precision Micro	Precision Macro	Precision Weighted	Recall Micro	Recall Macro	Recall Weighted	Jaccard Micro	Jaccard Macro	Jaccard Weighted
BTOP	99.93	99.96	99.93	99.94	99.93	99.93	99.92	99.93	99.93	99.96	99.93	99.86	99.88	99.86
CT2	99.93	99.96	99.93	99.94	99.93	99.93	99.92	99.93	99.93	99.96	99.93	99.86	99.88	99.86
HiTaC	99.93	99.96	99.93	99.94	99.93	99.93	99.92	99.93	99.93	99.96	99.93	99.86	99.88	99.86
HiTaC_Filter	99.93	99.96	99.93	99.94	99.93	99.93	99.92	99.93	99.93	99.96	99.93	99.86	99.88	99.86
KTOP	99.93	99.96	99.93	99.94	99.93	99.93	99.92	99.93	99.93	99.96	99.93	99.86	99.88	99.86
Microclass	99.93	99.96	99.93	99.94	99.93	99.93	99.92	99.93	99.93	99.96	99.93	99.86	99.88	99.86
NBC50	99.93	99.96	99.93	99.94	99.93	99.93	99.92	99.93	99.93	99.96	99.93	99.86	99.88	99.86
NBC80	99.93	99.96	99.93	99.94	99.93	99.93	99.92	99.93	99.93	99.96	99.93	99.86	99.88	99.86
Q2_SK	99.93	99.96	99.93	99.94	99.93	99.93	99.92	99.93	99.93	99.96	99.93	99.86	99.88	99.86
RDP50	99.93	99.96	99.93	99.94	99.93	99.93	99.92	99.93	99.93	99.96	99.93	99.86	99.88	99.86
RDP80	99.93	99.96	99.93	99.94	99.93	99.93	99.92	99.93	99.93	99.96	99.93	99.86	99.88	99.86
SINTAX50	99.93	99.96	99.93	99.94	99.93	99.93	99.92	99.93	99.93	99.96	99.93	99.86	99.88	99.86
TOP	99.93	99.96	99.93	99.94	99.93	99.93	99.92	99.93	99.93	99.96	99.93	99.86	99.88	99.86
Q2_BLAST	99.86	99.88	99.86	99.88	99.86	99.86	99.89	99.86	99.86	99.88	99.86	99.72	99.77	99.72
CT1	99.86	99.88	99.86	94.90	99.89	99.86	94.92	99.93	99.86	94.88	99.86	99.72	94.81	99.79
Q2_VS	99.86	99.88	99.86	94.90	99.89	99.86	94.92	99.93	99.86	94.88	99.86	99.72	94.81	99.79
SINTAX80	99.86	99.88	99.86	94.90	99.89	99.86	94.92	99.93	99.86	94.88	99.86	99.72	94.81	99.79
Q1	99.79	99.86	99.79	94.90	99.86	99.79	94.92	99.93	99.79	94.87	99.79	99.58	94.79	99.72
Metaxa2	99.72	89.35	99.72	84.90	99.75	99.72	84.92	99.79	99.72	84.88	99.72	99.44	84.81	99.65
KNN	98.10	82.41	98.10	80.98	98.88	98.10	84.91	99.78	98.10	78.29	98.10	96.27	78.22	98.04
BLCA	94.44	81.54	94.44	80.43	96.73	94.44	84.92	99.79	94.44	77.46	94.44	89.46	77.39	94.37
SPINGO	0.00	0.00	0.00	0.00	0.00	0.00	0.00	0.00	0.00	0.00	0.00	0.00	0.00	0.00

Table S77. Machine learning metrics computed for the dataset SP RDP ITS 97 at the order level.

Method	Accuracy	Balanced Accuracy	F1-score Micro	F1-score Macro	F1-score Weighted	Precision Micro	Precision Macro	Precision Weighted	Recall Micro	Recall Macro	Recall Weighted	Jaccard Micro	Jaccard Macro	Jaccard Weighted
BTOP	99.44	98.34	99.44	97.67	99.51	99.44	98.25	99.69	99.44	98.34	99.44	98.88	96.59	99.12
KTOP	99.44	98.34	99.44	97.67	99.51	99.44	98.25	99.69	99.44	98.34	99.44	98.88	96.59	99.12
Microclass	99.44	98.34	99.44	97.67	99.51	99.44	98.25	99.69	99.44	98.34	99.44	98.88	96.59	99.12
NBC50	99.44	98.34	99.44	97.67	99.51	99.44	98.25	99.69	99.44	98.34	99.44	98.88	96.59	99.12
NBC80	99.44	98.34	99.44	97.67	99.51	99.44	98.25	99.69	99.44	98.34	99.44	98.88	96.59	99.12
Q2_SK	99.44	98.34	99.44	97.67	99.51	99.44	98.25	99.69	99.44	98.34	99.44	98.88	96.59	99.12
RDP50	99.44	98.34	99.44	97.67	99.51	99.44	98.25	99.69	99.44	98.34	99.44	98.88	96.59	99.12
RDP80	99.44	98.34	99.44	97.67	99.51	99.44	98.25	99.69	99.44	98.34	99.44	98.88	96.59	99.12
TOP	99.44	98.34	99.44	97.67	99.51	99.44	98.25	99.69	99.44	98.34	99.44	98.88	96.59	99.12
SINTAX50	99.37	98.27	99.37	96.11	99.48	99.37	96.71	99.69	99.37	96.74	99.37	98.74	95.01	99.05
HiTaC	99.37	98.14	99.37	97.34	99.44	99.37	97.85	99.63	99.37	98.14	99.37	98.74	95.99	99.00
HiTaC_Filter	99.37	98.14	99.37	96.04	99.47	99.37	96.71	99.69	99.37	96.61	99.37	98.74	94.88	99.05
SINTAX80	99.30	98.16	99.30	96.08	99.47	99.30	96.75	99.75	99.30	96.63	99.30	98.60	94.94	99.05
Q1	99.30	98.14	99.30	95.84	99.44	99.30	96.36	99.70	99.30	96.60	99.30	98.60	94.53	99.00
Q2_VS	99.30	96.71	99.30	94.37	99.44	99.30	94.80	99.70	99.30	95.20	99.30	98.60	93.13	99.07
Q2_BLAST	99.30	96.71	99.30	94.36	99.41	99.30	94.79	99.63	99.30	95.20	99.30	98.60	93.11	99.00
CT1	99.23	97.98	99.23	95.69	99.34	99.23	96.23	99.56	99.23	96.45	99.23	98.46	94.24	98.79
CT2	99.23	96.62	99.23	94.25	99.34	99.23	94.68	99.56	99.23	95.11	99.23	98.46	92.91	98.86
Metaxa2	99.01	94.72	99.01	92.81	99.26	99.01	93.62	99.61	99.01	93.24	99.01	98.05	91.55	98.77
KNN	95.85	78.05	95.85	76.82	97.09	95.85	78.00	98.55	95.85	76.83	95.85	92.02	75.15	95.67
BLCA	93.87	90.37	93.87	89.59	96.14	93.87	92.02	99.48	93.87	88.96	93.87	88.45	87.23	93.56
SPINGO	0.00	0.00	0.00	0.00	0.00	0.00	0.00	0.00	0.00	0.00	0.00	0.00	0.00	0.00

Table S78. Machine learning metrics computed for the dataset SP RDP ITS 97 at the family level.

Method	Accuracy	Balanced Accuracy	F1-score Micro	F1-score Macro	F1-score Weighted	Precision Micro	Precision Macro	Precision Weighted	Recall Micro	Recall Macro	Recall Weighted	Jaccard Micro	Jaccard Macro	Jaccard Weighted
TOP	99.23	98.28	99.23	97.07	99.26	99.23	97.40	99.55	99.23	97.65	99.23	98.46	96.33	98.85
BTOP	99.16	97.95	99.16	96.34	99.21	99.16	96.78	99.52	99.16	96.71	99.16	98.33	95.61	98.79
KTOP	99.15	98.25	99.15	97.02	99.19	99.15	97.33	99.49	99.15	97.62	99.15	98.32	96.24	98.71
Microclass	99.08	98.23	99.08	96.98	99.12	99.08	97.28	99.44	99.08	97.60	99.08	98.19	96.16	98.59
NBC50	99.08	98.23	99.08	96.98	99.12	99.08	97.28	99.44	99.08	97.60	99.08	98.19	96.16	98.59
Q2_SK	99.08	98.23	99.08	96.98	99.12	99.08	97.28	99.44	99.08	97.60	99.08	98.19	96.16	98.59
RDP50	99.08	98.23	99.08	96.98	99.12	99.08	97.28	99.44	99.08	97.60	99.08	98.19	96.16	98.59
RDP80	99.08	98.23	99.08	96.42	99.19	99.08	96.78	99.55	99.08	96.98	99.08	98.19	95.67	98.71
NBC80	99.01	98.20	99.01	96.62	99.17	99.01	97.09	99.58	99.01	96.95	99.01	98.05	95.96	98.67
SINTAX50	98.94	98.09	98.94	96.35	99.11	98.94	96.78	99.55	98.94	96.84	98.94	97.91	95.53	98.56
HiTaC	98.94	97.96	98.94	96.11	99.02	98.94	96.55	99.43	98.94	96.71	98.94	97.91	95.17	98.45
HiTaC_Filter	98.87	97.93	98.87	95.62	99.05	98.87	96.16	99.55	98.87	96.07	98.87	97.77	94.77	98.49
SPINGO	98.73	97.79	98.73	96.15	98.99	98.73	96.77	99.54	98.73	96.54	98.73	97.50	95.23	98.36
CT1	98.66	95.24	98.66	93.44	98.66	98.66	93.84	98.95	98.66	94.03	98.66	97.36	92.36	97.98
Q1	98.45	97.76	98.45	95.97	98.60	98.45	96.44	99.09	98.45	96.52	98.45	96.95	94.88	97.64
SINTAX80	98.31	96.58	98.31	95.75	98.67	98.31	96.76	99.48	98.31	95.96	98.31	96.68	94.64	97.93
Metaxa2	98.10	93.36	98.10	92.23	98.47	98.10	93.27	99.16	98.10	92.17	98.10	96.27	91.18	97.76
Q2_VS	97.39	93.75	97.39	91.87	97.63	97.39	93.12	98.43	97.39	91.97	97.39	94.92	90.16	96.19
Q2_BLAST	97.39	93.70	97.39	90.85	97.66	97.39	91.68	98.45	97.39	91.34	97.39	94.92	89.31	96.40
CT2	96.41	89.39	96.41	88.18	96.59	96.41	90.06	97.68	96.41	88.25	96.41	93.07	85.96	94.80
BLCA	93.38	90.80	93.38	89.55	95.45	93.38	91.10	98.74	93.38	89.08	93.38	87.58	87.99	93.02
KNN	85.63	64.32	85.63	66.23	88.40	85.63	71.56	93.77	85.63	63.90	85.63	74.88	63.78	85.46

Table S79. Machine learning metrics computed for the dataset SP RDP ITS 97 at the genus level.

Method	Accuracy	Balanced Accuracy	F1-score Micro	F1-score Macro	F1-score Weighted	Precision Micro	Precision Macro	Precision Weighted	Recall Micro	Recall Macro	Recall Weighted	Jaccard Micro	Jaccard Macro	Jaccard Weighted
HiTaC	91.55	89.31	91.55	84.36	90.79	91.55	85.15	91.67	91.55	85.07	91.55	84.42	82.67	87.69
Microclass	91.06	89.41	91.06	84.24	90.74	91.06	85.25	91.99	91.06	84.72	91.06	83.58	82.49	87.23
TOP	90.99	89.33	90.99	84.40	90.42	90.99	85.49	91.58	90.99	84.86	90.99	83.46	82.70	87.12
KTOP	90.99	89.33	90.99	83.85	90.46	90.99	84.79	91.49	90.99	84.42	90.99	83.46	82.09	87.07
BTOP	90.85	89.60	90.85	84.53	90.38	90.85	85.55	91.38	90.85	84.90	90.85	83.24	82.87	86.93
NBC50	90.56	88.93	90.56	83.93	90.21	90.56	84.75	91.37	90.56	84.48	90.56	82.75	82.24	86.91
RDP50	90.49	88.82	90.49	84.21	90.25	90.49	85.12	91.41	90.49	84.60	90.49	82.64	82.49	86.89
Q2_SK	89.08	88.05	89.08	83.73	89.23	89.08	84.78	91.09	89.08	84.09	89.08	80.32	82.03	85.77
SINTAX50	88.94	86.85	88.94	83.12	89.01	88.94	84.69	91.02	88.94	83.17	88.94	80.09	81.30	85.53
CT1	87.89	80.70	87.89	73.99	87.24	87.89	74.78	88.41	87.89	74.70	87.89	78.39	72.07	83.55
HiTaC_Filter	87.68	85.02	87.68	82.36	88.63	87.68	84.13	91.30	87.68	82.07	87.68	78.06	80.66	85.16
Q1	87.18	82.75	87.18	75.23	86.91	87.18	76.43	88.63	87.18	75.63	87.18	77.28	73.06	82.73
SPINGO	86.48	84.67	86.48	82.03	87.85	86.48	84.56	91.69	86.48	81.50	86.48	76.18	80.10	84.16
NBC80	86.41	85.42	86.41	82.63	87.60	86.41	84.76	91.15	86.41	82.24	86.41	76.07	80.96	84.13
RDP80	86.34	84.90	86.34	82.29	87.50	86.34	84.33	91.00	86.34	81.95	86.34	75.96	80.57	84.07
Metaxa2	84.93	78.12	84.93	74.57	85.65	84.93	76.62	88.17	84.93	74.22	84.93	73.81	72.62	81.69
BLCA	82.96	79.28	82.96	75.61	84.27	82.96	78.68	88.81	82.96	74.92	82.96	70.88	73.47	80.50
SINTAX80	82.25	78.99	82.25	77.74	83.93	82.25	80.18	88.36	82.25	77.07	82.25	69.86	76.00	80.48
Q2_BLAST	78.03	65.61	78.03	61.17	77.67	78.03	62.09	79.97	78.03	62.17	78.03	63.97	58.94	73.27
Q2_VS	77.89	65.86	77.89	62.48	77.83	77.89	64.02	80.80	77.89	63.06	77.89	63.78	60.26	73.33
CT2	75.99	61.01	75.99	56.97	75.40	75.99	58.02	78.05	75.99	57.96	75.99	61.27	54.94	71.13
KNN	48.03	30.70	48.03	31.76	51.49	48.03	34.66	59.27	48.03	30.62	48.03	31.60	30.18	47.16

Table S80. Machine learning metrics computed for the dataset SP RDP ITS 97 at the species level.

Method	Accuracy	Balanced Accuracy	F1-score Micro	F1-score Macro	F1-score Weighted	Precision Micro	Precision Macro	Precision Weighted	Recall Micro	Recall Macro	Recall Weighted	Jaccard Micro	Jaccard Macro	Jaccard Weighted
TOP	3.66	4.94	3.66	2.75	3.54	3.66	2.71	3.50	3.66	2.84	3.66	1.87	2.71	3.50
HiTaC	3.66	4.89	3.66	2.75	3.60	3.66	2.73	3.58	3.66	2.80	3.66	1.87	2.72	3.56
BTOP	3.66	4.84	3.66	2.66	3.50	3.66	2.62	3.44	3.66	2.78	3.66	1.86	2.62	3.44
Microclass	3.59	4.84	3.59	2.73	3.48	3.59	2.70	3.45	3.59	2.81	3.59	1.83	2.70	3.45
KTOP	3.52	4.75	3.52	2.64	3.35	3.52	2.59	3.30	3.52	2.77	3.52	1.79	2.59	3.30
Q2_SK	3.45	4.65	3.45	3.01	3.39	3.45	2.99	3.37	3.45	3.06	3.45	1.76	2.99	3.37
RDP50	3.45	4.65	3.45	2.88	3.39	3.45	2.86	3.37	3.45	2.93	3.45	1.76	2.86	3.37
NBC50	3.45	4.65	3.45	2.86	3.39	3.45	2.84	3.37	3.45	2.91	3.45	1.76	2.84	3.37
SINTAX50	3.17	4.27	3.17	2.84	3.05	3.17	2.79	3.00	3.17	2.95	3.17	1.61	2.79	3.00
NBC80	3.03	4.08	3.03	2.94	3.00	3.03	2.93	2.99	3.03	2.96	3.03	1.54	2.93	2.99
SPINGO	3.03	4.08	3.03	2.82	2.93	3.03	2.79	2.90	3.03	2.91	3.03	1.54	2.79	2.90
RDP80	2.96	3.99	2.96	2.88	2.93	2.96	2.86	2.92	2.96	2.90	2.96	1.50	2.86	2.92
HiTaC_Filter	2.89	3.89	2.89	2.92	2.84	2.89	2.90	2.82	2.89	2.97	2.89	1.46	2.90	2.82
BLCA	2.61	3.51	2.61	2.27	2.54	2.61	2.25	2.52	2.61	2.33	2.61	1.32	2.25	2.52
SINTAX80	2.39	3.23	2.39	2.60	2.37	2.39	2.58	2.36	2.39	2.62	2.39	1.21	2.58	2.36
Q1	1.69	2.28	1.69	1.40	1.62	1.69	1.37	1.58	1.69	1.46	1.69	0.85	1.37	1.58
Q2_BLAST	0.77	1.04	0.77	1.01	0.77	0.77	1.01	0.77	0.77	1.01	0.77	0.39	1.01	0.77
Q2_VS	0.70	0.95	0.70	0.92	0.70	0.70	0.92	0.70	0.70	0.92	0.70	0.35	0.92	0.70
CT1	0.56	0.76	0.56	0.43	0.48	0.56	0.40	0.45	0.56	0.50	0.56	0.28	0.40	0.45
Metaxa2	0.35	0.47	0.35	0.31	0.31	0.35	0.28	0.28	0.35	0.36	0.35	0.18	0.28	0.28
CT2	0.21	0.28	0.21	0.28	0.21	0.21	0.28	0.21	0.21	0.28	0.21	0.11	0.28	0.21
KNN	0.00	0.00	0.00	0.00	0.00	0.00	0.00	0.00	0.00	0.00	0.00	0.00	0.00	0.00

Table S81. Machine learning metrics computed for the dataset SP RDP ITS 99 at the phylum level.

Method	Accuracy	Balanced Accuracy	F1-score Micro	F1-score Macro	F1-score Weighted	Precision Micro	Precision Macro	Precision Weighted	Recall Micro	Recall Macro	Recall Weighted	Jaccard Micro	Jaccard Macro	Jaccard Weighted
BTOP	100.00	100.00	100.00	100.00	100.00	100.00	100.00	100.00	100.00	100.00	100.00	100.00	100.00	100.00
CT1	100.00	100.00	100.00	100.00	100.00	100.00	100.00	100.00	100.00	100.00	100.00	100.00	100.00	100.00
CT2	100.00	100.00	100.00	100.00	100.00	100.00	100.00	100.00	100.00	100.00	100.00	100.00	100.00	100.00
HiTaC	100.00	100.00	100.00	100.00	100.00	100.00	100.00	100.00	100.00	100.00	100.00	100.00	100.00	100.00
HiTaC_Filter	100.00	100.00	100.00	100.00	100.00	100.00	100.00	100.00	100.00	100.00	100.00	100.00	100.00	100.00
KTOP	100.00	100.00	100.00	100.00	100.00	100.00	100.00	100.00	100.00	100.00	100.00	100.00	100.00	100.00
Microclass	100.00	100.00	100.00	100.00	100.00	100.00	100.00	100.00	100.00	100.00	100.00	100.00	100.00	100.00
NBC50	100.00	100.00	100.00	100.00	100.00	100.00	100.00	100.00	100.00	100.00	100.00	100.00	100.00	100.00
NBC80	100.00	100.00	100.00	100.00	100.00	100.00	100.00	100.00	100.00	100.00	100.00	100.00	100.00	100.00
Q2_SK	100.00	100.00	100.00	100.00	100.00	100.00	100.00	100.00	100.00	100.00	100.00	100.00	100.00	100.00
RDP50	100.00	100.00	100.00	100.00	100.00	100.00	100.00	100.00	100.00	100.00	100.00	100.00	100.00	100.00
RDP80	100.00	100.00	100.00	100.00	100.00	100.00	100.00	100.00	100.00	100.00	100.00	100.00	100.00	100.00
SINTAX50	100.00	100.00	100.00	100.00	100.00	100.00	100.00	100.00	100.00	100.00	100.00	100.00	100.00	100.00
SINTAX80	100.00	100.00	100.00	100.00	100.00	100.00	100.00	100.00	100.00	100.00	100.00	100.00	100.00	100.00
TOP	100.00	100.00	100.00	100.00	100.00	100.00	100.00	100.00	100.00	100.00	100.00	100.00	100.00	100.00
Metaxa2	99.97	95.00	99.97	80.95	99.99	99.97	83.33	100.00	99.97	79.17	99.97	99.95	79.17	99.97
Q2_BLAST	99.90	99.96	99.90	83.32	99.95	99.90	83.33	100.00	99.90	83.30	99.90	99.80	83.30	99.90
Q2_VS	99.87	99.95	99.87	83.31	99.94	99.87	83.33	100.00	99.87	83.29	99.87	99.75	83.29	99.87
KNN	99.77	69.56	99.77	60.93	99.86	99.77	66.67	99.97	99.77	57.97	99.77	99.54	57.97	99.77
Q1	99.75	99.90	99.75	83.29	99.87	99.75	83.33	100.00	99.75	83.25	99.75	99.49	83.25	99.75
BLCA	95.21	89.50	95.21	78.46	97.51	95.21	83.33	100.00	95.21	74.58	95.21	90.86	74.58	95.21
SPINGO	0.00	0.00	0.00	0.00	0.00	0.00	0.00	0.00	0.00	0.00	0.00	0.00	0.00	0.00

Table S82. Machine learning metrics computed for the dataset SP RDP ITS 99 at the class level.

Method	Accuracy	Balanced Accuracy	F1-score Micro	F1-score Macro	F1-score Weighted	Precision Micro	Precision Macro	Precision Weighted	Recall Micro	Recall Macro	Recall Weighted	Jaccard Micro	Jaccard Macro	Jaccard Weighted
BTOP	100.00	100.00	100.00	100.00	100.00	100.00	100.00	100.00	100.00	100.00	100.00	100.00	100.00	100.00
HiTaC	100.00	100.00	100.00	100.00	100.00	100.00	100.00	100.00	100.00	100.00	100.00	100.00	100.00	100.00
HiTaC_Filter	100.00	100.00	100.00	100.00	100.00	100.00	100.00	100.00	100.00	100.00	100.00	100.00	100.00	100.00
KTOP	100.00	100.00	100.00	100.00	100.00	100.00	100.00	100.00	100.00	100.00	100.00	100.00	100.00	100.00
Microclass	100.00	100.00	100.00	100.00	100.00	100.00	100.00	100.00	100.00	100.00	100.00	100.00	100.00	100.00
NBC50	100.00	100.00	100.00	100.00	100.00	100.00	100.00	100.00	100.00	100.00	100.00	100.00	100.00	100.00
NBC80	100.00	100.00	100.00	100.00	100.00	100.00	100.00	100.00	100.00	100.00	100.00	100.00	100.00	100.00
Q2_SK	100.00	100.00	100.00	100.00	100.00	100.00	100.00	100.00	100.00	100.00	100.00	100.00	100.00	100.00
RDP50	100.00	100.00	100.00	100.00	100.00	100.00	100.00	100.00	100.00	100.00	100.00	100.00	100.00	100.00
RDP80	100.00	100.00	100.00	100.00	100.00	100.00	100.00	100.00	100.00	100.00	100.00	100.00	100.00	100.00
SINTAX50	100.00	100.00	100.00	100.00	100.00	100.00	100.00	100.00	100.00	100.00	100.00	100.00	100.00	100.00
TOP	100.00	100.00	100.00	100.00	100.00	100.00	100.00	100.00	100.00	100.00	100.00	100.00	100.00	100.00
SINTAX80	99.97	99.99	99.97	96.15	99.99	99.97	96.15	100.00	99.97	96.14	99.97	99.95	96.14	99.97
CT1	99.87	98.23	99.87	98.73	99.87	99.87	99.51	99.88	99.87	98.23	99.87	99.75	97.75	99.75
Q2_VS	99.70	99.50	99.70	95.74	99.76	99.70	95.83	99.82	99.70	95.67	99.70	99.39	95.35	99.52
Q1	99.70	98.91	99.70	95.48	99.82	99.70	95.87	99.95	99.70	95.10	99.70	99.39	94.89	99.65
Q2_BLAST	99.70	98.17	99.70	94.98	99.76	99.70	95.83	99.82	99.70	94.39	99.70	99.39	94.07	99.52
CT2	99.70	98.16	99.70	94.87	99.72	99.70	95.62	99.75	99.70	94.38	99.70	99.39	93.87	99.45
Metaxa2	99.65	92.82	99.65	90.52	99.80	99.65	92.31	99.97	99.65	89.25	99.65	99.29	89.25	99.65
KNN	97.85	66.37	97.85	65.80	98.75	97.85	69.23	99.75	97.85	63.82	97.85	95.78	63.82	97.85
BLCA	95.18	91.29	95.18	89.76	97.36	95.18	92.31	99.95	95.18	87.78	95.18	90.81	87.78	95.18
SPINGO	0.00	0.00	0.00	0.00	0.00	0.00	0.00	0.00	0.00	0.00	0.00	0.00	0.00	0.00

Table S83. Machine learning metrics computed for the dataset SP RDP ITS 99 at the order level.

Method	Accuracy	Balanced Accuracy	F1-score Micro	F1-score Macro	F1-score Weighted	Precision Micro	Precision Macro	Precision Weighted	Recall Micro	Recall Macro	Recall Weighted	Jaccard Micro	Jaccard Macro	Jaccard Weighted
BTOP	99.97	99.97	99.97	99.98	99.97	99.97	99.99	99.98	99.97	99.97	99.97	99.95	99.97	99.95
HiTaC	99.97	99.97	99.97	99.98	99.97	99.97	99.99	99.97	99.97	99.97	99.97	99.95	99.97	99.95
HiTaC_Filter	99.97	99.97	99.97	99.98	99.97	99.97	99.99	99.97	99.97	99.97	99.97	99.95	99.97	99.95
Microclass	99.97	99.97	99.97	99.98	99.97	99.97	99.99	99.97	99.97	99.97	99.97	99.95	99.97	99.95
KTOP	99.95	99.97	99.95	99.98	99.95	99.95	99.99	99.95	99.95	99.97	99.95	99.90	99.95	99.90
NBC50	99.95	99.97	99.95	99.98	99.95	99.95	99.99	99.95	99.95	99.97	99.95	99.90	99.95	99.90
RDP50	99.95	99.97	99.95	99.98	99.95	99.95	99.99	99.95	99.95	99.97	99.95	99.90	99.95	99.90
TOP	99.95	99.97	99.95	99.98	99.95	99.95	99.99	99.95	99.95	99.97	99.95	99.90	99.95	99.90
Q2_SK	99.95	99.97	99.95	98.82	99.96	99.95	98.83	99.97	99.95	98.80	99.95	99.90	98.80	99.92
SINTAX50	99.95	99.97	99.95	98.82	99.96	99.95	98.83	99.97	99.95	98.80	99.95	99.90	98.80	99.92
SINTAX80	99.92	99.96	99.92	98.81	99.95	99.92	98.83	99.97	99.92	98.80	99.92	99.85	98.79	99.90
NBC80	99.92	99.96	99.92	98.81	99.94	99.92	98.83	99.95	99.92	98.80	99.92	99.85	98.78	99.87
RDP80	99.92	99.96	99.92	98.81	99.94	99.92	98.83	99.95	99.92	98.80	99.92	99.85	98.78	99.87
Q1	99.39	99.22	99.39	98.31	99.54	99.39	98.58	99.70	99.39	98.07	99.39	98.79	97.83	99.10
CT1	99.29	97.29	99.29	95.31	99.33	99.29	96.30	99.43	99.29	95.06	99.29	98.59	93.68	98.73
Q2_VS	99.04	96.20	99.04	95.44	99.13	99.04	96.24	99.27	99.04	95.08	99.04	98.09	93.86	98.41
Q2_BLAST	98.91	95.71	98.91	95.15	99.02	98.91	96.22	99.19	98.91	94.60	98.91	97.84	93.35	98.21
Metaxa2	98.66	87.63	98.66	88.26	99.14	98.66	90.69	99.70	98.66	86.61	98.66	97.35	86.61	98.63
CT2	98.45	89.42	98.45	88.31	98.59	98.45	91.04	98.87	98.45	87.37	98.45	96.95	85.54	97.58
BLCA	95.16	94.55	95.16	95.16	97.20	95.16	97.67	99.91	95.16	93.46	95.16	90.76	93.45	95.14
KNN	93.16	56.50	93.16	58.16	95.37	93.16	61.62	97.97	93.16	55.84	93.16	87.19	55.83	93.13
SPINGO	0.00	0.00	0.00	0.00	0.00	0.00	0.00	0.00	0.00	0.00	0.00	0.00	0.00	0.00

Table S84. Machine learning metrics computed for the dataset SP RDP ITS 99 at the family level.

Method	Accuracy	Balanced Accuracy	F1-score Micro	F1-score Macro	F1-score Weighted	Precision Micro	Precision Macro	Precision Weighted	Recall Micro	Recall Macro	Recall Weighted	Jaccard Micro	Jaccard Macro	Jaccard Weighted
BTOP	99.97	99.94	99.97	99.97	99.97	99.97	100.00	99.98	99.97	99.94	99.97	99.95	99.94	99.95
HiTaC	99.97	99.94	99.97	99.97	99.97	99.97	100.00	99.97	99.97	99.94	99.97	99.95	99.94	99.95
HiTaC_Filter	99.95	99.94	99.95	99.55	99.96	99.95	99.58	99.97	99.95	99.52	99.95	99.90	99.52	99.92
Microclass	99.95	99.92	99.95	99.93	99.95	99.95	99.95	99.95	99.95	99.92	99.95	99.90	99.88	99.90
KTOP	99.92	99.92	99.92	99.93	99.92	99.92	99.95	99.93	99.92	99.92	99.92	99.85	99.87	99.85
NBC50	99.92	99.92	99.92	99.93	99.92	99.92	99.95	99.93	99.92	99.92	99.92	99.85	99.87	99.85
RDP50	99.92	99.92	99.92	99.93	99.92	99.92	99.95	99.93	99.92	99.92	99.92	99.85	99.87	99.85
TOP	99.92	99.92	99.92	99.87	99.93	99.92	99.85	99.93	99.92	99.92	99.92	99.85	99.77	99.86
Q2_SK	99.90	99.87	99.90	99.49	99.92	99.90	99.53	99.95	99.90	99.46	99.90	99.80	99.41	99.85
NBC80	99.87	99.87	99.87	99.51	99.92	99.87	99.58	99.97	99.87	99.45	99.87	99.75	99.45	99.85
SINTAX50	99.87	99.87	99.87	99.51	99.92	99.87	99.58	99.97	99.87	99.45	99.87	99.75	99.45	99.85
SPINGO	99.87	99.87	99.87	99.51	99.92	99.87	99.58	99.97	99.87	99.45	99.87	99.75	99.45	99.85
RDP80	99.87	99.87	99.87	99.49	99.91	99.87	99.53	99.95	99.87	99.45	99.87	99.75	99.41	99.83
SINTAX80	99.80	99.73	99.80	99.44	99.88	99.80	99.58	99.97	99.80	99.32	99.80	99.60	99.32	99.77
Q1	99.06	98.67	99.06	98.18	99.21	99.06	98.24	99.42	99.06	98.27	99.06	98.14	97.36	98.52
CT1	97.95	91.67	97.95	90.84	97.99	97.95	91.94	98.31	97.95	90.91	97.95	95.98	88.42	96.60
Metaxa2	96.22	84.42	96.22	86.31	97.59	96.22	89.67	99.29	96.22	84.07	96.22	92.72	84.07	96.20
Q2_VS	96.10	85.90	96.10	85.98	96.45	96.10	87.93	97.23	96.10	85.19	96.10	92.49	82.96	94.29
Q2_BLAST	95.56	84.96	95.56	85.19	96.09	95.56	87.84	97.28	95.56	84.26	95.56	91.50	81.85	93.75
BLCA	95.13	95.48	95.13	96.60	97.08	95.13	99.17	99.91	95.13	95.08	95.13	90.72	95.08	95.12
CT2	93.81	77.40	93.81	78.02	94.44	93.81	82.26	96.00	93.81	76.45	93.81	88.35	73.78	91.58
KNN	80.84	45.20	80.84	48.58	85.31	80.84	55.78	92.72	80.84	45.01	80.84	67.84	45.01	80.81

Table S85. Machine learning metrics computed for the dataset SP RDP ITS 99 at the genus level.

Method	Accuracy	Balanced Accuracy	F1-score Micro	F1-score Macro	F1-score Weighted	Precision Micro	Precision Macro	Precision Weighted	Recall Micro	Recall Macro	Recall Weighted	Jaccard Micro	Jaccard Macro	Jaccard Weighted
TOP	99.52	99.64	99.52	99.05	99.58	99.52	99.11	99.69	99.52	99.04	99.52	99.04	98.89	99.27
HiTaC	99.49	99.48	99.49	99.08	99.51	99.49	99.15	99.61	99.49	99.12	99.49	98.99	98.88	99.17
Microclass	99.44	99.45	99.44	98.96	99.48	99.44	99.04	99.58	99.44	98.97	99.44	98.89	98.75	99.11
BTOP	99.37	99.49	99.37	98.86	99.42	99.37	98.89	99.51	99.37	98.89	99.37	98.76	98.66	99.02
KTOP	99.37	99.42	99.37	98.82	99.43	99.37	98.90	99.55	99.37	98.83	99.37	98.74	98.60	99.02
RDP50	99.32	99.38	99.32	98.88	99.39	99.32	99.02	99.58	99.32	98.90	99.32	98.64	98.64	98.97
NBC50	99.29	99.37	99.29	98.86	99.35	99.29	98.98	99.52	99.29	98.89	99.29	98.59	98.61	98.91
Q2_SK	99.24	99.36	99.24	99.15	99.38	99.24	99.30	99.64	99.24	99.12	99.24	98.49	98.91	98.95
SINTAX50	99.21	99.35	99.21	98.89	99.39	99.21	99.05	99.69	99.21	98.87	99.21	98.44	98.64	98.96
SPINGO	98.99	99.24	98.99	99.26	99.27	98.99	99.55	99.73	98.99	99.12	98.99	97.99	99.03	98.79
RDP80	98.86	99.12	98.86	99.03	99.23	98.86	99.30	99.76	98.86	98.88	98.86	97.74	98.79	98.71
NBC80	98.81	99.08	98.81	99.01	99.22	98.81	99.31	99.79	98.81	98.84	98.81	97.65	98.75	98.68
HiTaC_Filter	98.78	98.83	98.78	98.99	99.19	98.78	99.47	99.80	98.78	98.71	98.78	97.60	98.68	98.68
SINTAX80	97.97	98.08	97.97	98.30	98.60	97.97	99.03	99.72	97.97	97.96	97.97	96.02	97.96	97.90
Q1	95.54	90.51	95.54	88.93	95.49	95.54	89.74	96.04	95.54	89.11	95.54	91.46	87.22	93.34
BLCA	94.32	95.26	94.32	95.69	95.99	94.32	97.30	99.07	94.32	94.91	94.32	89.25	94.75	94.16
CT1	92.22	79.41	92.22	75.50	91.66	92.22	75.87	92.05	92.22	76.72	92.22	85.56	72.74	88.53
Metaxa2	89.05	73.05	89.05	74.58	91.32	89.05	77.84	94.73	89.05	72.78	89.05	80.26	72.72	88.90
Q2_VS	81.42	58.70	81.42	57.74	81.20	81.42	59.06	82.86	81.42	58.21	81.42	68.66	54.94	76.70
Q2_BLAST	80.15	55.87	80.15	55.13	79.88	80.15	56.89	81.52	80.15	55.41	80.15	66.88	51.89	74.96
CT2	77.08	47.32	77.08	46.66	76.91	77.08	48.79	79.21	77.08	46.70	77.08	62.71	43.72	72.02
KNN	47.98	15.58	47.98	17.03	53.01	47.98	20.14	62.89	47.98	15.56	47.98	31.57	15.56	47.96

Table S86. Machine learning metrics computed for the dataset SP RDP ITS 99 at the species level.

Method	Accuracy	Balanced Accuracy	F1-score Micro	F1-score Macro	F1-score Weighted	Precision Micro	Precision Macro	Precision Weighted	Recall Micro	Recall Macro	Recall Weighted	Jaccard Micro	Jaccard Macro	Jaccard Weighted
HiTaC	72.98	77.61	72.98	66.47	71.65	72.98	66.12	71.59	72.98	67.79	72.98	57.45	65.69	70.56
BTOP	72.59	76.78	72.59	65.60	71.13	72.59	65.17	71.02	72.59	67.05	72.59	56.97	64.78	70.03
Microclass	72.32	76.87	72.32	65.90	70.72	72.32	65.42	70.32	72.32	67.38	72.32	56.64	65.15	69.72
TOP	72.29	76.98	72.29	65.95	70.96	72.29	65.64	70.92	72.29	67.25	72.29	56.61	65.16	69.86
KTOP	71.63	76.35	71.63	65.39	70.06	71.63	64.89	69.70	71.63	66.89	71.63	55.81	64.56	68.96
NBC50	68.59	73.81	68.59	63.59	66.98	68.59	63.06	66.51	68.59	65.10	68.59	52.20	62.85	66.05
RDP50	68.29	73.51	68.29	63.36	66.73	68.29	62.86	66.30	68.29	64.82	68.29	51.85	62.64	65.81
SINTAX50	68.24	73.34	68.24	65.11	67.26	68.24	64.80	67.13	68.24	66.10	68.24	51.79	64.51	66.50
Q2_SK	66.89	72.37	66.89	63.74	65.80	66.89	63.35	65.48	66.89	64.81	66.89	50.26	63.21	65.15
SPINGO	65.73	71.03	65.73	64.70	65.31	65.73	64.65	65.56	65.73	65.24	65.73	48.95	64.23	64.68
NBC80	64.06	69.53	64.06	62.43	63.15	64.06	62.12	62.96	64.06	63.35	64.06	47.12	61.96	62.60
RDP80	63.90	69.36	63.90	62.49	63.03	63.90	62.18	62.82	63.90	63.37	63.90	46.95	62.04	62.51
HiTaC_Filter	63.45	68.82	63.45	63.45	63.15	63.45	63.45	63.47	63.45	63.89	63.45	46.46	63.07	62.62
BLCA	63.07	67.98	63.07	60.21	62.32	63.07	60.01	62.43	63.07	61.10	63.07	46.06	59.58	61.47
SINTAX80	57.90	63.39	57.90	59.76	57.73	57.90	59.79	58.01	57.90	60.06	57.90	40.74	59.44	57.31
Q1	36.91	39.50	36.91	32.19	34.93	36.91	31.41	34.14	36.91	34.02	36.91	22.63	31.33	33.90
CT1	27.10	28.42	27.10	21.62	24.50	27.10	20.67	23.47	27.10	23.93	27.10	15.67	20.59	23.28
Metaxa2	23.12	24.39	23.12	22.58	22.69	23.12	22.45	22.71	23.12	23.05	23.12	13.07	22.27	22.32
Q2_VS	7.25	8.11	7.25	7.81	7.11	7.25	7.74	7.04	7.25	7.97	7.25	3.76	7.74	7.04
Q2_BLAST	5.75	6.43	5.75	6.22	5.65	5.75	6.17	5.61	5.75	6.33	5.75	2.96	6.17	5.61
CT2	2.94	3.24	2.94	3.18	2.90	2.94	3.16	2.89	2.94	3.21	2.94	1.49	3.16	2.89
KNN	0.00	0.00	0.00	0.00	0.00	0.00	0.00	0.00	0.00	0.00	0.00	0.00	0.00	0.00

Table S87. Machine learning metrics computed for the dataset SP RDP ITS 100 at the phylum level.

Method	Accuracy	Balanced Accuracy	F1-score Micro	F1-score Macro	F1-score Weighted	Precision Micro	Precision Macro	Precision Weighted	Recall Micro	Recall Macro	Recall Weighted	Jaccard Micro	Jaccard Macro	Jaccard Weighted
BTOP	100.00	100.00	100.00	100.00	100.00	100.00	100.00	100.00	100.00	100.00	100.00	100.00	100.00	100.00
CT1	100.00	100.00	100.00	100.00	100.00	100.00	100.00	100.00	100.00	100.00	100.00	100.00	100.00	100.00
CT2	100.00	100.00	100.00	100.00	100.00	100.00	100.00	100.00	100.00	100.00	100.00	100.00	100.00	100.00
HiTaC	100.00	100.00	100.00	100.00	100.00	100.00	100.00	100.00	100.00	100.00	100.00	100.00	100.00	100.00
HiTaC_Filter	100.00	100.00	100.00	100.00	100.00	100.00	100.00	100.00	100.00	100.00	100.00	100.00	100.00	100.00
KTOP	100.00	100.00	100.00	100.00	100.00	100.00	100.00	100.00	100.00	100.00	100.00	100.00	100.00	100.00
Metaxa2	100.00	100.00	100.00	100.00	100.00	100.00	100.00	100.00	100.00	100.00	100.00	100.00	100.00	100.00
Microclass	100.00	100.00	100.00	100.00	100.00	100.00	100.00	100.00	100.00	100.00	100.00	100.00	100.00	100.00
Q1	100.00	100.00	100.00	100.00	100.00	100.00	100.00	100.00	100.00	100.00	100.00	100.00	100.00	100.00
Q2_BLAST	100.00	100.00	100.00	100.00	100.00	100.00	100.00	100.00	100.00	100.00	100.00	100.00	100.00	100.00
Q2_SK	100.00	100.00	100.00	100.00	100.00	100.00	100.00	100.00	100.00	100.00	100.00	100.00	100.00	100.00
Q2_VS	100.00	100.00	100.00	100.00	100.00	100.00	100.00	100.00	100.00	100.00	100.00	100.00	100.00	100.00
RDP50	100.00	100.00	100.00	100.00	100.00	100.00	100.00	100.00	100.00	100.00	100.00	100.00	100.00	100.00
RDP80	100.00	100.00	100.00	100.00	100.00	100.00	100.00	100.00	100.00	100.00	100.00	100.00	100.00	100.00
SINTAX50	100.00	100.00	100.00	100.00	100.00	100.00	100.00	100.00	100.00	100.00	100.00	100.00	100.00	100.00
SINTAX80	100.00	100.00	100.00	100.00	100.00	100.00	100.00	100.00	100.00	100.00	100.00	100.00	100.00	100.00
TOP	100.00	100.00	100.00	100.00	100.00	100.00	100.00	100.00	100.00	100.00	100.00	100.00	100.00	100.00
KNN	99.81	53.42	99.81	48.15	99.88	99.81	50.00	99.96	99.81	46.74	99.81	99.63	46.74	99.81
BLCA	0.00	0.00	0.00	0.00	0.00	0.00	0.00	0.00	0.00	0.00	0.00	0.00	0.00	0.00
SPINGO	0.00	0.00	0.00	0.00	0.00	0.00	0.00	0.00	0.00	0.00	0.00	0.00	0.00	0.00

Table S88. Machine learning metrics computed for the dataset SP RDP ITS 100 at the class level.

Method	Accuracy	Balanced Accuracy	F1-score Micro	F1-score Macro	F1-score Weighted	Precision Micro	Precision Macro	Precision Weighted	Recall Micro	Recall Macro	Recall Weighted	Jaccard Micro	Jaccard Macro	Jaccard Weighted
BTOP	100.00	100.00	100.00	100.00	100.00	100.00	100.00	100.00	100.00	100.00	100.00	100.00	100.00	100.00
HiTaC	100.00	100.00	100.00	100.00	100.00	100.00	100.00	100.00	100.00	100.00	100.00	100.00	100.00	100.00
HiTaC_Filter	100.00	100.00	100.00	100.00	100.00	100.00	100.00	100.00	100.00	100.00	100.00	100.00	100.00	100.00
KTOP	100.00	100.00	100.00	100.00	100.00	100.00	100.00	100.00	100.00	100.00	100.00	100.00	100.00	100.00
Microclass	100.00	100.00	100.00	100.00	100.00	100.00	100.00	100.00	100.00	100.00	100.00	100.00	100.00	100.00
Q2_SK	100.00	100.00	100.00	100.00	100.00	100.00	100.00	100.00	100.00	100.00	100.00	100.00	100.00	100.00
RDP50	100.00	100.00	100.00	100.00	100.00	100.00	100.00	100.00	100.00	100.00	100.00	100.00	100.00	100.00
RDP80	100.00	100.00	100.00	100.00	100.00	100.00	100.00	100.00	100.00	100.00	100.00	100.00	100.00	100.00
SINTAX50	100.00	100.00	100.00	100.00	100.00	100.00	100.00	100.00	100.00	100.00	100.00	100.00	100.00	100.00
SINTAX80	100.00	100.00	100.00	100.00	100.00	100.00	100.00	100.00	100.00	100.00	100.00	100.00	100.00	100.00
TOP	100.00	100.00	100.00	100.00	100.00	100.00	100.00	100.00	100.00	100.00	100.00	100.00	100.00	100.00
Q1	99.99	100.00	99.99	100.00	99.99	99.99	100.00	99.99	99.99	100.00	99.99	99.99	99.99	99.99
CT1	99.93	99.97	99.93	99.97	99.93	99.93	99.97	99.93	99.93	99.97	99.93	99.86	99.94	99.86
Metaxa2	99.88	96.39	99.88	93.41	99.94	99.88	93.55	99.99	99.88	93.28	99.88	99.76	93.28	99.88
Q2_VS	99.74	99.64	99.74	96.46	99.75	99.74	96.51	99.77	99.74	96.42	99.74	99.48	96.16	99.51
Q2_BLAST	99.73	99.63	99.73	96.46	99.75	99.73	96.51	99.77	99.73	96.42	99.73	99.47	96.16	99.50
CT2	99.67	99.46	99.67	96.34	99.69	99.67	96.44	99.70	99.67	96.25	99.67	99.34	95.92	99.38
KNN	98.15	57.88	98.15	57.92	98.91	98.15	61.29	99.77	98.15	56.01	98.15	96.37	56.01	98.15
BLCA	0.00	0.00	0.00	0.00	0.00	0.00	0.00	0.00	0.00	0.00	0.00	0.00	0.00	0.00
SPINGO	0.00	0.00	0.00	0.00	0.00	0.00	0.00	0.00	0.00	0.00	0.00	0.00	0.00	0.00

Table S89. Machine learning metrics computed for the dataset SP RDP ITS 100 at the order level.

Method	Accuracy	Balanced Accuracy	F1-score Micro	F1-score Macro	F1-score Weighted	Precision Micro	Precision Macro	Precision Weighted	Recall Micro	Recall Macro	Recall Weighted	Jaccard Micro	Jaccard Macro	Jaccard Weighted
BTOP	100.00	100.00	100.00	100.00	100.00	100.00	100.00	100.00	100.00	100.00	100.00	100.00	100.00	100.00
HiTaC	100.00	100.00	100.00	100.00	100.00	100.00	100.00	100.00	100.00	100.00	100.00	100.00	100.00	100.00
HiTaC_Filter	100.00	100.00	100.00	100.00	100.00	100.00	100.00	100.00	100.00	100.00	100.00	100.00	100.00	100.00
KTOP	100.00	100.00	100.00	100.00	100.00	100.00	100.00	100.00	100.00	100.00	100.00	100.00	100.00	100.00
Microclass	100.00	100.00	100.00	100.00	100.00	100.00	100.00	100.00	100.00	100.00	100.00	100.00	100.00	100.00
Q2_SK	100.00	100.00	100.00	100.00	100.00	100.00	100.00	100.00	100.00	100.00	100.00	100.00	100.00	100.00
RDP50	100.00	100.00	100.00	100.00	100.00	100.00	100.00	100.00	100.00	100.00	100.00	100.00	100.00	100.00
RDP80	100.00	100.00	100.00	100.00	100.00	100.00	100.00	100.00	100.00	100.00	100.00	100.00	100.00	100.00
SINTAX50	100.00	100.00	100.00	100.00	100.00	100.00	100.00	100.00	100.00	100.00	100.00	100.00	100.00	100.00
SINTAX80	100.00	100.00	100.00	100.00	100.00	100.00	100.00	100.00	100.00	100.00	100.00	100.00	100.00	100.00
TOP	100.00	100.00	100.00	100.00	100.00	100.00	100.00	100.00	100.00	100.00	100.00	100.00	100.00	100.00
Q1	99.96	98.99	99.96	97.98	99.96	99.96	97.95	99.96	99.96	98.02	99.96	99.93	97.93	99.93
CT1	99.79	96.50	99.79	95.55	99.79	99.79	95.59	99.79	99.79	95.55	99.79	99.59	95.07	99.60
Metaxa2	99.69	94.19	99.69	93.67	99.83	99.69	94.12	99.97	99.69	93.26	99.69	99.39	93.26	99.69
Q2_VS	99.05	95.17	99.05	94.94	99.18	99.05	96.01	99.36	99.05	94.24	99.05	98.11	93.32	98.48
Q2_BLAST	98.96	92.88	98.96	92.73	99.11	98.96	93.98	99.31	98.96	91.97	98.96	97.94	90.97	98.35
CT2	98.73	89.11	98.73	89.55	98.91	98.73	91.94	99.17	98.73	88.24	98.73	97.49	87.19	98.00
KNN	94.37	54.20	94.37	56.59	96.34	94.37	60.78	98.68	94.37	53.67	94.37	89.34	53.67	94.37
BLCA	0.00	0.00	0.00	0.00	0.00	0.00	0.00	0.00	0.00	0.00	0.00	0.00	0.00	0.00
SPINGO	0.00	0.00	0.00	0.00	0.00	0.00	0.00	0.00	0.00	0.00	0.00	0.00	0.00	0.00

Table S90. Machine learning metrics computed for the dataset SP RDP ITS 100 at the family level.

Method	Accuracy	Balanced Accuracy	F1-score Micro	F1-score Macro	F1-score Weighted	Precision Micro	Precision Macro	Precision Weighted	Recall Micro	Recall Macro	Recall Weighted	Jaccard Micro	Jaccard Macro	Jaccard Weighted
BTOP	100.00	100.00	100.00	100.00	100.00	100.00	100.00	100.00	100.00	100.00	100.00	100.00	100.00	100.00
HiTaC	100.00	100.00	100.00	100.00	100.00	100.00	100.00	100.00	100.00	100.00	100.00	100.00	100.00	100.00
HiTaC_Filter	100.00	100.00	100.00	100.00	100.00	100.00	100.00	100.00	100.00	100.00	100.00	100.00	100.00	100.00
KTOP	100.00	100.00	100.00	100.00	100.00	100.00	100.00	100.00	100.00	100.00	100.00	100.00	100.00	100.00
Microclass	100.00	100.00	100.00	100.00	100.00	100.00	100.00	100.00	100.00	100.00	100.00	100.00	100.00	100.00
SINTAX50	100.00	100.00	100.00	100.00	100.00	100.00	100.00	100.00	100.00	100.00	100.00	100.00	100.00	100.00
SPINGO	100.00	100.00	100.00	100.00	100.00	100.00	100.00	100.00	100.00	100.00	100.00	100.00	100.00	100.00
TOP	100.00	100.00	100.00	100.00	100.00	100.00	100.00	100.00	100.00	100.00	100.00	100.00	100.00	100.00
Q2_SK	99.99	100.00	99.99	99.65	100.00	99.99	99.66	100.00	99.99	99.65	99.99	99.99	99.65	99.99
RDP50	99.99	100.00	99.99	99.65	100.00	99.99	99.66	100.00	99.99	99.65	99.99	99.99	99.65	99.99
RDP80	99.99	99.82	99.99	99.54	99.99	99.99	99.66	100.00	99.99	99.48	99.99	99.98	99.48	99.99
SINTAX80	99.98	99.96	99.98	99.63	99.99	99.98	99.66	100.00	99.98	99.61	99.98	99.95	99.61	99.98
Q1	99.83	98.12	99.83	97.75	99.82	99.83	97.73	99.81	99.83	97.79	99.83	99.66	97.58	99.68
CT1	99.47	94.39	99.47	94.10	99.45	99.47	94.23	99.45	99.47	94.06	99.47	98.95	93.47	99.02
Metaxa2	99.22	92.31	99.22	92.64	99.54	99.22	93.45	99.88	99.22	91.99	99.22	98.44	91.99	99.22
Q2_VS	96.37	86.84	96.37	87.49	96.84	96.37	89.87	97.68	96.37	86.54	96.37	92.99	84.68	94.68
Q2_BLAST	96.14	85.71	96.14	86.55	96.72	96.14	89.49	97.72	96.14	85.41	96.14	92.58	83.29	94.40
CT2	95.76	81.91	95.76	84.00	96.46	95.76	88.57	97.57	95.76	81.63	95.76	91.86	79.69	93.92
KNN	84.75	45.98	84.75	49.49	89.04	84.75	56.21	95.75	84.75	45.82	84.75	73.54	45.82	84.75
BLCA	0.00	0.00	0.00	0.00	0.00	0.00	0.00	0.00	0.00	0.00	0.00	0.00	0.00	0.00

Table S91. Machine learning metrics computed for the dataset SP RDP ITS 100 at the genus level.

Method	Accuracy	Balanced Accuracy	F1-score Micro	F1-score Macro	F1-score Weighted	Precision Micro	Precision Macro	Precision Weighted	Recall Micro	Recall Macro	Recall Weighted	Jaccard Micro	Jaccard Macro	Jaccard Weighted
BTOP	100.00	100.00	100.00	100.00	100.00	100.00	100.00	100.00	100.00	100.00	100.00	100.00	100.00	100.00
HiTaC	100.00	100.00	100.00	100.00	100.00	100.00	100.00	100.00	100.00	100.00	100.00	100.00	100.00	100.00
KTOP	100.00	100.00	100.00	100.00	100.00	100.00	100.00	100.00	100.00	100.00	100.00	100.00	100.00	100.00
Microclass	99.99	99.99	99.99	100.00	99.99	99.99	100.00	99.99	99.99	99.99	99.99	99.99	99.99	99.99
TOP	99.99	99.99	99.99	99.99	99.99	99.99	100.00	99.99	99.99	99.99	99.99	99.99	99.99	99.99
SINTAX50	99.99	99.98	99.99	99.91	100.00	99.99	99.92	100.00	99.99	99.90	99.99	99.99	99.90	99.99
SPINGO	99.98	99.97	99.98	99.90	99.99	99.98	99.92	100.00	99.98	99.89	99.98	99.96	99.89	99.98
Q2_SK	99.97	99.87	99.97	99.80	99.97	99.97	99.81	99.98	99.97	99.79	99.97	99.94	99.77	99.95
RDP50	99.96	99.88	99.96	99.80	99.96	99.96	99.81	99.96	99.96	99.79	99.96	99.93	99.77	99.93
HiTaC_Filter	99.95	99.89	99.95	99.82	99.97	99.95	99.83	99.99	99.95	99.81	99.95	99.90	99.81	99.95
RDP80	99.91	99.70	99.91	99.66	99.94	99.91	99.73	99.97	99.91	99.62	99.91	99.83	99.59	99.90
SINTAX80	99.76	99.53	99.76	99.58	99.86	99.76	99.75	99.99	99.76	99.45	99.76	99.53	99.45	99.76
Q1	98.97	95.51	98.97	95.49	98.92	98.97	95.83	98.96	98.97	95.43	98.97	97.97	94.69	98.21
CT1	98.16	89.35	98.16	88.94	97.95	98.16	89.02	97.88	98.16	89.28	98.16	96.38	87.59	96.83
Metaxa2	97.68	87.58	97.68	88.28	98.35	97.68	89.35	99.13	97.68	87.51	97.68	95.47	87.51	97.68
Q2_VS	84.27	57.74	84.27	57.93	84.36	84.27	60.28	86.12	84.27	57.70	84.27	72.82	54.91	79.63
Q2_BLAST	83.62	54.73	83.62	55.23	83.76	83.62	58.25	85.78	83.62	54.69	83.62	71.85	51.70	78.68
CT2	82.90	51.40	82.90	53.84	83.51	82.90	60.63	86.41	82.90	51.36	82.90	70.79	48.51	77.99
KNN	57.61	17.43	57.61	19.13	62.89	57.61	23.20	73.90	57.61	17.42	57.61	40.46	17.42	57.61
BLCA	0.00	0.00	0.00	0.00	0.00	0.00	0.00	0.00	0.00	0.00	0.00	0.00	0.00	0.00

Table S92. Machine learning metrics computed for the dataset SP RDP ITS 100 at the species level.

Method	Accuracy	Balanced Accuracy	F1-score Micro	F1-score Macro	F1-score Weighted	Precision Micro	Precision Macro	Precision Weighted	Recall Micro	Recall Macro	Recall Weighted	Jaccard Micro	Jaccard Macro	Jaccard Weighted
BTOP	99.99	99.99	99.99	99.99	99.99	99.99	99.99	100.00	99.99	99.99	99.99	99.99	99.99	99.99
HiTaC	99.97	99.99	99.97	99.98	99.97	99.97	99.98	99.98	99.97	99.99	99.97	99.94	99.97	99.95
TOP	99.56	99.50	99.56	99.43	99.51	99.56	99.50	99.60	99.56	99.50	99.56	99.12	99.24	99.28
KTOP	99.45	99.48	99.45	99.37	99.39	99.45	99.45	99.54	99.45	99.48	99.45	98.90	99.14	99.11
Microclass	99.38	99.14	99.38	99.07	99.25	99.38	99.13	99.28	99.38	99.14	99.38	98.77	98.84	98.94
HiTaC_Filter	97.78	97.80	97.78	98.12	98.24	97.78	98.75	99.10	97.78	97.78	97.78	95.66	97.78	97.78
SINTAX50	97.50	97.08	97.50	97.32	97.83	97.50	98.00	98.70	97.50	97.07	97.50	95.13	96.86	97.22
SPINGO	96.77	96.43	96.77	97.01	97.52	96.77	98.16	98.97	96.77	96.42	96.77	93.74	96.42	96.77
RDP50	96.19	93.95	96.19	93.57	95.47	96.19	93.81	95.50	96.19	93.94	96.19	92.67	92.69	94.30
Q2_SK	95.33	93.22	95.33	93.51	95.60	95.33	94.39	96.53	95.33	93.21	95.33	91.09	92.80	94.72
RDP80	93.02	89.86	93.02	90.09	93.13	93.02	91.08	94.13	93.02	89.85	93.02	86.96	89.20	92.00
SINTAX80	86.18	85.13	86.18	86.39	87.82	86.18	89.10	91.41	86.18	85.12	86.18	75.71	85.11	86.16
Q1	83.71	73.71	83.71	72.29	81.98	83.71	72.42	82.05	83.71	73.70	83.71	71.99	69.80	78.91
CT1	82.65	70.33	82.65	68.46	80.39	82.65	68.33	80.17	82.65	70.32	82.65	70.44	65.78	77.14
Metaxa2	77.98	66.12	77.98	67.30	79.50	77.98	69.70	82.59	77.98	66.11	77.98	63.90	66.10	77.97
Q2_VS	10.90	9.90	10.90	9.81	10.70	10.90	10.00	11.01	10.90	9.90	10.90	5.76	9.50	10.19
Q2_BLAST	9.27	8.10	9.27	8.08	9.17	9.27	8.39	9.60	9.27	8.10	9.27	4.86	7.76	8.65
CT2	8.53	6.86	8.53	7.80	9.49	8.53	9.83	11.79	8.53	6.86	8.53	4.45	6.61	8.01
KNN	0.07	0.01	0.07	0.01	0.07	0.07	0.01	0.08	0.07	0.01	0.07	0.03	0.01	0.07
BLCA	0.00	0.00	0.00	0.00	0.00	0.00	0.00	0.00	0.00	0.00	0.00	0.00	0.00	0.00

References

1. RC Edgar, Accuracy of taxonomy prediction for 16s rrna and fungal its sequences. *PeerJ* **6**, e4652 (2018).
2. EW Sayers, et al., Database resources of the national center for biotechnology information. *Nucleic acids research* **40**, D13–D25 (2012).
3. JR Cole, et al., Ribosomal database project: data and tools for high throughput rrna analysis. *Nucleic acids research* **42**, D633–D642 (2014).
4. V Deshpande, et al., Fungal identification using a bayesian classifier and the warcup training set of internal transcribed spacer sequences. *Mycologia* **108**, 1–5 (2016).
5. U Kõljalg, et al., Unite: a database providing web-based methods for the molecular identification of ectomycorrhizal fungi. *New Phytol.* **166**, 1063–1068 (2005).
6. F Pedregosa, et al., Scikit-learn: Machine learning in python. *J. machine learning research* **12**, 2825–2830 (2011).
7. FM Miranda, N Köhnecke, BY Renard, Hiclass: a python library for local hierarchical classification compatible with scikit-learn. *J. Mach. Learn. Res.* **24**, 1–17 (2023).
8. J Köster, S Rahmann, Snakemake—a scalable bioinformatics workflow engine. *Bioinformatics* **28**, 2520–2522 (2012).
9. X Gao, H Lin, K Revanna, Q Dong, A bayesian taxonomic classification method for 16s rrna gene sequences with improved species-level accuracy. *BMC bioinformatics* **18**, 247 (2017).
10. SF Altschul, W Gish, W Miller, EW Myers, DJ Lipman, Basic local alignment search tool. *J. molecular biology* **215**, 403–410 (1990).
11. PD Schloss, et al., Introducing mothur: open-source, platform-independent, community-supported software for describing and comparing microbial communities. *Appl. Environ. Microbiol.* **75**, 7537–7541 (2009).
12. J Bengtsson-Palme, et al., Metaxa2: improved identification and taxonomic classification of small and large subunit rrna in metagenomic data. *Mol. Ecol. Resour.* **15**, 1403–1414 (2015).
13. KH Liland, H Vinje, L Snipen, microclass: an r-package for 16s taxonomy classification. *BMC bioinformatics* **18**, 172 (2017).
14. JG Caporaso, et al., Qiime allows analysis of high–throughput community sequencing data. *Nat. Methods* **7**, 335 (2010).
15. NA Bokulich, et al., Optimizing taxonomic classification of marker-gene amplicon sequences with qiime 2's q2-feature-classifier plugin. *Microbiome* **6**, 1–17 (2018).
16. Q Wang, GM Garrity, JM Tiedje, JR Cole, Naive bayesian classifier for rapid assignment of rrna sequences into the new bacterial taxonomy. *Appl. Environ. Microbiol.* **73**, 5261–5267 (2007).
17. R Edgar, Sintax: a simple non-bayesian taxonomy classifier for 16s and its sequences. *BioRxiv* p. 074161 (2016).
18. G Allard, FJ Ryan, IB Jeffery, MJ Claesson, Spingo: a rapid species-classifier for microbial amplicon sequences. *BMC bioinformatics* **16**, 324 (2015).
